# A survey of strategies for the early diagnosis of hearing loss in infants: a scoping review

**DOI:** 10.1590/2317-1782/e20250375en

**Published:** 2026-07-31

**Authors:** Allan Dayner Silva Lopes, Antônio Pereira de Carvalho, Sanmara de Andrade Silva, Rodrigo Oliveira da Fonseca, Maiara Cristine Oliveira de Almeida, Maria Helena Medeiros de Sá Lima Lucena, Karinna Veríssimo Meira Taveira, Hannalice Gottschalck Cavalcanti

**Affiliations:** 1 Programa Associado de Pós-graduação em Fonoaudiologia, Universidade Federal da Paraíba – UFPB - João Pessoa (PB), Brasil.; 2 Departamento de Fonoaudiologia, Universidade Federal da Paraíba – UFPB - João Pessoa (PB), Brasil.; 3 Programa Associado de Pós-graduação em Fonoaudiologia, Universidade Federal do Rio Grande do Norte Natal – UFRN - Natal (RN), Brasil.; 4 Departamento de Morfologia, Universidade Federal do Rio Grande do Norte Natal – UFRN - Natal (RN), Brasil.

**Keywords:** Neonatal Screening, Hearing Loss, Infant, Early Diagnosis, Scoping Review

## Abstract

**Purpose:**

To map the strategies used for the early diagnosis of hearing loss in infants.

**Research strategies:**

A comprehensive literature search was conducted in PubMed/MEDLINE, Scopus, Embase, Web of Science, LILACS, Google Scholar, and ProQuest, with no restrictions on language or publication period. The search was initially performed in October 2024 and updated in January 2026. Controlled descriptors (DeCS/MeSH) and keywords related to hearing loss, newborn hearing screening, and early diagnosis were used. The protocol was registered in the Open Science Framework.

**Selection criteria:**

Primary studies addressing strategies for the early diagnosis of hearing loss in children up to two years of age were included. Secondary studies, case reports, guidelines, duplicate publications, and studies without full-text availability were excluded. Study selection was performed independently by reviewers, with disagreements resolved by consensus.

**Data analysis:**

Extracted data included study design, population, diagnostic instruments, strategies, and care setting. A descriptive and narrative synthesis was conducted, grouping evidence according to diagnostic approaches.

**Results:**

Of the 16,946 records identified, 58 studies published between 1991 and 2025 met the eligibility criteria. Universal Newborn Hearing Screening (UNHS) predominated, particularly protocols combining otoacoustic emissions and automated auditory brainstem response. Complementary strategies included two-stage screening, early retesting, community-based programs, genetic screening, and additional electrophysiological assessments. Variability in protocol implementation and persistent structural inequalities were observed.

**Conclusion:**

UNHS remains the cornerstone of early diagnosis of hearing loss in infants, with effectiveness dependent on well-organized care pathways and integration between screening, diagnosis, and follow-up.

## INTRODUCTION

Hearing plays a central role in child development and is directly related to language acquisition, communication, and cognitive and social skills^(1,2)^. Hearing deprivation during the first years of life may lead to long-term educational, emotional, and social impairments, making early identification and intervention essential to mitigate these impacts^(1-3)^.

In this context, Universal Newborn Hearing Screening (UNHS) programs have become an important public health policy in several countries, aiming to promote the early detection of hearing loss^(4,5)^. However, structural, organizational, and contextual challenges—more prominently highlighted during the COVID-19 pandemic—have compromised the continuity of these programs, resulting in delays in diagnosis, intervention, and follow-up care^(5-7)^.

National and international guidelines recommend the combined use of Otoacoustic Emissions (OAE) and Automated Auditory Brainstem Response (AABR) as the gold-standard protocol for newborn hearing screening, preferably to be performed within the first 48 hours of life or up to 30 days after birth^(2,6,8-10)^. However, the existence of formal recommendations does not ensure their homogeneous implementation in clinical practice. Differences among healthcare systems, between public and private sectors, as well as failures in the integration of healthcare networks, result in variations in diagnostic pathways and in the effectiveness of follow-up care^(8-11)^.

The literature suggests that strategies for the early diagnosis of hearing loss in infants remain fragmented, often focusing on specific technologies or populations without comprehensively considering organizational, socioeconomic, and geographic factors that influence access to services and continuity of care^(8,12-14)^. This heterogeneity hinders comparisons across contexts and limits the identification of patterns that could support the improvement of clinical practices and public hearing health policies^(9,13,15)^.

Given the epidemiological relevance of childhood hearing loss and the persistent challenges related to its early detection and intervention^(12,13,16-19)^, a gap remains in the literature regarding a critical synthesis of the strategies employed for early diagnosis, encompassing not only the tests used but also care pathways, protocols, educational interventions, and health policies. Therefore, the objective of this scoping review was to map the strategies used for the early diagnosis of hearing loss in infants by gathering and synthesizing the available scientific evidence in order to contribute to the improvement of clinical practices and the strengthening of public policies for child hearing health.

## METHODS

This scoping review was conducted in accordance with the methodological recommendations of the Joanna Briggs Institute (JBI)^(20)^ and reported following the Preferred Reporting Items for Systematic Reviews and Meta-Analyses Extension for Scoping Reviews (PRISMA-ScR) checklist^(21)^. The study protocol was previously registered on the Open Science Framework (OSF)^(22)^. 

### Eligibility criteria

The research question was formulated based on the PCC strategy (Population, Concept, and Context), defined as follows:

– infants (0–2 years);
  – strategies used for the early diagnosis of hearing loss;
    – hearing healthcare services and settings.

Thus, the guiding research question was: “What strategies have been used for the early diagnosis of hearing loss in infants?”

### Inclusion criteria

Studies addressing strategies, protocols, programs, or methods applied to the early diagnosis of hearing impairment in children up to two years of age were included, as were primary studies, including cross-sectional observational, cohort, case-control, quasi-experimental, experimental, and methodological studies. No restrictions regarding language or publication date were applied.

Gray literature (theses, dissertations, institutional documents, and technical reports) was excluded in order to ensure greater reproducibility, methodological standardization, and data comparability, prioritizing studies published in peer-reviewed scientific journals.

### Exclusion criteria

Studies involving participants outside the proposed age range, studies that did not meet the predefined inclusion criteria, studies that did not address specific strategies for the assessment or diagnosis of hearing loss, or studies that did not specify the age at detection were excluded. Secondary studies (systematic, narrative, and integrative reviews, as well as meta-analyses), clinical guidelines, case reports, case series, opinion articles, technical texts, books, websites, blogs, and other non-scientific sources were also excluded. Articles whose full texts could not be obtained, even after contacting the authors, were likewise excluded.

### Search strategy

The bibliographic search was initially conducted in the PubMed/MEDLINE database and subsequently adapted for the following databases: Embase, Scopus, Web of Science, LILACS, Google Scholar, and ProQuest. The search was performed on October 15, 2024, and updated on January 5, 2026 (Appendix A). In Google Scholar, the first 200 references sorted by relevance were screened, in accordance with methodological recommendations for scoping reviews.

The search strategy was adapted to each database and combined controlled and uncontrolled descriptors (MeSH, DeCS, and free keywords) related to the main study concepts, using the Boolean operators AND and OR, as well as truncation and spelling variations, according to the specificities of each database.

### Reference management and screening

The search results were initially exported to Zotero software (version 7.0), which was used exclusively for reference organization and automatic duplicate removal. Subsequently, the records were imported into the Rayyan platform^(23)^ where an additional manual duplicate check was performed to increase the accuracy of the process, followed by title and abstract screening.

Before the selection process began, the reviewers participated in a calibration stage involving the joint analysis of a pilot sample of studies in order to standardize the application of the eligibility criteria.

### Study selection

Study selection was carried out in two stages. In the first stage, titles and abstracts were independently assessed by six reviewers organized into three pairs (pair 1: ADSL and SAS; pair 2: APCF and ROF; pair 3: MCOA and MHM). The division into three pairs aimed to distribute the volume of records evenly, ensuring greater efficiency and rigor during the initial screening process.

Each database was reviewed by at least one pair of reviewers, ensuring the uniform application of the eligibility criteria. In the second stage, the full texts of potentially eligible studies were independently assessed by the same reviewers.

Decisions were initially made by consensus within each pair. In cases of disagreement, the study was discussed in meetings involving all reviewer pairs. If disagreement persisted, a third independent reviewer was consulted for the final decision. Throughout all stages, reviewers remained blinded to the decisions of the other reviewer pairs, according to the functionality provided by the Rayyan platform.

### Data extraction and analysis

Data were extracted using a structured form developed by the authors, including: (a) study identification (authors, year, country, language, and type of publication); (b) methodological characteristics (study design, sample, instruments used, and target population); (c) description of the applied early diagnosis strategies; and (d) main findings and conclusions.

The results were synthesized descriptively and narratively, with presentation in tables and figures grouping the identified strategies according to the type of approach (technological, clinical, educational, or policy-related). Whenever applicable, knowledge gaps and recommendations for future research in the field of child hearing health were highlighted.

## RESULTS

**Figure 1:**
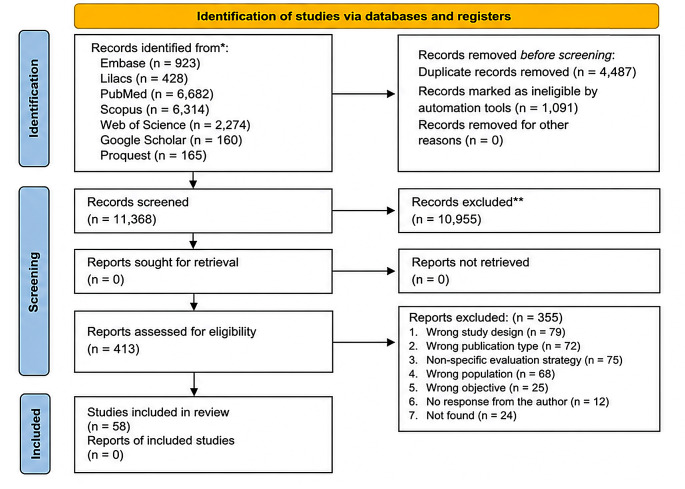
Flow diagram according to the PRISMA-ScR guidelines^(25)^ (adapted)

The study search and selection process is presented in the PRISMA-ScR flowchart (Figure 1). A total of 16,946 records were initially identified across the searched databases. After duplicate removal and title and abstract screening, 413 articles were selected for full-text review, of which 355 were excluded (Appendix B). Consequently, 58 studies met the eligibility criteria and comprised the final sample. Most exclusions were due to the absence of a focus on early diagnosis, inclusion of age groups older than two years, or the use of study designs incompatible with the scope of this review.

*Consider, if feasible to do so, reporting the number of records identified from each database or register searched (rather than the total number across all databases/registers); **If automation tools were used, indicate how many records were excluded by a human and how many were excluded by automation tools

**Figure 2:**
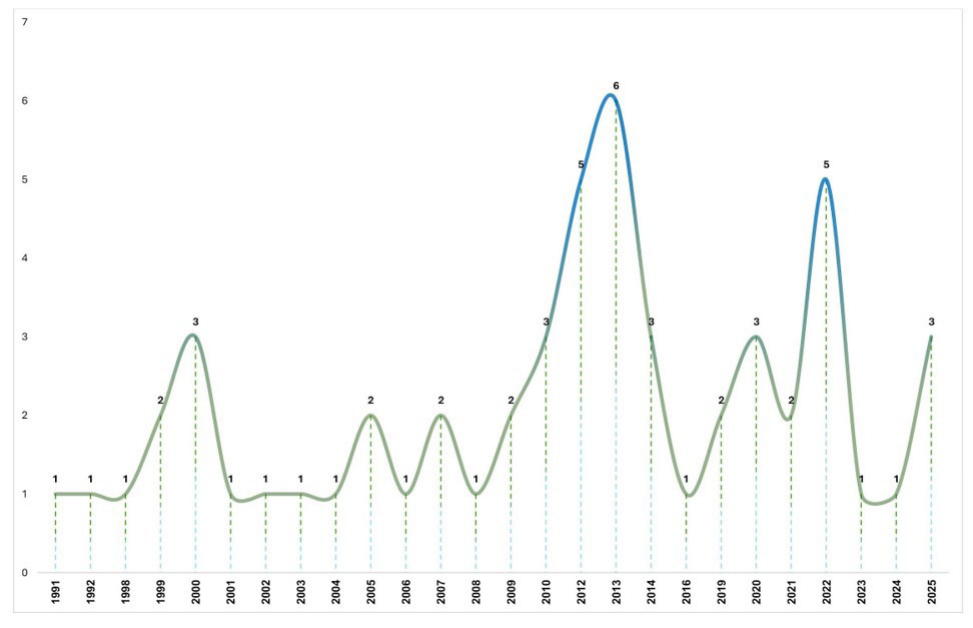
Temporal distribution of studies included in the review

The general characteristics of the included studies are presented in Table 1. Publications ranged from 1991 to 2025, with a marked increase after 2015 (n = 20)^(64-83)^. The temporal distribution of publications is shown in Figure 2.

**Table 1:** Characteristics of the studies included in the review

	**Authors (Year)**	**Methodological Characteristics**		**Procedures**	**Outcomes**
		**Study Design**	**Sample**		
1	Webb and Stevens (1991)^(26)^	Comparative	250	TEOAE / ABR	Newborn hearing screening should begin with TEOAE due to its efficiency and cost-effectiveness. ABR should be used for diagnostic confirmation and differentiation of hearing loss type.
2	Tudehope et al. (1992)^(27)^	Prospective	149	ABR / BOA / Immittance	Early auditory assessment in very low birth weight neonates is essential for identifying hearing loss, particularly conductive loss. The combination of ABR, behavioral audiometry, and immittance measures provides a comprehensive diagnostic approach.
3	Morlet et al. (1998)^(28)^	Prospective	1.531	TEOAE / ABR	The screening process successfully identified hearing loss in a high-risk population, highlighting the need for repeated assessments during the first year of life for children with initially abnormal results.
4	Aidan et al. (1999)^(29)^	Cohort	1.727	TEOAE	The study demonstrated the feasibility and limitations of TEOAE as a universal newborn hearing screening tool.
5	Watkin and Baldwin (1999)^(30)^	Retrospective	100	TEOAE / ABR / BOA	Newborn hearing screening is effective for early identification of hearing impairment; however, adequate follow-up and effective parental communication are essential to confirm diagnosis and initiate early intervention.
6	Prieve et al. (2000)^(31)^	Prospective	43.311	TEOAE / AABR / ABR	Outpatient indicators improved over time, with significant differences between neonates admitted to the NICU and those from routine nurseries, in addition to regional disparities requiring further investigation.
7	Shehata-Dieler et al. (2000)^(32)^	Prospective	1.349	ABR / OAE	The BeRapHon® method proved to be an effective and highly specific tool for universal newborn hearing screening.
8	Stewart et al. (2000)^(33)^	Prospective	11.711	AABR / ABR / Tympanometry / TEOAE	Universal hearing screening using AABR proved highly effective and feasible across different hospitals, with low follow-up costs and minimal interference in parent–infant bonding.
9	Baumann and Schorn (2001)^(34)^	Cohort	102	TEOAE / BOA / ABR	Behavioral band testing was inadequate for hearing screening, detecting only 61.5% of cases. In contrast, otoacoustic emissions testing effectively identified hearing loss, and automated devices such as Echoscreen demonstrated high specificity.
10	Owen et al. (2001)^(35)^	Prospective	683	OAE	Community-based newborn hearing screening conducted by healthcare workers was feasible, well accepted, and associated with high population coverage and low false-positive rates.
11	Lin et al. (2002)^(36)^	Prospective	6.765	TEOAE / ABR	The use of TEOAE as a universal newborn hearing screening tool was feasible, accurate, and cost-effective.
12	Iwasaki et al. (2003)^(37)^	Prospective	4.085	AABR / ABR / OAE / BOA	A two-stage AABR screening protocol effectively reduced referral and false-positive rates among newborns.
13	Rouev et al. (2004)^(38)^	Prospective	1.838	AABR / ABR	Universal newborn hearing screening in Bulgaria using a two-stage protocol with AABR and ABR proved effective and cost-effective for early hearing loss detection, despite challenges related to follow-up of referred cases.
14	White et al. (2005)^(39)^	Prospective	86.634	OAE / AABR	The newborn hearing screening protocol using OAE and AABR identified most infants with permanent hearing loss; however, a substantial proportion would have passed the AABR screening.
15	Widen et al. (2005)^(40)^	Prospective	973	OAE / AABR / VRA	Newborn hearing screening should include continuous monitoring beyond the neonatal period. VRA was appropriate for most infants, although examiner experience influenced diagnostic effectiveness.
16	Yee-Arellano et al. (2006)^(41)^	Prospective	3.066	AABR / ABR	Even with a reduced pilot sample, the rates of identified hearing loss were similar to those reported in other countries. The study highlighted the feasibility of the program and the need for expansion, emphasizing operational and follow-up aspects.
17	De Capua et al. (2007)^(42)^	Prospective	19.700	OAE / ABR / BOA	Diagnostic ABR was preferable to automated ABR due to its greater diagnostic accuracy.
18	Tatli et al. (2007)^(43)^	Prospective	711	TEOAE / ABR	A two-stage TEOAE newborn hearing screening program was feasible and well accepted by families. Effective screening was achieved in 96% of neonates admitted to the nursery.
19	Olusanya et al. (2008)^(44)^	Cross-sectional	1.330	TEOAE / ABR	Hospital-based universal newborn hearing screening was feasible in urban Nigerian settings, even when conducted by non-specialized personnel after brief training, showing effective detection and low referral rates.
20	Dantas et al. (2009)^(45)^	Retrospective	1.626	TEOAE	The findings reinforced the importance of implementing and maintaining newborn hearing screening programs and may support comparisons in regional and multinational studies.
21	Ohl et al. (2009)^(46)^	Retrospective	1.461	Automated OAE / OAE / ABR	The screening program for high-risk infants enabled early management of 60 deaf children, although loss to follow-up after screening remained substantial.
22	Geal-Dor et al. (2010)^(47)^	Comparative	1.545	TEOAE / BOA	Newborn hearing screening using TEOAE was objective and favored early detection and adherence. Subsequent behavioral screening was useful for identifying late-onset or progressive hearing deficits, including auditory neuropathy. Both methods were complementary rather than interchangeable.
23	Tasci et al. (2010)^(48)^	Retrospective	16.975	TEOAE / ABR	The three-stage protocol was accurate and noninvasive; however, reorganization into a two-stage approach (initial TEOAE followed by ABR) was suggested to improve coverage.
24	Yu et al. (2010)^(49)^	Retrospective	6.336	DPOAE / AABR	The combined DPOAE and AABR screening protocol proved feasible and more efficient for newborn hearing screening.
25	Daniel and Lim (2012)^(50)^	Retrospective	100.237	OAE / AABR / ABR	The newborn hearing screening program in Singapore was effective for early identification of hearing impairment and provided opportunities for timely intervention.
26	Friderichs et al. (2012)^(51)^	Cross-sectional	6.227	DPOAE	Low screening coverage highlighted the need for dedicated personnel, rather than regular clinic staff, to achieve broader and more effective screening rates.
27	Lim et al. (2012)^(52)^	Retrospective	10.879	AABR / ABR / Tympanometry	The AABR screening protocol demonstrated acceptable referral rates and predictive values.
28	Martines et al. (2012)^(53)^	Comparative	412	TEOAE / Tympanometry / ABR	A high prevalence of auditory neuropathy spectrum disorder was identified among high-risk infants. Family history and NICU admission were independent predictors. Combined screening (TEOAE + ABR) demonstrated better performance than isolated TEOAE, especially for detecting auditory neuropathy in high-risk neonates.
29	Zhang et al. (2012)^(54)^	Prospective	10.043	DPOAE / AABR / Genetic screening	Combined hearing and genetic screening improved identification of newborns at risk for hearing loss, including cases associated with recessive mutations or drug-induced hearing impairment.
30	Arslan et al. (2013)^(55)^	Prospective	2.229	Automated TEOAE / AABR	Automated TEOAE proved feasible for newborn hearing screening, and the two-stage protocol effectively reduced false-positive referral rates.
31	Borkoski Barreiro et al. (2013)^(56)^	Retrospective	26.717	Automated TEOAE / TEOAE / ABR / ASSR	The early hearing detection program achieved coverage above 95%, although referral rates for diagnostic confirmation remained below recommended levels. Nevertheless, the protocol proved suitable for hospital routine and ensured substantial screening coverage.
32	Hsu et al. (2013)^(57)^	Prospective	3.361	TEOAE / AABR	The universal newborn hearing screening program using AABR demonstrated high coverage and low referral rates.
33	Kuki et al. (2013)^(58)^	Comparative	50	BOA / TEOAE / AABR / ABR / ASSR	Combining BOA with TEOAE and/or AABR enabled accurate, cost-effective, and well-accepted hearing screening. Due to its simplicity, low cost, and acceptability, BOA may be especially useful in low-resource settings where universal screening is not yet implemented.
34	Mishra et al. (2013)^(59)^	Prospective	1.101	DPOAE / ABR	The combination of DPOAE and ABR was effective for universal newborn hearing screening and early detection of hearing loss in children younger than 2 years.
35	Qi et al. (2013)^(60)^	Prospective	10.983	TEOAE / ABR	High screening coverage and reduced referral rates were feasible and practical among newborns of migrant workers.
36	Amini et al. (2014)^(61)^	Case series	149	TEOAE / Tympanometry / ABR	A significant association was observed between low birth weight and abnormal otoacoustic emissions. Altered OAE responses were identified in 2% of asphyxiated newborns.
37	Çelik et al. (2014)^(62)^	Retrospective	142.128	TEOAE / ABR	Hearing screening proved essential for all newborns to support developmental outcomes. Infants with risk factors required repeated testing for early diagnosis, and improvements in prenatal care could contribute to reducing hearing impairment rates.
38	Huang et al. (2014)^(63)^	Prospective	13.315	TEOAE / AABR / ABR	The combination of TEOAE and AABR followed by complete audiological assessment constituted an effective universal newborn hearing screening model for high-risk infants admitted to the NICU.
39	Canet et al. (2016)^(64)^	Retrospective	14.247	OAE / ABR	The screening program proved useful; however, improvements in tracking and follow-up processes were necessary. Effectiveness was limited by loss to follow-up, reinforcing the importance of electronic records and coordinated care.
40	Chrobok et al. (2019)^(65)^	Descriptive	—	OAE / ABR	Newborn hearing screening techniques (TEOAE and ABR) were effective, objective, and rapid. Universal implementation was considered essential for early identification of hearing loss and intervention before six months of age.
41	Wasser et al. (2019)^(66)^	Prospective	179.000	TEOAE / ABR	The national newborn hearing screening program in Israel achieved high coverage and significantly reduced the age at diagnosis and habilitation among infants with hearing loss.
42	Dey et al. (2020)^(67)^	Prospective	264	DPOAE / ABR	Hearing impairment was associated with hyperbilirubinemia requiring phototherapy or exchange transfusion, suggesting its role as a predictive indicator for hearing loss development.
43	McInerney et al. (2020)^(68)^	Retrospective	604	DPOAE / ABR	The findings highlighted the need to address barriers associated with loss to follow-up and recommended the use of strategies to improve parental engagement.
44	Nishad et al. (2020)^(69)^	Prospective	1.000	TEOAE / ABR	A well-structured protocol including initial OAE screening and ABR retesting was necessary for early hearing loss detection. Higher incidence rates were observed among high-risk neonates. OAE was useful for rapid screening, whereas ABR remained essential for definitive diagnosis.
45	Kelly et al. (2021)^(70)^	Retrospective	31.984	ABR	Delaying ABR testing to approximately 10–11 hours after vaginal delivery and 30–32 hours after cesarean delivery significantly improved pass rates. Inclusion of a third post-discharge test further reduced referral rates.
46	Shim et al. (2021)^(71)^	Retrospective	3.059	AABR / ABR / Tympanometry / ASSR / TEOAE	The two-stage AABR screening protocol demonstrated effective performance within JCIH-recommended referral rates (3–4%). The study validated this method as appropriate for hospital-based newborn hearing screening.
47	Arora et al. (2022)^(72)^	Prospective	1.200	TEOAE / ABR / ASSR	Newborn hearing screening was feasible in low-resource settings, particularly when associated with community programs and parental education strategies.
48	Gharibi and Khavidaki (2022)^(73)^	Cross-sectional	7.700	TEOAE / AABR / ABR	A comprehensive newborn hearing screening program was essential to ensure timely diagnosis and treatment, with early identification and intervention being decisive for effective auditory rehabilitation.
49	Hajare and Mudhol (2022)^(74)^	Comparative	800	DPOAE / AABR / ABR	A centralized screening facility could benefit hospitals lacking resources for universal or high-risk hearing screening programs.
50	Khaimook et al. (2022)^(75)^	Retrospective	1.579	OAE / ABR / Tympanometry	The universal newborn hearing screening program was effective in early identification and referral of children with hearing loss, highlighting family support and interdisciplinary care as key factors for improved outcomes.
51	Mazlan et al. (2022)^(76)^	Retrospective	50.569	TEOAE / AABR / ABR	The program achieved excellent screening coverage; however, performance remained below expectations regarding referral, follow-up, and diagnostic confirmation. Program revision and optimization were recommended to maintain effectiveness and relevance.
52	Dutra et al. (2023)^(77)^	Cross-sectional	104	TEOAE / VRA / BOA / ABR / ASSR	Limited access to hearing healthcare services delayed diagnostic confirmation and intervention. Maternal age, education, and distance from healthcare services did not significantly influence service utilization.
53	Badusha et al. (2024)^(78)^	Prospective	117	DPOAE / ABR	The high incidence of hearing impairment among high-risk infants reinforced the importance of newborn hearing screening. Collaboration between healthcare professionals and parents was essential for effective screening and intervention.
54	Kgare and Joubert (2024)^(79)^	Retrospective	2.302	Tympanometry / ABR / ASSR	Community-based universal newborn hearing screening proved feasible in a rural setting; however, high loss-to-follow-up rates and delayed screening age compromised the effectiveness of early diagnosis.
55	Jennings and Isaacson (2025)^(80)^	Retrospective	2.069	TEOAE / AABR	Although newborn hearing screening before hospital discharge achieved high coverage, early diagnostic effectiveness was compromised by outpatient loss to follow-up (43%) and socioeconomic barriers such as transportation, insurance, and childcare responsibilities.
56	Chandrakapure et al. (2025)^(81)^	Retrospective	28.025	BOA / TEOAE / AABR / ABR	Universal newborn hearing screening conducted at a tertiary healthcare center achieved high coverage and excellent diagnostic performance; however, poor adherence to post-screening follow-up limited the effectiveness of early diagnosis.
57	Ndoleriire et al. (2025)^(82)^	Cross-sectional	12.996	TEOAE / AABR / ABR / Tympanometry	Quality-controlled newborn hearing screening proved feasible in a public hospital setting; however, large variations in coverage and high loss-to-follow-up rates compromised the effectiveness of early hearing loss diagnosis.
58	Parangrit et al. (2025)^(83)^	Retrospective	6.472	OAE / AABR	The implementation of an earlier and more structured newborn hearing screening protocol increased screening coverage and timely diagnosis, although loss to follow-up remained a major limitation in the care pathway.

**Caption:** BOA = Behavioral Observation Audiometry; AABR = Automated Auditory Brainstem Response; ABR = Auditory Brainstem Response; ASSR = Auditory Steady-State Response; DPOAE = Distortion Product Otoacoustic Emissions; Immittance = Immittance Measures; TEOAE = Transient Evoked Otoacoustic Emissions; Tympanometry = Tympanometry; VRA = Visual Reinforcement Audiometry

Source: Page et al.^(24)^

**Figure 3:**
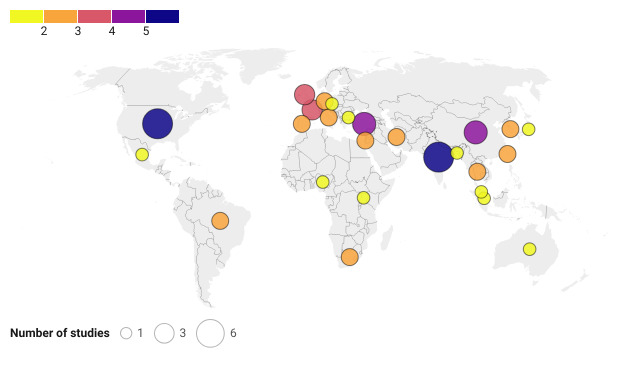
Geographic distribution of studies included in the review. Created with Datawrapper

Regarding geographic distribution, studies conducted in North America, Europe, and Asia predominated, particularly those from the United States^(31,33,39,40,68,70,80)^, India^(58,59,69,72,74,78,81)^, China^(49,54,60,63)^, and Türkiye^(43,46,55,62)^. Figure 3 presents the geographic distribution of the included studies. Few publications originated from Latin America (n = 3)^(41,45,77)^ and Africa (n = 4)^(44,51,79,82)^.

Descriptive observational studies, mainly cross-sectional and retrospective in design, predominated and accounted for most of the sample (n = 35). These studies were primarily linked to Universal Newborn Hearing Screening (UNHS) programs or referral hospital services. Prospective cohort studies and clinical trials were less frequent (n = 18) and mainly aimed at evaluating diagnostic accuracy and validating combined protocols. Sample sizes varied considerably, ranging from 50^(58)^ to 179,000^(66)^ participants, demonstrating substantial heterogeneity among the included studies.

The identified strategies for the early diagnosis of childhood hearing loss predominantly involved universal newborn hearing screening, with the use of otoacoustic emissions (TEOAE/DPOAE) reported in 89.6% of the studies (n = 52), auditory brainstem response (ABR) in 77.5% (n = 45), and automated ABR (AABR) in 41.3% (n = 24). Two-stage protocols, generally consisting of OAE followed by AABR, were described in approximately 35% of the studies (n = 20).

Complementary strategies were also identified, including approaches directed toward at-risk populations (n = 7)^(26,28,46,53,68,69,74,78)^, parental education and counseling interventions (n = 2)^(68,72)^, expanded electrophysiological test batteries (n = 7)^(38,41,56,58,70-72)^, and behavioral assessments (n = 9)^(27,30,34,37,42,47,58,77,81)^. To a lesser extent, some studies reported the incorporation of complementary etiological testing, such as genetic screening associated with neonatal hearing assessment (n = 1)^(54)^.

Regarding healthcare settings, most studies were conducted in maternity wards and public hospitals (n = 52), whereas initiatives developed in primary healthcare or community services were less frequent (n = 6)^(35,47,51,53,65,79)^. The main reported barriers included loss to follow-up during retesting, communication failures between maternity hospitals and specialized services, lack of equipment availability, and logistical difficulties related to access to healthcare services^(31,34,47,52,60,66,77)^. In contrast, studies reporting better healthcare indicators described the adoption of universal two-stage protocols, active family follow-up strategies, computerized case-monitoring systems, and structured parental education programs^(39,43,48,55,56,58,61,67,72,73,82)^.

**Figure 4:**
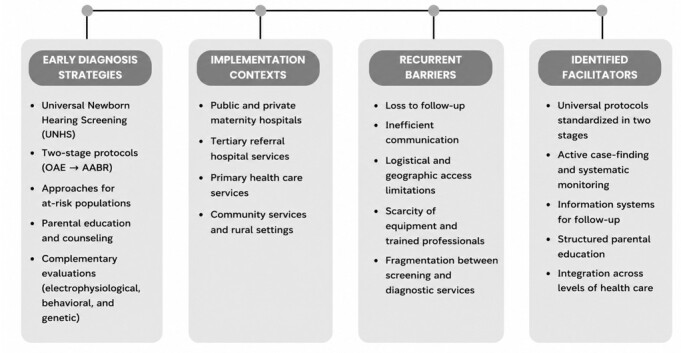
Synthesis of strategies and barriers related to the early diagnosis of hearing loss in infants

Based on the synthesis of the included studies, a summary figure (Figure 4) was developed integrating diagnostic strategies, healthcare settings, and recurrent barriers related to the early diagnosis of childhood hearing loss.

## DISCUSSION

The temporal and geographic distribution of the included studies reveals important patterns in the scientific literature on the early diagnosis of childhood hearing loss. An increase in publications was observed after 2015, along with a concentration of studies conducted in high-income countries, particularly in the United States, Europe, and Asia, highlighting inequalities in both scientific production and the implementation of structured screening programs.

This scoping review demonstrated substantial variability in healthcare pathways and in the standardization of protocols aimed at the early detection and intervention of childhood hearing loss^(26-78)^. Such heterogeneity reflects not only methodological differences among studies, but also structural inequalities across healthcare systems, related to the organization of healthcare networks, resource availability, and integration between levels of care. At the same time, the diversity of the analyzed contexts enabled the identification of successful strategies that contribute to the timely diagnosis of hearing impairment, even in settings with logistical constraints^(9,26,29,36)^.

Across the included studies, the combination of otoacoustic emissions and auditory brainstem response remained the cornerstone of early detection strategies, associating greater diagnostic sensitivity with earlier initiation of hearing rehabilitation^(27,34,44,72,76)^. The historical evolution of Universal Newborn Hearing Screening (UNHS), initially targeted at high-risk neonates and later expanded to the entire neonatal population, demonstrated a consistent impact on reducing the mean age at diagnosis and improving access to hearing interventions, regardless of the healthcare setting^(26-29,38,60,76,84)^.

Despite its widespread adoption, the findings of this review indicate that the effectiveness of UNHS is strongly influenced by the way protocols are operationalized. The use of two-stage protocols, involving the sequential application of OAE and automated ABR, proved effective in reducing false-positive results and improving diagnostic specificity^(81,85-87)^. Furthermore, retesting performed before hospital discharge or within the first 48–72 hours of life was associated with higher adherence rates and fewer unnecessary referrals, minimizing transient interferences related to the neonatal period^(8,33,37,41,52,71,80,87)^. These findings reinforce that the quality of healthcare pathways is as important as the choice of the diagnostic method itself.

Programmatic and management-related aspects emerged as critical determinants of screening program performance. Coordination by specialized professionals, exemption from costs in subsequent stages, and integration with regional and national healthcare networks were consistently associated with reduced loss to follow-up^(8,78,82)^. These results support the interpretation that the effectiveness of UNHS depends less on isolated technology and more on service organization and continuity of care.

Community-based screening models, particularly those incorporating community health workers, demonstrated potential to expand coverage and reduce dropout rates, especially in rural or hard-to-reach areas^(35,44)^. Such experiences suggest that territorially based strategies may mitigate logistical and geographic barriers, contributing to greater equity in access to hearing healthcare services^(35,44,79)^.

Another relevant finding concerns the limitations of hearing screening as a single event. Included studies demonstrated that infants who “pass” the initial screening may later develop late-onset hearing loss, resulting in delayed diagnosis and intervention^(40,66,68,88)^. This evidence reinforces the need for continuous clinical and etiological monitoring, with periodic recall for complementary examinations, such as diagnostic ABR and behavioral audiometry, throughout the first years of life.

Among the complementary strategies, genetic screening emerged as a promising approach, particularly for the early identification of hearing loss not detected by conventional auditory methods and for the prevention of conditions associated with ototoxicity^(54,89-91)^. Similarly, the combined use of electrophysiological tests, such as Auditory Steady-State Response, contributed to estimating hearing loss severity and audiometric configuration, although controversies remain regarding its large-scale feasibility^(26,28,40,50,66,92-96)^.

Taken together, the findings of this review indicate that the effectiveness of early diagnosis programs for childhood hearing loss results from the articulation between technological strategies, service organization, and integration across different levels of healthcare. Fragmentation between screening, diagnosis, and intervention remains one of the main barriers to achieving the internationally recommended continuum of care^(12,88)^.

This review has limitations inherent to its design, including the methodological heterogeneity of the included studies and the predominance of research conducted in high-income countries, which may limit the generalizability of the findings. Nevertheless, by integrating strategies, contexts, and barriers into a single analytical synthesis, this study contributes to a broader understanding of the strengths and weaknesses of early diagnosis programs for childhood hearing loss, highlighting pathways for improving public policies and healthcare practices.

## CONCLUSION

This scoping review mapped the main strategies for the early diagnosis of hearing loss in infants, highlighting the predominance of Universal Newborn Hearing Screening based on combined protocols. Although diagnostic methods are well established, considerable variability was observed in protocol operationalization across different healthcare settings, indicating that the effectiveness of early diagnosis depends more on the organization of healthcare pathways and the integration between screening, diagnosis, and follow-up than on isolated technology. The scarcity of studies originating from socially vulnerable settings reveals important gaps in the literature and highlights the need for future investigations addressing different healthcare realities.

## Appendix A Search strategy across databases

**Table:**

Database	Search (October 15^th^ 2024, updated January 5, 2026)
Embase	#1. 'newborn'/exp OR 'animals, newborn' OR 'child, newborn' OR 'full term infant' OR 'human neonate' OR 'human newborn' OR 'infant, newborn' OR 'neonatal animal' OR 'neonate' OR 'neonate animal' OR 'neonatus' OR 'newborn animal' OR 'newborn animals' OR 'newborn baby' OR 'newborn child' OR 'newborn infant' OR 'newly born animal' OR 'newly born baby' OR 'newly born child' OR 'newly born infant' OR 'newborn'
	#2. 'hearing impairment'/exp OR 'auditory defect' OR 'deaf' OR 'deafness' OR 'hard of hearing' OR 'hearing damage' OR 'hearing defect' OR 'hearing difficulty' OR 'hearing loss' OR 'hypacousia' OR 'hypacousis' OR 'hypacusia' OR 'hypacusis' OR 'hypakousia' OR 'hypakusis' OR 'hypoacousia' OR 'hypoacousis' OR 'hypoacusia' OR 'hypoacusis' OR 'hypoakusis' OR 'impaired hearing' OR 'hearing impairment'
	#3. 'early diagnosis'/exp OR 'diagnosis, early' OR 'early diagnosis' OR 'newborn screening'/exp OR 'mass screening, newborn' OR 'neonatal screening' OR 'screening, newborn' OR 'newborn screening'
	#4. #1 AND #2 AND #3
	#5. #4 AND [embase]/lim NOT ([embase]/lim AND [medline]/lim)
LILACS	(mh:"Recém-Nascido" OR "Recém-Nascido" OR "Criança Recém-Nascida" OR "Crianças Recém-Nascidas" OR "Lactente Recém-Nascido" OR "Lactentes Recém-Nascidos" OR neonato neonatos "Recém-Nascido (RN)" OR "Recém-Nascidos" OR "Newborn Infant" OR "Recién Nacido" OR mh:m01.060.703.520*) AND (mh:"Perda Auditiva" OR "Perda Auditiva" OR "Deficiência Auditiva" OR hipoacusia OR "Perda Auditiva Transitória" OR "Perda da Audição" OR "Perda da Capacidade Auditiva" OR "Surdez Transitória" OR "Hearing Loss" OR "Pérdida Auditiva" OR mh:c09.218.458.341* OR mh:c10.597.751.418.341* OR mh:c23.888.592.763.393.341*) AND (mh:"Diagnóstico Precoce" OR "Diagnóstico Precoce" OR "Early Diagnosis" OR "Diagnóstico Precoz" OR mh:e01.390* OR mh:"Triagem Neonatal" OR "Triagem Neonatal" OR "Rastreamento Neonatal" OR "Rede Estadual" de "Triagem Neonatal" OR "Triagem Neonatal Universal" OR "Triagem do Recém-Nascido" OR "Neonatal Screening" OR "Tamizaje Neonatal" OR mh:e01.370.225.910* OR mh:e01.370.500.580* OR mh:e05.200.910* OR mh:e05.318.308.980.438.580.580* OR mh:n02.421.726.233.443.816* OR mh:n05.715.360.300.800.438.500.575* OR mh:n06.850.520.308.980.438.580.580* OR mh:n06.850.780.500.580*)
PubMed/ Medline	#1. "Infant"[Mesh] OR Infants OR "Infant, Newborn"[Mesh] OR "Newborn Infant" OR "Newborn Infants" OR Neonate OR Neonates OR Newborns OR Newborn
	#2. "Hearing Loss"[Mesh] OR "Hearing Impairment"
	#3. "Early Diagnosis"[Mesh] OR "Early Detection of Disease" OR "Disease Early Detection" OR "Mass Screening"[Mesh] OR "Mass Screenings" OR Screening OR Screenings OR "Neonatal Screening"[Mesh] OR "Neonatal Screenings" OR "Newborn Screening" OR "Newborn Screenings" OR "Newborn Infant Screening"
	#4. #1 AND #2 AND #3
Scopus	(TITLE-ABS-KEY ( infant OR infants OR "Newborn Infant" OR "Newborn Infants" OR neonate OR neonates OR newborns OR newborn) AND TITLE-ABS-KEY ("Hearing Loss" OR "Hearing Impairment") AND TITLE-ABS-KEY ("Early Diagnosis" OR "Early Detection of Disease" OR "Disease Early Detection" OR "Mass Screening" OR "Mass Screenings" OR screening OR screenings OR "Neonatal Screening" OR "Neonatal Screenings" OR "Newborn Screening" OR "Newborn Screenings" OR "Newborn Infant Screening" ))
Web of Science	Infant OR Infants OR "Newborn Infant" OR "Newborn Infants" OR Neonate OR Neonates OR Newborns OR Newborn (Topic) AND "Hearing Loss" OR "Hearing Impairment" (Topic) AND "Early Diagnosis" OR "Early Detection of Disease" OR "Disease Early Detection" OR "Mass Screening" OR "Mass Screenings" OR Screening OR Screenings OR "Neonatal Screening" OR "Neonatal Screenings" OR "Newborn Screening" OR "Newborn Screenings" OR "Newborn Infant Screening" (Topic)
Google Scholar	"Newborn Infant" AND "Hearing Loss" AND "Early Diagnosis" OR "Neonatal Screening"
*ProQuest*	Infant OR Infants OR "Newborn Infant" OR "Newborn Infants" OR Neonate OR Neonates OR Newborns OR Newborn (Topic) AND "Hearing Loss" OR "Hearing Impairment" (Topic) AND "Early Diagnosis" OR "Early Detection of Disease" OR "Disease Early Detection" OR "Mass Screening" OR "Mass Screenings" OR Screening OR Screenings OR "Neonatal Screening" OR "Neonatal Screenings" OR "Newborn Screening" OR "Newborn Screenings" OR "Newborn Infant Screening" (Topic)

## Appendix B Reasons for exclusion of studies *(n = 355)*

**Table:**

Nº	Author, Year	Reason for exclusion*
[1]	Pérez et al., 2009	4
[2]	Abdul Hadi et al., 2012	1
[3]	Abdulai et al., 2020	2
[4]	Abdullah et al., 2006	1
[5]	Adelola et al., 2010	5
[6]	Ahmed et al., 2018	1
[7]	Aiyer; Parikh, 2009	1
[8]	Alberti et al., 1983	4
[9]	Al-Kandari; Alshuaib, 2007	5
[10]	Alarcón Avila et al., 2025	2
[11]	Allen; Lambert, 1990	4
[12]	Almenar Latorre et al., 2002	5
[13]	Alves De Sousa et al., 1996	2
[14]	Anderssen et al., 2002	3
[15]	Angrisani et al., 2012	2
[16]	Ansari, 2022	2
[17]	Applebaum, 1999	2
[18]	Aras Öztürk et al., 2018	3
[19]	Ardic, 2017	4
[20]	Arnold et al., 2006	5
[21]	Arslan et al., 2013a	2
[22]	Arslan et al., 2013b	2
[23]	Ataoğlu et al., 2019	1
[24]	Azevedo, 1991	7
[25]	Azevedo et al., 2004	7
[26]	Azizi et al., 2016	1
[27]	Babac; Djerić; Ivanković, 2007	5
[28]	Back; Ho, 2011	3
[29]	Backous, 2002	2
[30]	Bagatto et al., 2020	1
[31]	Bai et al., 2024	1
[32]	Bansal; Gupta; Nagarkar, 2008	3
[33]	BarskyFirkser; Sun, 1997	1
[34]	Bartmeyer; Navarini; Carvalho, 2025	3
[35]	Begum et al., 2024	3
[36]	Bellia et al., 2020	1
[37]	Benito Orejas et al., 2008a	3
[38]	Benito Orejas et al., 2008b	3
[39]	Benito Orejas; Silva Rico, 2017	2
[40]	Berger et al., 2012	4
[41]	Bertoldi; Manfredi; Mitre, 2017	4
[42]	Bevilacqua et al., 2010	3
[43]	Bhalot et al., 2023	3
[44]	Bhatia et al., 2013	4
[45]	Biaggio et al., 2015	2
[46]	Bianchin et al., 2022	4
[47]	Biscegli et al., 2015	3
[48]	Bishnoi et al., 2019	2
[49]	Bonelli et al., 2000	4
[50]	Botasso; Lima; Correa, 2022a	4
[51]	Botasso; Lima; Correa, 2022b	4
[52]	Botelho et al., 2010	3
[53]	Bradford et al., 1985	2
[54]	Bradley; Barbera, 2001	2
[55]	Bravo A et al., 2017	5
[56]	Brockow et al., 2011a	3
[57]	Brockow et al., 2011b	3
[58]	Bubbico et al., 2008	1
[59]	Bubbico; Tognola; Grandori, 2017	3
[60]	Buser et al., 2003	5
[61]	Calcutt et al., 2016	5
[62]	Calevo et al., 2007	3
[63]	Campos Banãles et al., 2003	4
[64]	Campos et al., 2003	4
[65]	Canale et al., 2006	4
[66]	Cao-Nguyen; Kos; Guyot, 2007	4
[67]	Cardoso et al., 2009	4
[68]	Cassidy; Ditty, 2001	1
[69]	Ceccato et al., 1996	2
[70]	Černý et al., 2003	3
[71]	Chadha; Bais, 1997	4
[72]	Champion, 2021	4
[73]	Chang et al., 2012	1
[74]	Chen et al., 2012	4
[75]	Chen et al., 2017	4
[76]	Chibisova et al., 2014	3
[77]	Chiriboga et al., 2021	2
[78]	Choi et al., 2022	4
[79]	Chu et al., 2015	1
[80]	Chung; Oh; Park, 2020a	6
[81]	Chung; Oh; Park, 2020b	6
[82]	Cianfrone et al., 2018	4
[83]	Cianfrone et al., 2021	1
[84]	Colella-Santos et al., 2013a	4
[85]	Colella-Santos et al., 2013b	1
[86]	Coll; Régnier, 1988	6
[87]	Collins et al., 2022	7
[88]	Costa et al., 2015	3
[89]	Cox; Toro, 2001	1
[90]	Cundall, 1997	7
[91]	Curcin; Sitka; Rasinski, 1998	6
[92]	Curnock, 1993	2
[93]	Da Silva et al., 2017	2
[94]	Dalzell et al., 2000	3
[95]	Dantas et al., 2009	3
[96]	Davis; Hind, 2003	1
[97]	Daykhes et al., 2017	3
[98]	De Barros Boishardy et al., 2005	3
[99]	De González Dios; Mollar Maseres, 2005	3
[100]	De Leenheer et al., 2011	1
[101]	De Mattos et al., 2009	2
[102]	Delgado Domínguez, 2011	2
[103]	Deng et al., 2022	3
[104]	Desloovere et al., 2000	6
[105]	Dlouhá et al., 2002	7
[106]	Downs, 1995	1
[107]	Duci A et al., 2000	2
[108]	Durante et al., 2005	4
[109]	Durante et al., 2004	7
[110]	Eibenstein et al., 2014	2
[111]	Eiserman et al., 2008	4
[112]	Elpers et al., 2016	1
[113]	Farhat et al., 2014	4
[114]	Feroz, et al., 2025	3
[115]	Felden et al., 2017	7
[116]	Fernandes; Nozawa, 2010	1
[117]	Fernandez Martinez et al., 1995	1
[118]	Ferro et al., 2007	4
[119]	Findlen; Malhotra; Hunter,	1
[120]	Fink et al., 2022	1
[121]	Fitzpatrick; Durieux-Smith; Whittingham, 2010	4
[122]	François et al., 2011	1
[123]	Fujikawa; Yoshinaga-Itano, 2000	2
[124]	Fukushima et al., 2008	1
[125]	Gáborján et al., 2019	3
[126]	Gaborján et al., 2022	3
[127]	Galhotra; Sahu, 2019	3
[128]	Garbaruk et al., 2021	2
[129]	Gavilan Cellie, 1993	2
[130]	Geal-Dor et al., 2002	3
[131]	Gellrich et al., 2024	5
[132]	Gentiletti-Faenze et al., 2003	2
[133]	Georgescu; Vladareanu, 2015	4
[134]	Giordano; Marchegiani; Germiller, 2015	7
[135]	Godoy B; Bustamante M, 2006	1
[136]	González De Aledo Linos et al., 2005	3
[137]	González de Dios; Mollar Maseres; Rebagliato Russo, 2005	1
[138]	González Díaz et al., 2020	5
[139]	González; Díaz; Mora, 2022	5
[140]	González; Fernández de Soto; Torres, 2012	2
[141]	Gordo et al., 1994	3
[142]	Granell; Martin, 2011	2
[143]	Greczka et al., 2015	3
[144]	Greczka et al., 2018	1
[145]	Grégoire, 1996	3
[146]	Greville; Keith, 1978	3
[147]	Groh et al., 1999	3
[148]	Guastini et al., 2010	1
[149]	Guilhoto; Quintal; Da Costa, 2003	4
[150]	Guimarães; Barbosa, 2012	1
[151]	Gulati et al., 2022	1
[152]	Halder et al., 2025	6
[153]	Hall; Brosch; Hoffmann, 2020	1
[154]	Hanschmann; Berger, 2010	7
[155]	Harrison et al., 2000	2
[156]	Hatzopoulos et al., 2001	5
[157]	Hayes, 2002	2
[158]	Helge et al., 2005	3
[159]	Hergils, 2000	2
[160]	Hoffmann et al., 2013	1
[161]	Hosforddunn et al., 1987	5
[162]	Huang et al., 2014	4
[163]	Huang et al., 2023	2
[164]	Huang et al., 2009	3
[165]	Huang et al., 2017	4
[166]	Huarte Irujo, 2003	1
[167]	Irfan et al., 2024	2
[168]	Iwanicka-Pronicka et al., 2008	4
[169]	Izumets; Nezhivenko; Murashko, 2015	2
[170]	Jacob et al., 2022	4
[171]	Jacobson; Jacobson, 1990	1
[172]	Jacobson, 2001	6
[173]	Jakubikova et al., 1999	7
[174]	Jakubíková; Kabátová; Závodná, 2003	1
[175]	Januário et al., 2015	1
[176]	Jayne et al., 2023	3
[177]	Johnson et al., 2005	1
[178]	Kanji; Khoza-Shangase, 2012	4
[179]	Kanji; Krabbenhoft, 2018	4
[180]	Kawata et al., 2011	1
[181]	Keefe et al., 2000	2
[182]	Kemper, 2011	4
[183]	Kerschner et al., 2004	3
[184]	Kiese-Himmel; Schroff; Kruse, 1997	1
[185]	Kim et al., 2011	7
[186]	Koester; Nekahm-Heis; Zorowka, 2002	7
[187]	Konukseven et al., 2010	4
[188]	Korres et al., 2006	5
[189]	Korres et al., 2008	2
[190]	Krauss M et al., 2013	1
[191]	Krishnan, 2009	3
[192]	Kumar; Gupta; Sinha, 2017	3
[193]	Lan et al., 2024	1
[194]	Langagne et al., 2008	6
[195]	Lee-Ramos et al., 2023	1
[196]	Leonhardt; Müller, 2008	3
[197]	Lewis; Racai; Bevilacqua, 1987	2
[198]	Lewis et al., 2010	2
[199]	Lim; Daniel, 2008	1
[200]	Lima; Marba; Santos, 2005	4
[201]	Lima et al., 2010	4
[202]	Lima et al., 2015	4
[203]	Ling et al., 2015	7
[204]	Liu et al., 2014	4
[205]	Liu et al., 2001	7
[206]	Lopes et al., 2020	4
[207]	Lotfi; Movallali, 2007;	4
[208]	Low et al., 2005	3
[209]	Lowell, 1960	1
[210]	Magnani et al., 2015	1
[211]	Magnusson; Persson; Sundelin, 2001;	4
[212]	Mahmood et al., 2020	3
[213]	Maluleke; Khoza-Shangase; Kanji, 2019	1
[214]	Maniuc; Chiaburu-Chiosa, 2018	4
[215]	Marco Algarra; Pitarch Ribes; Morant Ventura, 1996	5
[216]	Marinho et al., 2020	1
[217]	Marn, 2000	1
[218]	Marn, 2012	6
[219]	Martines et al., 2007	2
[220]	Martines et al., 2014	1
[221]	Martínez et al., 2003a	4
[222]	Martínez et al., 2003b	4
[223]	Martínez-Pacheco; Ferrán de la Cierva; García-Purriños, 2016	4
[224]	Mason; Herrmann, 1998	3
[225]	Mathur; Dhawan, 2007	4
[226]	Matschke; Plath, 1985a	7
[227]	Matschke; Plath, 1985b	2
[228]	Mattos et al., 2009	2
[229]	Mauk et al., 1991	4
[230]	Maxon et al., 1993	5
[231]	Meier et al., 2004	2
[232]	Mencher, 1974	1
[233]	Meyer et al., 2012	5
[234]	Mijares Nodarse et al., 2006	2
[235]	Molini et al., 1996	2
[236]	Molteni, 2006	7
[237]	Monteiro, 2004	2
[238]	Montovani; Fioravanti; Tamashiro, 1996	2
[239]	Moradi-Joo et al., 2024	3
[240]	Morgan; Canalis, 1991	2
[241]	Mukkun, 2021	3
[242]	Murray; Javel; Watson, 1985	2
[243]	Navarro Rivero et al., 1999	4
[244]	Nennstiel-Ratzel et al., 2007	3
[245]	Newton et al., 1999	1
[246]	Ng; Yun, 1992	2
[247]	Ng et al., 2004	1
[248]	Norton et al., 2000a	1
[249]	Norton et al., 2000b	2
[250]	Obeidat et al., 2024	1
[251]	Olarte et al., 2019	4
[252]	Olusanya, 2001	4
[253]	Olusanya; Ebuehi; Somefun, 2009	1
[254]	Onoda; Azevedo; Santos, 2011	3
[255]	Paat-Ahi; Sikkut; Laarmann, 2014	5
[256]	Pastorino et al., 2005	3
[257]	Paul, 2003	2
[258]	Pedersen et al., 2008	5
[259]	Pelosi et al., 1998	1
[260]	Pitathawatchai; Khaimook; Kirtsreesakul, 2019	3
[261]	Pitathawatchai; Wanachottrakul; Prayuenyong, 2025	6
[262]	Plinkert et al., 1990	1
[263]	Plinkert; Delb, 2001a	1
[264]	Plinkert; Delb, 2001b	3
[265]	Poblano et al., 2000	1
[266]	Polski et al., 2014	3
[267]	Poncet; Chaperon, 1983	4
[268]	Prieve et al., 2013	3
[269]	Prieve; Stevens, 2000	2
[270]	Prince et al., 2003	1
[271]	Ptok, 2004	2
[272]	Radü, 1985	4
[273]	Raghuwanshi et al., 2019	3
[274]	Appanaitis; Rahimian, 2018	5
[275]	Ren et al., 2025	3
[276]	Reis et al., 2019	1
[277]	Richard et al., 2025	6
[278]	Rodrigues, 2020	2
[279]	Roman et al., 2001	4
[280]	Ruiz De La Cuesta; Juste Ruiz; Cortés Castell, 2018	3
[281]	Saitoh et al., 2002	3
[282]	Saki et al., 2017	5
[283]	Santos-Ceballos et al., 2020	7
[284]	Sarno; Clemis, 1980	2
[285]	Sassada et al., 2005	7
[286]	Sass-Lehrer, 2004	2
[287]	Satish; Anil Kumar; Viswanatha, 2019	3
[288]	Schade, 2008	2
[289]	Schauseil-Zipf; Von Wedel, 1988	5
[290]	Schmidt et al., 2007	3
[291]	Schönweiler et al., 2002	4
[292]	Sedano M; San Martín U; Rahal E, 2018	3
[293]	Seguya et al., 2021	4
[294]	Sente; Aleksov-Hatvan, 2006	2
[295]	Shang et al., 2016	4
[296]	Sharma et al., 2022	4
[297]	Shehata-Dieler et al., 2000	3
[298]	Shi et al., 2025	3
[299]	Shirane et al., 2020	4
[300]	Shulman et al., 2010	1
[301]	Solanellas Soler, 2009	2
[302]	Sun et al., 2003	7
[303]	Sun et al., 2009	4
[304]	Sun et al., 2015	4
[305]	Sun et al., 2019	7
[306]	Swanepoel; Delport; Swart, 2004	3
[307]	Szyfter et al., 2008	2
[308]	Szyfter et al., 2016	1
[309]	Tanon-Anoh; Sanogo-Gone; Kouassi, 2010	5
[310]	Tham; Lee; Joseph, 2010	1
[311]	Todd, 2006	3
[312]	Trinidad Ruiz et al., 2005	4
[313]	Tufatulin et al., 2023	2
[314]	Turan; Bas, 2019	1
[315]	Tzanakakis et al., 2016	6
[316]	Ulusoy et al., 2013	3
[317]	Ulusoy et al., 2014	2
[318]	Uppenkamp et al., 1992	1
[319]	Valencia et al., 2008	4
[320]	Van Straaten et al., 2001;	1
[321]	Van Straaten; Groote; Oudesluys-Murphy, 1996	1
[322]	Vernier et al., 2014	7
[323]	Verrue; Smets, 2012	7
[324]	Vila et al., 2004	2
[325]	Voegeli, 1979	2
[326]	Vohr et al., 2002	5
[327]	Vos; Lagasse; Levêque, 2013	3
[328]	Vranješ et al., 2012	3
[329]	Wang; Xing; Liu, 2002	7
[330]	Wang et al., 2022	3
[331]	Watkin, 1999	2
[332]	Weichbold; Nekahm-Heis; Welzl-Müller, 2005	1
[333]	Welzl-Müller; Stephan, 2001	1
[334]	Widen et al., 2000	1
[335]	Wong et al., 2021	1
[336]	Wu; Huang; Kao, 1998	7
[337]	Xu et al., 2006	2
[338]	Xu et al., 2003	2
[339]	Xu; Li, 2005a	2
[340]	Xu; Li, 2005b	3
[341]	Xu; Cheng; Yang, 2011	2
[342]	Yao et al., 2013	4
[343]	Yigit et al., 2020	4
[344]	Yılmazer et al., 2016	1
[345]	Yoshinaga-Itano, 2003	3
[346]	Yoshinaga-Itano; Apuzzo, 1998	1
[347]	Yoshinaga-Itano; Gravel, 2001	1
[348]	Yousefi et al., 2013a	3
[349]	Yousefi et al., 2013b	3
[350]	Zeng et al., 2020	3
[351]	Zhang; Li; Zheng, 2014	2
[352]	Zhang et al., 2004	2
[353]	Zhao et al., 2003	1
[354]	Zwicker; Schorn, 1990	2
[355]	Zych et al., 2018	2

* 1. Wrong study design; 2. Wrong publication type; 3. Non-specific evaluation strategy; 4. Wrong population; 5. Wrong objective; 6. no response from the author; 7. Not found

REFERENCES

[1] Pérez MC, Gaya JA, Savio G, Perera M, Ponce de León M, Sánchez M. A 25-year review of Cuba’s screening program for early detection of hearing loss. MEDICC Rev. 2009;11(1):21-8. PMid:21483323.

[2] Abdul Hadi K, Salahaldin A, Al Qahtani A, Al Musleh Z, Al Sulaitin M, Bener A, et al. Universal neonatal hearing screening: six years of experience in Qatar. Qatar Med J. 2012;2012(2):42-50. https://doi.org/10.5339/qmj.2012.2.12. PMid:25003040.

[3] Abdulai ER, Baidoo KK, Jangu AA, Searyoh K, Ahonon Y. Otoacoustic emission hearing screening of newborns in Korle-bu teaching hospital, Ghana. Postgrad Med J Ghana. 2020;9(2):90-4. https://doi.org/10.60014/pmjg.v9i2.232.

[4] Abdullah A, Hazim MYS, Almyzan A, Jamilah AG, Roslin S, Ann MT, et al. Newborn hearing screening: experience in a Malaysian hospital. Singapore Med J. 2006;47(1):60-4. PMid:16397723.

[5] Adelola OA, Papanikolaou V, Gormley P, Lang J, Keogh IJ. Newborn hearing screening: A regional example for national care. Ir Med J. 2010;103(5):146-9. PMid:20666087.

[6] Ahmed S, Sheraz S, Malik SA, Ahmed NR, Malik SA, Farooq S, et al. Frequency of congenital hearing loss in neonates. J Ayub Med Coll Abbottabad. 2018;30(2):234-6. PMid:29938425.

[7] Aiyer RG, Parikh B. Evaluation of auditory brainstem responses for hearing screening of high-risk infants. Indian J Otolaryngol Head Neck Surg. 2009;61(1):47-53. https://doi.org/10.1007/s12070-009-0034-4. PMid:23120604.

[8] Al-Kandari JM, Alshuaib WB. Newborn hearing screening in Kuwait. Electromyogr Clin Neurophysiol. 2007;47(6):305-13. PMid:17918507.

[9] Alarcón Avila C, Saenz González AT, Vivas Gómez LF, Rivero Centeno MA, Silva Ortiz MJ, Salazar Vera LC, et al. Diagnostic performance and clinical follow-up of a universal newborn hearing screening program using OAE and ABR in a middle-income country. Int J Pediatr Otorhinolaryngol. 2025;197:112511. https://doi.org/10.1016/j.ijporl.2025.112511. PMid:40884912.

[10] Alberti PW, Hyde ML, Corbin H, Riko K, Abramovich S. An evaluation of bera for hearing screening in high-risk neonates. Laryngoscope. 1983;93(9):1115-21. https://doi.org/10.1288/00005537-198309000-00001. PMid:6888122.

[11] Allen B, Lambert G. Computerisation of V.R.O.A.: a double blind hearing screening technique. Aust J Audiol. 1990;12:11-5.

[12] Almenar Latorre A, Tapia Toca MC, Fernández Pérez C, Moro Serrano M. A combined neonatal hearing screening protocol. An Esp Pediatr. 2002;57(1):55-9. https://doi.org/10.1016/S1695-4033(02)77893-4. PMid:12139894.

[13] Alves De Sousa LC, De Toledo Piza M, Da Costa SS, Andrade MJ, Jaeger WLG. Child deafness: Early diagnosis and casuistic of Paparella Foundation. Revista Brasileira de Otorrinolaringologia 1996;62:9+11-12+14.

[14] Anderssen SH, Andresen J, Andersen R, Sponheim L. Universal neonatal hearing screening of infants with otoacoustic emissions. Tidsskr Nor Laegeforen. 2002;122(22):2187-9. PMid:12426894.

[15] Angrisani RMG, Suzuki MR, Pifaia GR, Testa JR, Sousa EC, Gil D, et al. PEATE automático em recém nascidos de risco: estudo da sensibilidade e especificidade. Rev CEFAC. 2012;14(2):223-33. https://doi.org/10.1590/S1516-18462011005000065.

[16] Ansari MS. Implementation of National Infant Screening for Hearing Program in India: Challenges, Opportunities and Suggested Framework. Indian J Otolaryngol Head Neck Surg. 2022;74(Suppl 3):4150-8. https://doi.org/10.1007/s12070-021-02873-6. PMid:36742505.

[17] Applebaum EL. Detection of hearing loss in children. Pediatr Ann. 1999;28(6):351-6. https://doi.org/10.3928/0090-4481-19990601-05. PMid:10382193.

[18] Aras Öztürk SE, Aktaş S, Karakurt LT, Develioğlu ÖN, Murat Z, Çetinkaya F, et al. The follow-up results of newborn hearing screening of gaziosmanpasa taksim research and training hospital. Turk Pediatri Ars. 2018;53(1):10-6. https://doi.org/10.5152/TurkPediatriArs.2018.5389. PMid:30083069.

[19] Ardic C. Newborn hearing screening outcomes from Rize; Turkey. Konuralp Tip Derg. 2017;9:41-5. https://doi.org/10.18521/ktd.292877.

[20] Arnold CL, Davis TC, Humiston SG, Bocchini JA Jr, Bass PF 3rd, Bocchini A, et al. Infant hearing screening: stakeholder recommendations for parent-centered communication. Pediatrics. 2006;117(5 Pt 2, Suppl 3):S341-54. https://doi.org/10.1542/peds.2005-2633N. PMid:16735261.

[21] Arslan S, Işik AU, Imamoğlu M, Topbaş M, Aslan Y, Ural A. Universal newborn hearing screening; automated transient evoked otoacoustic emissions. B-ENT. 2013;9(2):122-31. PMid:23909119.

[22] Arslan S, Isik A, Imamoglu M, Topbas M, Aslan Y, Ural A. Universal newborn hearing screening; automated transient evoked otoacoustic emissions. B-ENT. 2013;9(2):123-31. PMid:23909119.

[23] Ataoğlu E, Oğuz D, Mutluay K, Elevli M. Evaluation of newborn hearing screening results in a period of four years newborn hearing screening results. Zeynep Kamil Tip Bulteni. 2019;50:35-8. https://doi.org/10.16948/zktipb.447801.

[24] Azevedo MF. Avaliaçäo e acompanhamento audiológico de neonatos de risco. Acta AWHO. 1991;10:107-16.

[25] Azevedo RF, Paschoal CP, de Azevedo MF, dos Santos AM, Furia CLB. Avaliação da implantação de programa de triagem auditiva neonatal em hospital de nível secundário. Rev Paul Pediatr. 2004;22:77-84.

[26] Azizi A, Amirian F, Dargahi A, Beidaghi S, Mohammadi M. Evaluation of universal newborn hearing screening with TEOAE and ABR: a cross-sectional study with the literature review. Int J Trop Med. 2016;11:84-9.

[27] Babac S, Djerić D, Ivanković Z. Newborn hearing screening. Srp Arh Celok Lek. 2007;135(5-6):264-8. https://doi.org/10.2298/SARH0706264B. PMid:17633310.

[28] Back KEM, Ho SKY. Universal newborn hearing screening in Singapore general hospital. Proceedings of Singapore Healthcare. 2011;20:127. https://doi.org/10.1177/20101058110200S101.

[29] Backous DD. Diagnosis and management of hearing loss in infants and young children. Otolaryngol Clin North Am. 2002;35(4):xi-xii. https://doi.org/10.1016/S0030-6665(02)00057-9.

[30] Bagatto M, Moodie S, Fitzpatrick E, Kealey C, Campbell B, Aiken S. Status of early hearing detection and intervention programs in Canada: results from a country-wide survey. Can J Speech Lang Pathol Audiol. 2020;44:107-24.

[31] Bai JS, Gowda PRP, Naik SM, Somashekhar A. Hearing screening in high-risk neonates using distortion product oto-acoustic emission. Indian J Otolaryngol Head Neck Surg. 2024;76(1):620-5. https://doi.org/10.1007/s12070-023-04227-w. PMid:38440481.

[32] Bansal S, Gupta A, Nagarkar A. Transient evoked otoacoustic emissions in hearing screening programs-Protocol for developing countries. Int J Pediatr Otorhinolaryngol. 2008;72(7):1059-63. https://doi.org/10.1016/j.ijporl.2008.03.014. PMid:18479757.

[33] Barsky-Firkser L, Sun S. Universal newborn hearing screenings: a three-year experience. Pediatrics 1997;99(6):e4. https://doi.org/10.1542/peds.99.6.e4.

[34] Bartmeyer B, Navarini IGF, Carvalho ER. Infecção congênita pelo citomegalovírus: achados clínicos e desfecho em pacientes de um hospital pediátrico terciário de Florianópolis. Brazilian Journal of Sexually Transmitted Diseases. 2025;37:e1448. https://doi.org/10.5327/DST-2177-8264-1448.

[35] Begum A, Siddiqua H, Harshitha D, Unnisa F. Screening of hearing impairment in newborns with risk factors, detected by otoacoustic emission hearing test. International Journal of Pharmaceutical and Clinical Research. 2024;16:785-90.

[36] Bellia CGD, Artmann H, Marques JM, Lüders D, Gonçalves CGD. Brainstem Auditory Evoked Potentials in infants aged 1 to 24 months during a hearing health care service. Clinics (Sao Paulo). 2020;75:e1579. https://doi.org/10.6061/clinics/2020/e1579. PMid:33146347.

[37] Benito Orejas JI, Cano BR, Pérez DM, Fernández-Calvo JL, Gómez AA. Results of applying a universal protocol for early detection of hypoacusia in newborn infants for 42 months. Acta Otorrinolaringol Esp. 2008;59(3):96-101. https://doi.org/10.1016/S0001-6519(08)73274-6. PMid:18364200.

[38] Benito Orejas JI, Ramírez Cano B, Morais Pérez D, Fernández-Calvo JL, Almaraz Gómez A. Results of applying a universal protocol for early detection of hypoacusia in newborn infants for 42 months. Acta Otorrinolaringol Esp. 2008;59(3):96-101. https://doi.org/10.1016/S0001-6519(08)73274-6. PMid:18364200.

[39] Benito Orejas JI, Silva Rico JC. Hearing loss. Identification and early intervention. Pediatria Integral. 2017;21:418-28.

[40] Berger R, Goeze A, Müller-Mazzotta J, Hanschmann H, Kadaifciu B, Eroglu E. Early diagnosis of infant hearing impairment after introduction of newborn hearing screening (UNHS). Laryngorhinootologie. 2012;91(10):637-40. https://doi.org/10.1055/s-0032-1316321. PMid:22773399.

[41] Bertoldi PM, Manfredi AKS, Mitre EI. Análise dos resultados da triagem auditiva neonatal no município de Batatais. Medicina (Ribeiräo Preto) 2017;50:150-7.

[42] Bevilacqua MC, Alvarenga Kde F, Costa OA, Moret AL. The universal newborn hearing screening in Brazil: from identification to intervention. Int J Pediatr Otorhinolaryngol. 2010;74(5):510-5. https://doi.org/10.1016/j.ijporl.2010.02.009. PMid:20303604.

[43] Bhalot L, Gupta Y, Kumar M, George SP, Godha S, Mundra RK. Effect of Neonatal Hearing Screening on Early Diagnosis of Sensorineural Hearing Loss. Indian J Otolaryngol Head Neck Surg. 2023;75(Suppl 1):809-14. https://doi.org/10.1007/s12070-022-03395-5. PMid:37206810.

[44] Bhatia P, Mintz S, Hecht BF, Deavenport A, Kuo AA. Early identification of young children with hearing loss in federally qualified health centers. J Dev Behav Pediatr. 2013;34(1):15-21. https://doi.org/10.1097/DBP.0b013e318279899c. PMid:23275054.

[45] Biaggio EPV, Bertuol B, David ALM, Costa LD, Melo Ã. Newborn hearing screening program: an outlook of detection to the intervention of hearing loss. Int Arch Otorhinolaryngol. 2015;19:S36.

[46] Bianchin G, Palma S, Polizzi V, Kaleci S, Stagi P, Cappai M, et al. A regional-based newborn hearing screening program: the Emilia-Romagna model after ten years of legislation. Ann Ig. 2022;35(3):297-307. http://doi.org/10.7416/ai.2022.2539. PMid:35861691.

[47] Biscegli TS, Bertoni A, Belucio IS, Marcos JMO, Matias TY, Cid FB. Triagem auditiva neonatal: estudo dos neonatos de um hospital-escola do interior do estado de São Paulo. Pediatr Mod. 2015;51(6).

[48] Bishnoi R, Baghel S, Agarwal S, Sharma S. Newborn Hearing Screening: time to Act! Indian J Otolaryngol Head Neck Surg. 2019;71(Suppl 2):1296-9. https://doi.org/10.1007/s12070-018-1352-1. PMid:31750168.

[49] Bonelli A, Marcarini L, Fontanella WM, Passerini S, Colombo F, Blotta P. Otoacustic emissions (TEOAEs) as a screening for neonatal sensorineural hearing loss: our experience. Otorinolaringologia Pediatrica. 2000;11:39-44.

[50] Botasso KD, Lima M, Correa CRS. Analysis of an outpatient child hearing health program: from screening to referral for rehabilitation. CODAS 2022;34(4):e20200403. https://doi.org/10.1590/2317-1782/20212020403.

[51] Botasso KC, Lima MCMP, Correa CRS. Análise de um programa de saúde auditiva infantil ambulatorial: da triagem ao encaminhamento para reabilitação. CoDAS. 2022;34(4):e20200403. https://doi.org/10.1590/2317-1782/20212020403. PMid:35137893.

[52] Botelho FA, Bouzada MC, Resende LM, Silva CF, Oliveira EA. Prevalence of hearing impairment in children at risk. Braz J Otorhinolaryngol. 2010;76(6):739-44. https://doi.org/10.1590/S1808-86942010000600012. PMid:21180942.

[53] Bradford BC, Baudin J, Conway MJ, Hazell JW, Stewart AL, Reynolds EO. Identification of sensory neural hearing loss in very preterm infants by brainstem auditory evoked potentials. Arch Dis Child. 1985;60(2):105-9. https://doi.org/10.1136/adc.60.2.105. PMid:4038866.

[54] Bradley M, Barbera JL. Hearing & Children: early detection-Only the beginning. Hear J. 2001;54(7):56. https://doi.org/10.1097/01.HJ.0000294113.60246.3c.

[55] Bravo AR, Krefft MM, Gómez YF, García TMF, Sandoval VP, Torrente AM. Indicadores de calidad del Programa de Detección Precoz de Hipoacusia Permanente del Hospital Padre Hurtado. Rev Otorrinolaringol Cir Cabeza Cuello. 2017;77(2):117-23. https://doi.org/10.4067/S0718-48162017000200001.

[56] Brockow I, Kummer P, Liebl B, Nennstiel-Ratzel U. Universal Newborn Hearing Screening (UNHS) - Is it possible to successfully implement it nationwide? Gesundheitswesen. 2011;73(8-9):477-82. https://doi.org/10.1055/s-0031-1285900. PMid:21887659.

[57] Brockow I, Kummer P, Liebl B, Nennstiel-Ratzel U. Universal newborn hearing screening (UNHS): is it possible to successfully implement it nationwide?. Gesundheitswesen 2011;73(08/09):477-82. https://doi.org/10.1055/s-0031-1285900.

[58] Bubbico L, Tognola G, Grandori F. Evolution of Italian universal newborn hearing screening programs. Ann Ig. 2017;29(2):116-22. https://doi.org/10.7416/ai.2017.2138. PMid:28244580.

[59] Bubbico L, Tognola G, Greco A, Grandori F. Universal newborn hearing screening programs in Italy: survey of year 2006. Acta Otolaryngol. 2008;128(12):1329-36. https://doi.org/10.1080/00016480802008165. PMid:18607902.

[60] Buser K, Bietendüwel A, Krauth C, Jalilvand N, Meyer S, Reuter G, et al. Model project of hearing screening in new-born in Hanover (preliminary results). Gesundheitswesen. 2003;65(3):200-3. https://doi.org/10.1055/s-2003-38515. PMid:12698391.

[61] Calcutt TL, Dornan D, Beswick R, Tudehope DI. Newborn hearing screening in Queensland 2009–2011: comparison of hearing screening and diagnostic audiological assessment between term and preterm infants. J Paediatr Child Health. 2016;52(11):995-1003. https://doi.org/10.1111/jpc.13281. PMid:27521761.

[62] Calevo MG, Mezzano P, Zullino E, Padovani P, Serra G, Zaccagnino V, et al. Ligurian experience on neonatal hearing screening: clinical and epidemiological aspects. Acta Paediatr. 2007;96(11):1592-9. https://doi.org/10.1111/j.1651-2227.2007.00475.x. PMid:17937684.

[63] Campos Banãles ME, López Campos D, Pérez B, López Aguado D. Correlation between otoacoustic emissions and auditory brainstem response. The relevance of using them in combination. Acta Otorrinolaringologica Espanola. 2003;54:667-70. https://doi.org/10.1016/S0001-6519(03)78465-9.

[64] Campos ME, López Campos D, Pérez B, López Aguado D. Correlation between otoacoustic emissions and BAEP. The importance of their combined use]. Acta Otorrinolaringol Esp 2003;54(10):667-70.

[65] Canale A, Favero E, Lacilla M, Recchia E, Schindler A, Roggero N, et al. Age at diagnosis of deaf babies: a retrospective analysis highlighting the advantage of newborn hearing screening. Int J Pediatr Otorhinolaryngol. 2006;70(7):1283-9. https://doi.org/10.1016/j.ijporl.2006.01.008. PMid:16488484.

[66] Cao-Nguyen MH, Kos MI, Guyot JP. Benefits and costs of universal hearing screening programme. Int J Pediatr Otorhinolaryngol. 2007;71(10):1591-5. https://doi.org/10.1016/j.ijporl.2007.07.008. PMid:17719096.

[67] Cardoso LF, Bissani C, Pinheiro MMC, Viveiros CM, Carreirão W Fo. Análise da implantação de programa de triagem auditiva neonatal em um hospital universitário. Rev Bras Otorrinolaringol. 2009;75(2):237-44. https://doi.org/10.1590/S0034-72992009000200013.

[68] Cassidy JW, Ditty KM. Gender differences among newborns on a transient otoacoustic emissions test for hearing. J Music Ther. 2001;38(1):28-35. https://doi.org/10.1093/jmt/38.1.28. PMid:11407963.

[69] Ceccato C, Chirico G, Mastricci N, Precerutti G, Rondini G. DPOAE screening on discharging infants in local NICU. In: McCafferty WG, editor. Sydney ’97: XVI World Congress of Otorhinolaryngology Head and Neck Surgery; 1997 Mar 2-7; Sydney, Australia. Bologna: Monduzzi Editore; 1997. p. 21-5.

[70] Černý M, Zoban P, Groh D, Brabec R, Vejvalka J, Kabelka Z, et al. Screening for hearing impairment in newborns using transient evoked otoacoustic emissions. Ceskoslov Pediatr. 2003;58:700-4.

[71] Chadha S, Bais AS. Auditory brainstem responses in high risk and normal newborns. Indian J Pediatr. 1997;64(6):777-84. https://doi.org/10.1007/BF02725499. PMid:10771920.

[72] Champion S. Assessment of hearing in high risk infants, using brainstem evoked response audiometry. Indian J Otolaryngol Head Neck Surg. 2021;73(3):383-8. https://doi.org/10.1007/s12070-020-02363-1. PMid:34471628.

[73] Chang HW, Dillon H, Carter L, van Dun B, Young ST. The relationship between cortical auditory evoked potential (CAEP) detection and estimated audibility in infants with sensorineural hearing loss. Int J Audiol. 2012;51(9):663-70. https://doi.org/10.3109/14992027.2012.690076. PMid:22873205.

[74] Chen G, Yi X, Chen P, Dong J, Yang G, Fu S. A large-scale newborn hearing screening in rural areas in China. Int J Pediatr Otorhinolaryngol. 2012;76(12):1771-4. https://doi.org/10.1016/j.ijporl.2012.08.021. PMid:22954384.

[75] Chen X, Yuan M, Lu J, Zhang Q, Sun M, Chang F. Assessment of universal newborn hearing screening and intervention in Shanghai, China. Int J Technol Assess Health Care. 2017;33(2):206-14. https://doi.org/10.1017/S0266462317000344. PMid:28583223.

[76] Chibisova SS, Tsygankova ER, Markova TG, Rumiantseva MG. The universal audiological screening of newborn infants: achievements and challenges. Vestn Otorinolaringol. 2014;(2):49-53. PMid:24781172.

[77] Chiriboga LF, Sideri KP, Ferraresi Rodrigues Figueiredo SN, Monteiro Pinto ES, Chiriboga Arteta LM. Outcomes of a universal neonatal hearing screening program of 9941 newborns over a one-year period in Campinas, Brazil. Int J Pediatr Otorhinolaryngol. 2021;148:110839. https://doi.org/10.1016/j.ijporl.2021.110839. PMid:34274888.

[78] Choi KY, Park SK, Choi S, Chang J. Analysis of Newborn Hearing Screening Results in South Korea after National Health Insurance Coverage: A Nationwide Population-Based Study. Int J Environ Res Public Health. 2022;19(22):15052. https://doi.org/10.3390/ijerph192215052. PMid:36429776.

[79] Chu CW, Chen YJ, Lee YH, Jaung SJ, Lee FP, Huang HM. Government-funded universal newborn hearing screening and genetic analyses of deafness predisposing genes in Taiwan. Int J Pediatr Otorhinolaryngol. 2015;79(4):584-90. https://doi.org/10.1016/j.ijporl.2015.01.033. PMid:25724631.

[80] Chung YS, Oh SH, Park SK. Results of a government-supported newborn hearing screening pilot project in the 17 cities and provinces from 2014 to 2018 in Korea. J Korean Med Sci. 2020;35(31):e251. https://doi.org/10.3346/jkms.2020.35.e251. PMid:32776720.

[81] Chung YS, Oh SH, Park SK. Results of a government-supported newborn hearing screening pilot project in the 17 cities and provinces from 2014 to 2018 in Korea. J Korean Med Sci. 2020;35(31):e251. https://doi.org/10.3346/jkms.2020.35.e251. PMid:32776720.

[82] Cianfrone F, Mammarella F, Ralli M, Evetovic V, Pianura CM, Bellocchi G. Universal newborn hearing screening using A-TEOAE and A-ABR: the experience of a large public hospital. J Neonatal Perinatal Med. 2018;11(1):87-92. https://doi.org/10.3233/NPM-181744. PMid:29689750.

[83] Cianfrone F, Ubah F, Cazzaniga C, Abuhajar S, Ralli M, Angeletti D, et al. Evaluation of newborn hearing screening program in aSl roma 1, rome, italy. Otorhinolaryngology. 2021;71(2):95-9. https://doi.org/10.23736/S2724-6302.20.02335-8.

[84] Colella-Santos MF, Sartorato EL, Tazinazzio TG, de Françozo MCF, do Couto CM, Castilho AM, et al. An auditory health program for neonates in ICU and/or intermediate care settings. Braz J Otorhinolaryngol. 2013;79(6):709-15. https://doi.org/10.5935/1808-8694.20130130. PMid:24474482.

[85] Colella-Santos MF, Sartorato EL, Tazinazzio TG, Françozo MFC, Couto CM, Castilho AM, et al. Programa de saude auditiva em neonatos que permaneceram em UTI e/ou cuidados intermediarios. Braz j Otorhinolaryngol (Impr). 2013;79(6):709-15. https://doi.org/10.5935/1808-8694.20130130.

[86] Coll J, Régnier C. Early auditory potentials in the detection of deafness. Rev Laryngol Otol Rhinol (Bord). 1988;109(4):325-6. PMid:3238194.

[87] Collins A, Beswick R, Driscoll C, Kei J. Clinical characteristics of infants identified with a conductive hearing loss through universal newborn hearing screening: a population-based sample. Int J Pediatr Otorhinolaryngol. 2022;161:111268. https://doi.org/10.1016/j.ijporl.2022.111268. PMid:35964490.

[88] Costa BM, Grawer RS, Soldera CLC, Machado MS, Benvenutti SK. Newborn hearing screening in philanthropic hospital in porto alegre. Int Arch Otorhinolaryngol. 2015;19:S93.

[89] Cox LC, Toro MR. Evolution of a universal infant hearing screening program in an inner city hospital. Int J Pediatr Otorhinolaryngol. 2001;59(2):99-104. https://doi.org/10.1016/S0165-5876(01)00462-1. PMid:11378184.

[90] Cundall J. Universal newborn hearing screening in Tennessee. Tennessee Medicine : Journal of the Tennessee Medical Association. 1997;90(9):370-1. PMid:9308393.

[91] Curcin OA, Sitka U, Rasinski C. Results of screening Very Low Birthweight (VLBW) infants’ hearing. Pediatrics and Related Topics. 1998;36:469-79.

[92] Curnock DA. Identifying hearing impairment in infants and young children. BMJ. 1993;307(6914):1225-6. https://doi.org/10.1136/bmj.307.6914.1225. PMid:8281050.

[93] Da Silva LS, Ribeiro GE, Da Silva DPC, Montovani JC. Outcomes of automated auditory evoked potential realized in different places and factors associated with refer cases. Int Arch Otorhinolaryngol. 2017;21:S104.

[94] Dalzell LE, Orlando M, MacDonald M, Berg A, Bradley M, Cacace A, et al. The New York State universal newborn hearing screening demonstration project: ages of hearing loss identification, hearing aid fitting, and enrollment in early intervention. Ear Hear. 2000;21(2):118-30. https://doi.org/10.1097/00003446-200004000-00006. PMid:10777019.

[95] Dantas MB, Anjos CA, Camboim ED, Pimentel Mde C. Results of a neonatal hearing screening program in Maceió. Braz J Otorhinolaryngol. 2009;75(1):58-63. https://doi.org/10.1016/S1808-8694(15)30832-6. PMid:19488561.

[96] Davis A, Hind S. The newborn hearing screening programme in England. Int J Pediatr Otorhinolaryngol. 2003;67(Suppl 1):S193-6. https://doi.org/10.1016/j.ijporl.2003.08.024. PMid:14662193.

[97] Daykhes NA, Grigor’eva EA, Nazarochkin YV, Davydov VM, Kuznetsov AO. The results of universal audiological screening of newborn infants in the Astrakhan region. Vestn Otorinolaringol 2017;82(2):16-8. https://doi.org/10.17116/otorino201782216-18.

[98] De Barros Boishardy A, Lenoir FM, Brami P, Kapella M, Obstoy MF, Amstutz-Montadert I, et al. Universal hearing screening: 10,835 newborns tested in maternity wards of the geographical Department of Eure, France. Annales d’oto-Laryngologie et de Chirurgie Cervico Faciale : Bulletin de La Société d’oto-Laryngologie. Hop Paris. 2005;122(5):223-30. https://doi.org/10.1016/S0003-438X(05)82353-9. PMid:16439932.

[99] De González Dios J, Mollar Maseres J. Universal screening for neonatal hearing loss: an evaluation of the program as opposed to an evaluation of the test. Acta Otorrinolaringologica Espanola. 2005;56:331-4. https://doi.org/10.1016/S0001-6519(05)78625-8.

[100] De Leenheer EM, Janssens S, Padalko E, Loose D, Leroy BP, Dhooge IJ. Etiological diagnosis in the hearing impaired newborn: proposal of a flow chart. Int J Pediatr Otorhinolaryngol. 2011;75(1):27-32. https://doi.org/10.1016/j.ijporl.2010.05.040. PMid:21047691.

[101] De Mattos WM, Cardoso LF, Bissani C, Pinheiro MMC, Viveiros CM, Carreirão Filho W. Newborn hearing screening program implantation analysis at a university hospital. Braz J Otorhinolaryngol. 2009;75(2):237-44. https://doi.org/10.1016/S1808-8694(15)30784-9. PMid:19575110.

[102] Delgado Domínguez JJ. Early detection of hearing loss in children. Pediatria de Atencion Primaria. 2011;13:279-97.

[103] Deng X, Ema S, Mason C, Nash A, Carbone E, Gaffney M. Receipt and timeliness of newborn hearing screening and diagnostic services among babies born in 2017 in 9 states. J Public Health Manag Pract. 2022;28(1):E100-8. https://doi.org/10.1097/PHH.0000000000001232. PMid:32956290.

[104] Desloovere C, Lemmens W, Feenstra L, Debruyne F, Van Kerschaver E, Offeciers FE, et al. Universal hearing screening of newborns in Flanders: from dreaming to reality? (multiple letters). Tijdschr Geneeskd. 2000;56:1203. https://doi.org/10.2143/TVG.56.16.1000836.

[105] Dlouhá O, Černý L, Hrdličková M, Jedlička I, Puchmajer P, Vohradník M. Auditory screening in high-risk infants. Otorinolaryngologie a Foniatrie. 2002;51:4-7.

[106] Downs MP. Universal newborn hearing screening - The Colorado story. Int J Pediatr Otorhinolaryngol. 1995;32(3):257-9. https://doi.org/10.1016/0165-5876(95)01183-C. PMid:7665273.

[107] Duci AR, Pons VA, Porta CL, Moya BA, Salomón GJ, Martínez CH, et al. Detección universal de hipoacusias en recién nacidos. Rev Otorrinolaringol Cir Cabeza Cuello. 2000;60:143-50.

[108] Durante AS, Carvallo RM, da Costa FS, Soares JC. Characteristics of transient evoked otoacoustic emissions in newborn hearing screening program. Pró-Fono R Atual Cient. 2005;17(2):133-40. https://doi.org/10.1590/s0104-56872005000200002.

[109] Durante AS, Carvallo RMM, Costa MTZ, Ianciarullo MA, Voegels RL, Takahashi GM, et al. A implementação de programa de triagen auditiva neonatal universal em um hospital universitário brasileiro. Pediatria (Säo Paulo). 2004;26:78-84.

[110] Eibenstein A, Varakliotis T, Saccoccio A, Cisternino S, La Gamma R, Mattei A, et al. Newborn hearing screening with TEOAE: our experience on 4759 infants. Otorinolaringologia. 2014;64:27-32.

[111] Eiserman WD, Hartel DM, Shisler L, Buhrmann J, White KR, Foust T. Using otoacoustic emissions to screen for hearing loss in early childhood care settings. Int J Pediatr Otorhinolaryngol. 2008;72(4):475-82. https://doi.org/10.1016/j.ijporl.2007.12.006. PMid:18276019.

[112] Elpers J, Lester C, Shinn JB, Bush ML. Rural family perspectives and experiences with early infant hearing detection and intervention: a qualitative study. J Community Health. 2016;41(2):226-33. https://doi.org/10.1007/s10900-015-0086-1. PMid:26316007.

[113] Farhat AS, Ghasemi MM, Akhondian J, Mohamadzadeh A, Esmaeili H, Amiri R, et al. Assessment of the prevalence of hearing impairment in neonates born in Imam Reza, Ghaem and OM-Albanin Hospitals of Mashhad. Iran J Nematol. 2014;5:17-20.

[114] Felden GQP, Didoné DD, Sleifer P, Gomes E, Etcheverria SK, França MCT, et al. Auditory monitoring in children with risk indicator for hearing loss. Int Arch Otorhinolaryngol. 2017;21:S8.

[115] Fernandes JC, Nozawa MR. Estudo da efetividade de um programa de triagem auditiva neonatal universal. Cien Saude Colet. 2010;15(2):353-61. https://doi.org/10.1590/S1413-81232010000200010.

[116] Fernandez Martinez MD, Bosch Gimenez VM, Recuero Fernandez E, Vicente Santos T, Borrajo Guadarrama E, Canteras Jordana M. Evaluation of audition in children who had a birth weight less than 1,201 grams. An Esp Pediatr. 1995;43:184-6.

[117] Feroz S, Irshad M, Shamsi SA, Sohail E, Hamid A, Rohan M. Evaluation of optimal postnatal timing for the screening of neonatal hearing via transient evoked oto-acoustic emission (TEOAE): a retrospective cross-sectional study. Ir J Med Sci. 2025;95(2):961-8. https://doi.org/10.1007/s11845-025-04238-2. PMid:41432879.

[118] Ferro LM, Tanner G, Erler SF, Erickson K, Dhar S. Comparison of universal newborn hearing screening programs in Illinois hospitals. Int J Pediatr Otorhinolaryngol. 2007;71(2):217-30. https://doi.org/10.1016/j.ijporl.2006.10.004. PMid:17097746.

[119] Findlen UM, Malhotra PS, Hunter LL. Early hearing detection. Clin Perinatol. 2025;52(3):509-21. https://doi.org/10.1016/j.clp.2025.06.009. PMid:40850713.

[120] Fink N, Goeze A, Zaretsky E, Fink A, Reimann K, Hey C. Follow-up II of newborn hearing screening: evaluation of a follow-up II facility after implementation of newborn hearing screening in Germany. HNO. 2022;70(3):179-86. https://doi.org/10.1007/s00106-021-01098-x. PMid:34448878.

[121] Fitzpatrick EM, Durieux-Smith A, Whittingham J. Clinical practice for children with mild bilateral and unilateral hearing loss. Ear Hear. 2010;31(3):392-400. https://doi.org/10.1097/AUD.0b013e3181cdb2b9. PMid:20054278.

[122] François M, Hautefort C, Nasra Y, Zohoun S. Evolution of age at diagnosis of congenital hearing impairment. Eur Ann Otorhinolaryngol Head Neck Dis. 2011;128(2):59-63. https://doi.org/10.1016/j.anorl.2010.10.009. PMid:21295536.

[123] Fujikawa S, Yoshinaga-Itano C. Current status of universal newborn hearing screening. Curr Opin Otolaryngol Head Neck Surg. 2000;8(5):404-8. https://doi.org/10.1097/00020840-200010000-00009.

[124] Fukushima K, Mimaki N, Fukuda S, Nishizaki K. Pilot study of universal newborn hearing screening in Japan: district-based screening program in Okayama. Ann Otol Rhinol Laryngol. 2008;117(3):166-71. https://doi.org/10.1177/000348940811700302. PMid:18444475.

[125] Gáborján A, Götze J, Küstel J, Kecskeméti N, Baranyi I, Csontos F, et al. Verification results of objective newborn hearing screening. Orv Hetil. 2019;160(47):1850-5. https://doi.org/10.1556/650.2019.31604. PMid:31736348.

[126] Gaborján A, Katona G, Szabó M, Muzsik B, Kustel M, Horváth M, et al. Universal newborn hearing screening with automated auditory brainstem response (AABR) in Hungary: 5-year experience in diagnostics and influence on the early intervention. Eur Arch Oto-Rhino-Laryngol. 2022;279(12):5647-54. https://doi.org/10.1007/s00405-022-07441-4. PMid:35767058.

[127] Galhotra A, Sahu P. Challenges and solutions in implementing hearing screening program in India. Indian J Community Med. 2019;44(4):299-302. https://doi.org/10.4103/ijcm.IJCM_73_19. PMid:31802788.

[128] Garbaruk ES, Fedorova LA, Savenko IV, Vikhnina SM, Boboshko MY. Childhood hearing screening: achievements, difficulties, and possible ways to improve. Vestn Otorinolaringol. 2021;86(1):82-9. https://doi.org/10.17116/otorino20218601182. PMid:33720658.

[129] Gavilan Cellie I. Auditory screening of newborns: new procedures. Rev Laryngol Otol Rhinol (Bord). 1993;114:239-41. PMid:8029542.

[130] Geal-Dor M, Levi H, Elidan Y, Arad I. The hearing screening program for newborns with otoacoustic emission for early detection of hearing loss. Harefuah. 2002;141(7):586-90, 668. PMid:12187552.

[131] Gellrich D, Gröger M, Echternach M, Eder K, Huber P. Neonatal hearing screening - does failure in TEOAE screening matter when the AABR test is passed? Eur Arch Oto-Rhino-Laryngol. 2024;281(3):1273-83. https://doi.org/10.1007/s00405-023-08250-z. PMid:37831131.

[132] Gentiletti-Faenze GG, Yañez-Suarez O, Cornejo-Crus JM. Evaluation of automatic identification algorithms for auditory brainstem response used in universal hearing loss *screening.* In: Proceedings of the 25th Annual International Conference of the IEEE Engineering in Medicine and Biology Society (IEEE Cat. No.03CH37439); 2003; Cancun, Mexico. USA: IEEE; 2003. vol. 3, p. 2857-60. https://doi.org/10.1109/IEMBS.2003.1280514.

[133] Georgescu M, Vladareanu S. Early audiological diagnostic in newborn and infant. GinecoEu. 2015;11(3):131-3. https://doi.org/10.18643/gieu.2015.131.

[134] Giordano T, Marchegiani AM, Germiller JA. Children With Sensorineural Hearing Loss And Referral To Early Intervention. ORL Head Neck Nurs. 2015;33(3):10-4. PMid:26427185.

[135] Godoy BC, Bustamante ML. Evaluación de la fase de screening auditivo en menores con factores de riesgo. Rev Otorrinolaringol Cir Cabeza Cuello. 2006;66(2):103-6. https://doi.org/10.4067/S0718-48162006000200005.

[136] González De Aledo Linos A, Bonilla Miera C, Morales Angulo C, Gómez Da Casa F, Barrasa Benito J. Universal newborn hearing screening in Cantabria (Spain): results of the first two years. Anales de Pediatria. 2005;62(2):135-40. https://doi.org/10.1157/13071310. PMid:15701309.

[137] González de Dios J, Mollar Maseres J, Rebagliato Russo M. Evaluation of a universal screening program for hypacusia in neonates. An Pediatr (Barc) 2005;63(3):230-7. https://doi.org/10.1157/13078486.

[138] González Díaz CA, Chapa González C, Laciar Leber E, Vélez HA, Puente NP, Flores D-L, et al., editors. VIII Latin American Conference on Biomedical Engineering and XLII National Conference on Biomedical Engineering. In: Proceedings of CLAIB-CNIB 2019; 2019 Oct 2-5; Cancún, México. Cham: Springer International Publishing; 2020. vol. 75. https://doi.org/10.1007/978-3-030-30648-9.

[139] Gonzalez LR, Díaz ALZ, Mora OC. Effectiveness of “Universal Newborn Hearing Screening program” of Caja Costarricense del Seguro Social, in detecting deafness in children, from 2016 to 2018. Poblac Salud Mesoam. 2022;19(2):204-23. http://dx.doi.org/10.15517/psm.v0i19.47144.

[140] González L, Fernández de Soto JM, Torres MI. Current status of the programs for detection of hearing loss in children younger than six months in Cali. Colomb Med (Cali). 2012;43(1):73-81. https://doi.org/10.25100/cm.v43i1.1061.

[141] Gordo A, Parlato EM, de Azevedo MF, Guedes ZCF. Triagem auditiva em bebês de 2 a 12 meses. Pro Fono. 1994;6:7-13.

[142] Granell J, Martin G. Early detection and intervention on infant hearing loss. In: Dupont JP, editor. Hearing loss: classification, causes and treatment. New York: Nova Science Publishers; 2011. p. 189-212.

[143] Greczka G, Wróbel M, Dąbrowski P, Mikołajczak K, Szyfter W. Universal Neonatal Hearing Screening Program in Poland–10-year summary. Otolaryngol Pol. 2015;69(3):1-5. https://doi.org/10.5604/00306657.1156325. PMid:26388243.

[144] Greczka G, Zych M, Szyfter W, Wróbel M. Analysis of changes in the Polish Universal Neonatal Hearing Screening Program over 15 years. Otolaryngol Pol. 2018;72(2):13-9. https://doi.org/10.5604/01.3001.0011.7247. PMid:29748454.

[145] Grégoire J. Results of a deafness screening program for infants: a pilot project. Can J Public Health. 1996;87(5):339-42. PMid:8991754.

[146] Greville KA, Keith WJ. The effectiveness of two infant hearing screening programmes in New Zealand. Scand Audiol. 1978;7(3):139-45. https://doi.org/10.3109/01050397809076280. PMid:756078.

[147] Groh D, Kabelka Z, Jurovcik M, Koprivova H, Zoban P, Cerny M. Results of a screening programme of hearing disorders in neonates in the Faculty Hospital Prague - Motol in 1997 - 1998. Otorinolaryngologie a Foniatrie. 1999;48:3-6.

[148] Guastini L, Mora R, Dellepiane M, Santomauro V, Mora M, Rocca A, et al. Evaluation of an automated auditory brainstem response in a multi-stage infant hearing screening. Eur Arch Oto-Rhino-Laryngol. 2010;267(8):1199-205. https://doi.org/10.1007/s00405-010-1209-z. PMid:20148257.

[149] Guilhoto LMFF, Quintal VS, Da Costa MTZ. Brainstem auditory evoked response in normal term neonates. Arq Neuropsiquiatr. 2003;61(4):906-8. https://doi.org/10.1590/S0004-282X2003000600003. PMid:14762588.

[150] Guimarães VC, Barbosa MA. Prevalência de alterações auditivas em recém-nascidos em hospital escola. Int Arch Otorhinolaryngol. 2012;16:179-85. https://doi.org/10.7162/S1809-97772012000200005.

[151] Gulati A, Sakthivel P, Singh I, Ramji S. The Hearing Status of Preterm Infant’s ≤ 34 Weeks as Revealed by Otoacoustic Emissions (OAE) Screening and Diagnostic Brainstem Evoked Response Audiometry (BERA): A Tertiary Center Experience. Indian J Otolaryngol Head Neck Surg. 2022;74(Suppl 1):178-83. https://doi.org/10.1007/s12070-020-01945-3. PMid:36032856.

[152] Halder AL, Mollah MAH, Baki MA, Khan S, Nahar J. Hearing impairment among the high-risk neonates: findings from a hospital-based targeted screening. Mymensingh Med J. 2025;34(2):400-7. PMid:40160057.

[153] Hall V, Brosch S, Hoffmann TK. Evaluation of universal newborn hearing screening and follow-up. Laryngorhinootologie. 2020;99(5):299-307. https://doi.org/10.1055/a-1114-6452. PMid:32131107.

[154] Hanschmann H, Berger R. Universal newborn hearing screening. New early recognition examination. Monatsschr Kinderheilkd. 2010;158(6):597-607. https://doi.org/10.1007/s00112-010-2203-7.

[155] Harrison WA, Dunnell JJ, Mascher K, Fletcher K, Vohr BR, Gorga MP, et al. Identification of Neonatal Hearing Impairment: experimental protocol and database management. Ear Hear. 2000;21(5):357-72. https://doi.org/10.1097/00003446-200010000-00004. PMid:11059698.

[156] Hatzopoulos S, Pelosi G, Petruccelli J, Rossi M, Vigi V, Chierici R, et al. Efficient otoacoustic emission protocols employed in a hospital-based neonatal screening program. Acta Otolaryngol. 2001;121(2):269-73. https://doi.org/10.1080/000164801300043802. PMid:11349794.

[157] Hayes D. Current status of universal newborn hearing screening. Curr Opin Otolaryngol Head Neck Surg. 2002;10(5):382-6. https://doi.org/10.1097/00020840-200210000-00010.

[158] Helge T, Werle E, Barnick M, Wegner C, Rühe B, Aust G, et al. Two-tier screening process (TEOAE/AABR) reduces recall rates in newborn hearing screening. HNO. 2005;53(7):655-60. https://doi.org/10.1007/s00106-004-1124-y. PMid:15565423.

[159] Hergils L. How do we identify hearing impairment in early childhood?. Acta Paediatr Suppl. 2000;89(434):12-6. https://doi.org/10.1111/j.1651-2227.2000.tb03090.x. PMid:11055312.

[160] Hoffmann A, Deuster D, Rosslau K, Knief A, Am Zehnhoff-Dinnesen A, Schmidt CM. Feasibility of 1000Hz tympanometry in infants: tympanometric trace classification and choice of probe tone in relation to age. Int J Pediatr Otorhinolaryngol. 2013;77(7):1198-203. https://doi.org/10.1016/j.ijporl.2013.05.001. PMid:23726955.

[161] Hosforddunn H, Johnson S, Simmons FB, Malachowski N, Low K. Infant hearing screening - program implementation and validation. Ear Hear. 1987;8(1):12-20. https://doi.org/10.1097/00003446-198702000-00003. PMid:3549402.

[162] Huang CM, Yang IY, Julie Ma YC, Lin GSF, Yang CC, Tsai HT, et al. The effectiveness of the promotion of newborn hearing screening in Taiwan. Int J Pediatr Otorhinolaryngol. 2014;78(1):14-8. https://doi.org/10.1016/j.ijporl.2013.10.005. PMid:24300945.

[163] Huang EY, DeSell M, White AD, Walsh J, Jenks CM. Results and patient satisfaction from an early access infant hearing detection clinic. Int J Pediatr Otorhinolaryngol. 2023;164:111396. https://doi.org/10.1016/j.ijporl.2022.111396. PMid:36450185.

[164] Huang L, Cai Z, Zhang H, Peng S, Wu D, Wang L, et al. [Study on multi-area universal newborn hearing screening in countryside of China]. Lin Chuang Er Bi Yan Hou Tou Jing Wai Ke Za Zhi. 2009;23(16):737-42. PMid:20041607.

[165] Huang L, Xiong F, Li J, Yang F. An analysis of hearing screening test results in 2291 premature infants of Chinese population. Int J Pediatr Otorhinolaryngol. 2017;95:15-9. https://doi.org/10.1016/j.ijporl.2017.01.027. PMid:28576525.

[166] Huarte Irujo A. Evaluación audiológica de la población infantil. Rev Chil Fonoaudiol 2003;4:17-35. https://doi.org/10.5354/0719-4692.2003.56613.

[167] Irfan M, Shuaib M, Imran M, Ahmed T, Saleem S, Khan HA. Outcomes of Universal Neonatal Hearing Screening Program in a Tertiary Care Hospital in Lahore. Ann Abbasi Shaheed Hosp Karachi Med Dent Coll. 2024;29(3):270-6. https://doi.org/10.58397/ashkmdc.v29i3.890.

[168] Iwanicka-Pronicka K, Radziszewska-Konopka M, Wybranowska A, Churawski Ł. Analysis of specificity and sensitivity of Polish “Universal Newborn Hearing Screening Program.”. Otolaryngol Pol. 2008;62(1):88-95. https://doi.org/10.1016/S0030-6657(08)70215-4. PMid:18637428.

[169] Izumets O, Nezhivenko T, Murashko T. Hearing loss among neonates, early diagnostics. Likars’ka Sprava. 2015:172-4.

[170] Jacob J, Kurien M, Kumar P, Krishnan L. Challenges of universal newborn hearing screening in a developing country-a double-edged sword. Indian J Otolaryngol Head Neck Surg. 2022;74(Suppl 1):395-401. https://doi.org/10.1007/s12070-020-02170-8. PMid:36032816.

[171] Jacobson CA, Jacobson JT. Follow-up services in newborn hearing screening programs. J Am Acad Audiol. 1990;1(4):181-6. PMid:2132602.

[172] Jacobson GP. Universal newborn hearing loss: screening, identification, intervention. Am J Audiol. 2001;10(2):52. https://doi.org/10.1044/1059-0889(2001/ed02). PMid:11808719.

[173] Jakubíková J, Kabátová Z, Závodná M. Identification of hearing loss in newborns by transient otoacoustic emissions. Int J Pediatr Otorhinolaryngol. 2003;67(1):15-8. https://doi.org/10.1016/S0165-5876(02)00285-9. PMid:12560144.

[174] Jakubikova J, Zavodna M, Kardosova A, Vicianova K. Auditory screening of neonates using an objective method–otoacoustic emissions. Bratisl Lek Listy. 1999;100(11):607-10. PMid:10758739.

[175] Januário GC, Lemos SMA, de Lima Friche AA, Alves CRL. Quality indicators in a newborn hearing screening service. Braz J Otorhinolaryngol. 2015;81(3):255-63. https://doi.org/10.1016/j.bjorl.2014.08.008. PMid:25596650.

[176] Jayne A, Fullante PB, Karisse T, Dariel A, Lenon M, Sunga MB, et al. Pilot Implementation of a Community-based, eHealth-enabled Service Delivery Model for Newborn Hearing Screening and Intervention in the Philippines. Acta Med Philipp. 2023;57(9):73-84. https://doi.org/10.47895/amp.v57i9.5332. PMid:39483802.

[177] Johnson JL, White KR, Widen JE, Gravel JS, Vohr BR, James M, et al. A multisite study to examine the efficacy of the otoacoustic emission/automated auditory brainstem response newborn hearing screening protocol: introduction and overview of the study. Am J Audiol. 2005;14(2):S178-85. https://doi.org/10.1044/1059-0889(2005/020). PMid:16489862.

[178] Kanji A, Khoza-Shangase K. The occurrence of high-risk factors for hearing loss in very-low-birth-weight neonates: a retrospective exploratory study of targeted hearing screening. S Afr J Commun Disord. 2012;59(1):3-7. https://doi.org/10.4102/sajcd.v59i1.16. PMid:23409613.

[179] Kanji A, Krabbenhoft K. Audiological follow-up in a risk-based newborn hearing screening programme: an exploratory study of the influencing factors. S Afr J Commun Disord. 2018;65(1):e1-7. https://doi.org/10.4102/sajcd.v65i1.587. PMid:30456962.

[180] Kawata A, Kumagami H, Hara M, Sainoo Y, Takasaki K, Takahashi H, et al. Universal newborn hearing screening in nagasaki prefecture - Experience for 4. 5 years. Pract Otorhinolaryngol (Basel). 2011;104(12):849-54. https://doi.org/10.5631/jibirin.104.849.

[181] Keefe DH, Folsom RC, Gorga MP, Vohr BR, Bulen JC, Norton SJ. Identification of Neonatal Hearing Impairment: ear-canal measurements of acoustic admittance and reflectance in neonates. Ear Hear. 2000;21(5):443-61. https://doi.org/10.1097/00003446-200010000-00009. PMid:11059703.

[182] Kemper AR. Universal newborn hearing screening improves quality of life for children with permanent hearing impairment. J Pediatr. 2011;158(5):859. https://doi.org/10.1016/j.jpeds.2011.03.011. PMid:21482247.

[183] Kerschner JE, Meurer JR, Conway AE, Fleischfresser S, Cowell MH, Seeliger E, et al. Voluntary progress toward universal newborn hearing screening. Int J Pediatr Otorhinolaryngol. 2004;68(2):165-74. https://doi.org/10.1016/j.ijporl.2003.10.010. PMid:14725983.

[184] Kiese-Himmel C, Schroff J, Kruse E. Identification and diagnostic evaluation of hearing impairments in early childhood in German-speaking infants. Eur Arch Oto-Rhino-Laryngol. 1997;254(3):133-9. https://doi.org/10.1007/BF02471276. PMid:9112033.

[185] Kim JH, Lee KB, Koo YC, Hong SA, Lee Y, Son EJ. Evaluation of transient evoked otoacoustic emission in the newborn hearing screening program in neonatal intensive care unit. Korean J Audiol. 2011;15:81-4.

[186] Koester M, Nekahm-Heis D, Zorowka P. Early detection of hearing impairment in the newborn. MMW Fortschr Med. 2002;144:39-40. PMid:12577738.

[187] Konukseven O, Genc A, Muderris T, Kayikci MK, Turkyilmaz D, Ozturk B, et al. Can automated auditory brainstem response be used as an initial stage screening test in newborn hearing screening programs? J Int Adv Otol. 2010;6:231-8.

[188] Korres SG, Balatsouras DG, Nikolopoulos T, Korres GS, Ferekidis E. Making universal newborn hearing screening a success. Int J Pediatr Otorhinolaryngol. 2006;70(2):241-6. https://doi.org/10.1016/j.ijporl.2005.06.010. PMid:16029898.

[189] Korres S, Nikolopoulos TP, Peraki EE, Tsiakou M, Karakitsou M, Apostolopoulos N, et al. Outcomes and efficacy of newborn hearing screening: strengths and weaknesses (success or failure?). Laryngoscope. 2008;118(7):1253-6. https://doi.org/10.1097/MLG.0b013e31816d726c. PMid:18401271.

[190] Krauss MK, Heider CC, Sierra GM, Ribalta LG. Estrategias para mejorar el seguimiento del Programa de Evaluación Auditiva Neonatal Universal. Rev Otorrinolaringol Cir Cabeza Cuello. 2013;73(2):140-5. https://doi.org/10.4067/S0718-48162013000200005.

[191] Krishnan LA. Universal newborn hearing screening follow-up: A university clinic perspective. Am J Audiol. 2009;18(2):89-98. https://doi.org/10.1044/1059-0889(2009/09-0003). PMid:19704112.

[192] Kumar A, Gupta SC, Sinha VR. Universal hearing screening in newborns using otoacoustic emissions and brainstem evoked response in eastern Uttar Pradesh. Indian J Otolaryngol Head Neck Surg. 2017;69(3):296-9. https://doi.org/10.1007/s12070-017-1081-x. PMid:28929058.

[193] Lan H, Liu M, Huang C, Ren J, Huang Y, Jiang F, et al. Evaluation of the current situation and quality of neonatal hearing screening from hearing screening practitioners’ perspective: a cross-sectional study. J Matern Fetal Neonatal Med. 2024;37(1):2317412. https://doi.org/10.1080/14767058.2024.2317412. PMid:38369473.

[194] Langagne T, Schmidt P, Leveque M, Chays A. Universal hearing screening in the Champagne-Ardenne regions: results and consideration after 55 000 births from January 2004 to June 2007. Rev Laryngol Otol Rhinol (Bord). 2008;129(3):153-8. PMid:19694157.

[195] Lee-Ramos CM, Francis A, Collanto AS, John P, Sison LG, Chiong CM. Hearing screening through frequency analysis of auditory brainstem response using PhysioNet Data. Acta Med Philipp. 2023;57(9):32-8. https://doi.org/10.47895/amp.v57i9.4336. PMid:39483803.

[196] Leonhardt A, Müller M. The Newborn hearing-screening: setting a direction for life. Sprache Stimme Gehor. 2008;32:12-7. https://doi.org/10.1055/s-2007-1022556.

[197] Lewis DR, Racai R, Bevilacqua MC. Identificaçäo precoce de deficiência auditiva 1987:113-8.

[198] Lewis DR, Marone SAM, Mendes BCA, Cruz OLM, Nóbrega M. Comitê multiprofissional em saúde auditiva: COMUSA. Braz J Otorhinolaryngol. 2010;76:121-8.

[199] Lim SB, Daniel LM. Establishing a universal newborn hearing screening programme. Ann Acad Med Singap. 2008;37(12, Suppl):63-5. PMid:19904454.

[200] Lima GML, Marba STM, Santos MFC. Avaliação auditiva em recém-nascidos internados em unidades de terapia intensiva e de cuidados intermediários: triagem e acompanhamento ambulatorial. Rev ciênc méd (Campinas) 2005;14:147-56.

[201] Lima MCMP, Rossi TRF, Françozo MFC, Marba ST, Lima GML, Santos MFC. Detecção de perdas auditivas em neonatos de um hospital público. Rev Soc Bras Fonoaudiol. 2010;15(1):1-6. https://doi.org/10.1590/S1516-80342010000100003.

[202] Lima PT, Goldbach MG, Monteiro MC, Ribeiro MG. A triagem auditiva neonatal na Rede Municipal do Rio de Janeiro, Brasil. Ciênc Saúde Colet. 2015;20:57-63. https://doi.org/10.1590/1413-81232014201.21002013.

[203] Ling Q, Huang Z, Zhou Y, Li M, Zhong J. 3,200 cases of neonatal hearing screening results and analysis of related factors. Lin Chuang Er Bi Yan Hou Tou Jing Wai Ke Za Zhi. 2015;29(22):1977-80. PMid:26911062.

[204] Liu P, Chen P, Wang Z, Wei Y, Le W, Ding G, et al. Analysis of TEOAE and AABR hearing screening and follow-up in NICU. Lin Chuang Er Bi Yan Hou Tou Jing Wai Ke Za Zhi. 2014;28(10):705-7. PMid:25129970.

[205] Liu Z, Bu X, Xing G, Lu L. A preliminary study of a hearing screening model for newborn. Zhonghua Er Bi Yan Hou Ke Za Zhi. 2001;36(4):292-4. PMid:12762000.

[206] Lopes BM, Bueno CD, Didoné DD, Sleifer P. Comparison between click and ce-chirp® stimuli in neonatal hearing screening. J Hum Growth Dev. 2020;30(2):260-5. https://doi.org/10.7322/jhgd.v30.10375.

[207] Lotfi Y, Movallali G. A universal newborn hearing screening in Iran. Iran Rehabil J. 2007;5:8-11.

[208] Low WK, Pang KY, Ho LY, Lim SB, Joseph R. Universal newborn hearing screening in Singapore: the need, implementation and challenges. Ann Acad Med Singap. 2005;34(4):301-6. https://doi.org/10.47102/annals-acadmedsg.V34N4p301. PMid:15937570.

[209] Lowell EL. Guidelines for the early detection of auditory deficiency. Bol Oficina Sanit Panam. 1960;49:467-70. PMid:13763822.

[210] Magnani C, Bacchi G, Borghini AM, Delmonte D, Fava G, Occasio AM, et al. Universal newborn hearing screening: the experience of the University Hospital of Parma. Acta Biomed. 2015;86(3):273-7. PMid:26694155.

[211] Magnusson M, Persson K, Sundelin C. The effectiveness of routine health examinations at 2, 6, 9 and 12 months of age: experiences based on data from a Swedish county. Child Care Health Dev. 2001;27(2):117-31. https://doi.org/10.1046/j.1365-2214.2001.00180.x. PMid:11251611.

[212] Mahmood Z, Dogar MR, Waheed A, Ahmad AN, Anwar Z, Abbasi SZ, et al. Screening programs for hearing assessment in newborns and children. Cureus. 2020;12(11):e11284. https://doi.org/10.7759/cureus.11284. PMid:33274158.

[213] Maluleke NP, Khoza-Shangase K, Kanji A. Hearing impairment detection and intervention in children from centre-based early intervention programmes. J Child Health Care. 2019;23(2):232-41. https://doi.org/10.1177/1367493518788477. PMid:30068223.

[214] Maniuc M, Chiaburu-Chiosa D. Auditory function assessment via audiological screening in children. Proc Natl Ent Head Neck Surg Conf. 2018:145-50.

[215] Marco Algarra J, Pitarch Ribes MI, Morant Ventura A. Early diagnosis of neonatal hearing loss. Acta Otorrinolaringol Esp. 1996;47(3):255-7. PMid:8924295.

[216] Marinho ACA, de Souza Pereira EC, Torres KKC, Miranda AM, Ledesma ALL. Evaluation of newborn hearing screening program. Rev Saude Publica. 2020;54:44. https://doi.org/10.11606/s1518-8787.2020054001643. PMid:32374803.

[217] Marn B. First model of the newborn screening for hearing impairment in Croatia. Paediatr Croat. 2000;44:77-80.

[218] Marn B. Early detection of hearing impairment in croatia - screening and diagnostics. Paediatr Croat. 2012;56:195-201.

[219] Martines F, Porrello M, Ferrara M, Martines M, Martines E. Newborn hearing screening project using transient evoked otoacoustic emissions: western Sicily experience. Int J Pediatr Otorhinolaryngol. 2007;71(1):107-12. https://doi.org/10.1016/j.ijporl.2006.09.011. PMid:17095100.

[220] Martines F, Salvago P, Cocuzza S, La Mattina E, Stefano M, Mucia M, et al. Essential of audiology: screening and post-screening. Ital J Pediatr. 2014;40.

[221] Martínez R, Benito JI, Condado MA, Morais D, Fernández-Calvo JL. Results of the application of the protocol for the early detection of hearing loss in high-risk neonates. An Otorrinolaringol Ibero Am. 2003;30(3):277-87. PMid:12918292.

[222] Martínez R, Benito Orejas JI, Condado MA, Morais D, Fernández Calvo JL. Results of 1 year’s application of a universal protocol for the early detection of hearing loss in neonates. Acta Otorrinolaringologica Espanola. 2003;54(5):309-15. https://doi.org/10.1016/S0001-6519(03)78419-2. PMid:12916474.

[223] Martínez-Pacheco MC, Ferrán de la Cierva L, García-Purriños FJ. Delayed diagnosis of childhood deafness: the value of false negatives in the Programme for Early Detection of Neonatal Hearing Loss. Acta Otorrinolaringol Esp. 2016;67(6):324-9. https://doi.org/10.1016/j.otoeng.2016.01.001. PMid:27061391.

[224] Mason JA, Herrmann KR. Universal infant hearing screening by automated auditory brainstem response measurement. Pediatrics. 1998;101(2):221-8. https://doi.org/10.1542/peds.101.2.221. PMid:9445495.

[225] Mathur NN, Dhawan R. An alternative strategy for universal infant hearing screening in tertiary hospitals with a high delivery rate, within a developing country, using transient evoked oto-acoustic emissions and brainstem evoked response audiometry. J Laryngol Rhinol Otol. 2007;121(7):639-43. https://doi.org/10.1017/S0022215106004403. PMid:17112395.

[226] Matschke RG, Plath P. Early detection of hearing impairment - a simple method of screening of neonates. HNO. 1985;33:40-4. PMid:3972648.

[227] Matschke RG, Plath P. Early detection of hearing impairment. A simple method of screening of neonates. HNO. 1985;33:40-4. PMid:3972648.

[228] Mattos WM, Cardoso LF, Bissani C, Pinheiro MM, Viveiros CM, Carreirão Filho W. Newborn hearing screening program implantation analysis at a University Hospital. Braz J Otorhinolaryngol. 2009;75(2):237-44. https://doi.org/10.1016/S1808-8694(15)30784-9. PMid:19575110.

[229] Mauk GW, White KR, Mortensen LB, Behrens TR. The effectiveness of screening programs based on high-risk characteristics in early identification of hearing impairment. Ear Hear. 1991;12(5):312-9. https://doi.org/10.1097/00003446-199110000-00003. PMid:1783234.

[230] Maxon AB, White KR, Vohr BR, Behrens TR. Using transient evoked otoacoustic emissions for neonatal hearing screening. Br J Audiol. 1993;27(2):149-53. https://doi.org/10.3109/03005369309077906. PMid:8220282.

[231] Meier S, Narabayashi O, Probst R, Schmuziger N. Comparison of currently available devices designed for newborn hearing screening using automated auditory brainstem and/or otoacoustic emission measurements. Int J Pediatr Otorhinolaryngol. 2004;68(7):927-34. https://doi.org/10.1016/j.ijporl.2004.02.008. PMid:15183584.

[232] Mencher GT. A program for neonatal hearing screening. Audiology. 1974;13(6):495-500. https://doi.org/10.3109/00206097409071713. PMid:4411714.

[233] Meyer ME, Swanepoel DW, le Roux T, van der Linde M. Early detection of infant hearing loss in the private health care sector of South Africa. Int J Pediatr Otorhinolaryngol. 2012;76(5):698-703. https://doi.org/10.1016/j.ijporl.2012.02.023. PMid:22386272.

[234] Mijares Nodarse E, Gaya Vázquez JA, Savio López G, Pérez Abalo MC, Eimil Suárez E, Torres Fortuny A. Diagnostics tests more used in the early detection of hearing losses. Rev Logop Foniatr Audiol. 2006;26:91-100. https://doi.org/10.1016/S0214-4603(06)70107-3.

[235] Molini E, Ricci G, Simoncelli C, Alunni N, Capolynghi B, Giommetti S, et al. Click evoked otoacoustic emissions (EOAES) to screen hearing in neonates. Rev Laryngol Otol Rhinol (Bord). 1996;117:341-3. PMid:9099021.

[236] Molteni G. Early detection of newborn hearing impairment by transiently evoked otoacoustic emissions and auditory evoked potentials. Personal experience in 10,454 children. Otorinolaringologia. 2006;56:93-6.

[237] Monteiro L. Auditory screening of newborns and children. Nascer Crescer. 2004;13:S283-9.

[238] Montovani JC, Fioravanti MP, Tamashiro IA. Avaliaçäo auditiva em recém-nascido e lactente. Rev Paul Pediatr. 1996;14:78-83.

[239] Moradi-Joo E, Barouni M, Vali L, Mahmoudian S. Qualitative Analysis of Newborn Hearing Screening Program in Iran. Med J Islam Repub Iran. 2024;38:146-54. https://doi.org/10.47176/mjiri.38.23. PMid:38783982.

[240] Morgan DE, Canalis RF. Auditory screening of infants. Otolaryngol Clin North Am. 1991;24(2):277-84. https://doi.org/10.1016/S0030-6665(20)31139-7. PMid:1907005.

[241] Mukkun T. The result of universal newborn hearing screening, 4 years of experience in trang. J Med Assoc Thai. 2021;104(1):95-9. https://doi.org/10.35755/jmedassocthai.2021.01.11432.

[242] Murray AD, Javel E, Watson CS. Prognostic validity of auditory brainstem evoked response screening in newborn infants. Am J Otolaryngol. 1985;6(2):120-31. https://doi.org/10.1016/S0196-0709(85)80050-8. PMid:3887960.

[243] Navarro Rivero B, Gonzalez Dîaz E, Marrero Santos L, Martinez Toledano I, Murillo Diaz MJ, Valino Colas MJ. Brainstem auditory evoked responses in children at risk: a prospective study. An Esp Pediatr. 1999;50:357-60. PMid:10356827.

[244] Nennstiel-Ratzel U, Arenz S, Von Kries R, Wildner M, Strutz J. The model project “newborn auditory screening” in the Upper Palatinate. High process and result quality of an interdisciplinary concept. HNO. 2007;55(2):128-34. https://doi.org/10.1007/s00106-006-1383-x. PMid:16528506.

[245] Newton VE, Liu X, Ke X, Xu L, Bamford JM. Evaluation of the use of a questionnaire to detect hearing loss in babies in China. Int J Pediatr Otorhinolaryngol. 1999;48(2):125-9. https://doi.org/10.1016/S0165-5876(99)00016-6. PMid:10375037.

[246] Ng J, Yun HL. Otoacoustic emissions (OAE) in paediatric hearing screening–the Singapore experience. J Singapore Paediatr Soc. 1992;34(1-2):1-5. PMid:1303456.

[247] Ng PK, Hui Y, Lam BCC, Goh WHS, Yeung CY. Feasibility of implementing a universal neonatal hearing screening programme using distortion product otoacoustic emission detection at a university hospital in Hong Kong. Hong Kong Med J. 2004;10(1):6-13. PMid:14967849.

[248] Norton SJ, Gorga MP, Widen JE, Folsom RC, Sininger Y, Cone-Wesson B, et al. Identification of Neonatal Hearing Impairment: evaluation of transient evoked otoacoustic emission, distortion product otoacoustic emission, and auditory brain stem response test performance. Ear Hear. 2000;21(5):508-28. https://doi.org/10.1097/00003446-200010000-00013. PMid:11059707.

[249] Norton SJ, Gorga MP, Widen JE, Vohr BR, Folsom RC, Sininger YS, et al. Identification of Neonatal Hearing Impairment: transient evoked otoacoustic emissions during the perinatal period. Ear Hear. 2000;21(5):425-42. https://doi.org/10.1097/00003446-200010000-00008. PMid:11059702.

[250] Obeidat FS, Alothman N, Alkahtani R, Al-Najjar S, Obeidat M, Ali AY, et al. Evaluation of newborn hearing screening program in Jordan. Front Pediatr. 2024;12:1420678. https://doi.org/10.3389/fped.2024.1420678. PMid:39055617.

[251] Olarte M, Bermúdez Rey MC, Beltran AP, Guerrero D, Suárez-Obando F, López G, et al. Detection of hearing loss in newborns: definition of a screening strategy in Bogotá, Colombia. Int J Pediatr Otorhinolaryngol. 2019;122:76-81. https://doi.org/10.1016/j.ijporl.2019.03.016. PMid:30978473.

[252] Olusanya B. Early detection of hearing impairment in a developing country: what options? Audiology. 2001;40(3):141-7. https://doi.org/10.3109/00206090109073109. PMid:11465296.

[253] Olusanya BO, Ebuehi OM, Somefun AO. Universal infant hearing screening programme in a community with predominant non-hospital births: A three-year experience. J Epidemiol Community Health. 2009;63(6):481-7. https://doi.org/10.1136/jech.2008.082784. PMid:19228683.

[254] Onoda RM, Azevedo MF, dos Santos AMN. Triagem auditiva neonatal: ocorrência de falhas, perdas auditivas e indicadores de riscos. Braz J Otorhinolaryngol. 2011;77(6):775-83. https://doi.org/10.1590/S1808-86942011000600015.

[255] Paat-Ahi G, Sikkut R, Laarmann H. Evaluation of the new born hearing screening programme. Eesti Arst. 2014;93:571-7.

[256] Pastorino G, Sergi P, Mastrangelo M, Ravazzani P, Tognola G, Parazzini M, et al. The Milan Project: a newborn hearing screening programme. Acta Paediatr. 2005;94(4):458-63. https://doi.org/10.1111/j.1651-2227.2005.tb01918.x. PMid:16092461.

[257] Paul AK. Hearing loss in children: need for early detection and intervention. Indian J Pract Pediatr. 2003;5:353-5.

[258] Pedersen L, Møller TR, Wetke R, Ovesen T. Neonatal hearing screening - Transient-evoked oto-acoustic emission compared to automatic auditory brainstem response. Ugeskr Læger. 2008;170:642-6. PMid:18364157.

[259] Pelosi G, Hatzopoulos S, Chierici R, Vigi V, Martini A. Evaluation of a linear TEOAE protocol in hearing screening of neonates: feasibility study. Acta Otorhinolaryngol Ital. 1998;18(4):213-7. PMid:10205919.

[260] Pitathawatchai P, Khaimook W, Kirtsreesakul V. Pilot implementation of newborn hearing screening programme at four hospitals in southern Thailand. Bull World Health Organ. 2019;97(10):663-71. https://doi.org/10.2471/BLT.18.220939. PMid:31656331.

[261] Pitathawatchai P, Wanachottrakul P, Prayuenyong P. Education on loss to follow-up after newborn hearing screening in Thailand: a randomised controlled trial. Int J Audiol. 2025;64(11):1204-10. https://doi.org/10.1080/14992027.2025.2496238. PMid:40266260.

[262] Plinkert PK, Delb W. Universal auditory screening in a German state: a multidisciplinary project. HNO. 2001;49:888-94. https://doi.org/10.1007/s001060170013. PMid:11759240.

[263] Plinkert PK, Delb W. Electronic data processing-assisted organization of interdisciplinary universal hearing screening in Saarland. HNO. 2001;49:888-94. https://doi.org/10.1007/s001060170013.

[264] Plinkert PK, Sesterhenn G, Arold R, Zenner HP. Evaluation of otoacoustic emissions in high-risk infants by using an easy and rapid objective auditory screening method. Eur Arch Oto-Rhino-Laryngol. 1990;247(6):356-60. https://doi.org/10.1007/BF00179006. PMid:2278701.

[265] Poblano A, Chayo I, Ibarra J, Rueda E. Electrophysiological and behavioral methods in early detection of hearing impairment. Arch Med Res. 2000;31(1):75-80. https://doi.org/10.1016/S0188-4409(99)00060-0. PMid:10767484.

[266] Polski B, Szydłowski J, Pucher B, Sroczyński J. The GP’s role in the early detection of hearing loss process, realised by the universal neonatal hearing screenings of infants program. Family Medicine and Primary Care Review. 2014;16:148-9.

[267] Poncet E, Chaperon C. Results of a ten-year screening programme for the diagnosis of deafness and its consequences in children. Early education of the deaf infant. Ann Otolaryngol Chir Cervicofac. 1983;100:543-8. PMid:6670806.

[268] Prieve BA, Stevens F. The New York State universal newborn hearing screening demonstration project: introduction and overview. Ear Hear. 2000;21(2):85-91. https://doi.org/10.1097/00003446-200004000-00003. PMid:10777016.

[269] Prieve BA, Vander Werff KR, Preston JL, Georgantas L. Identification of conductive hearing loss in young infants using tympanometry and wideband reflectance. Ear Hear. 2013;34(2):168-78. https://doi.org/10.1097/AUD.0b013e31826fe611. PMid:23263407.

[270] Prince CB, Miyashiro L, Weirather Y, Heu P. Epidemiology of early hearing loss detection in Hawaii. Pediatrics. 2003;111(5 Pt 2, Suppl 1):1202-6. https://doi.org/10.1542/peds.111.S1.1202. PMid:12728139.

[271] Ptok M. Early diagnosis of hearing impairment in children. Z Arztl Fortbild Qualitatssich. 2004;98(4):265-70. PMid:15295927.

[272] Radü HJ. Comparison of different audiometric procedures for infants less than 1 year old. HNO. 1985;33(6):271-4. PMid:4030410.

[273] Raghuwanshi SK, Gargava A, Kulkarani V, Kumar A. Role of otoacoustic emission test in neonatal screening at Tertiary Center. Indian J Otolaryngol Head Neck Surg. 2019;71(Suppl 2):1535-7. https://doi.org/10.1007/s12070-019-01606-0. PMid:31750212.

[274] Appanaitis I, Rahimian R. Early hearing detection and intervention in the Republic of Palau: program successes and challenges. J Investig Med. 2018;66(1):A106-7. https://doi.org/10.1136/jim-2017-000663.100.

[275] Reis FMFS, Gonçalves CGO, Conto J, Iantas M, Lüders D, Marques J. Hearing assessment of neonates at risk for hearing loss at a hearing health high complexity service: an electrophysiological assessment. Int Arch Otorhinolaryngol. 2019;23(2):157-64. https://doi.org/10.1055/s-0038-1648217. PMid:30956699.

[276] Ren B, Zhang X, Zhang Q, Gan W, Zhang Y, Guo B, et al. Associational study of neonatal hearing screening results and common metabolic disorders. Int J Pediatr Otorhinolaryngol. 2025;196:112489. https://doi.org/10.1016/j.ijporl.2025.112489. PMid:40690833.

[277] Richard C, Shakhtour L, Gallo N, Smith R, MacDonald CB, Gentry R, et al. Audiological outcomes of cytomegalovirus saliva PCR-Positive Newborns in support of universal screening. Otolaryngol Head Neck Surg. 2025;173(5):1245-53. https://doi.org/10.1002/ohn.1332. PMid:40504068.

[278] Rodrigues RP. Avaliação da implantação do Programa de Triagem Auditiva Neonatal em maternidades públicas brasileiras [dissertação]. Rio de Janeiro: Fundação Oswaldo Cruz, Instituto Nacional de Saúde da Mulher, da Criança e do Adolescente Fernandes Figueira; 2020 [citado em 2026 maio 18]. Disponível em: https://acervos.icict.fiocruz.br/iff/mestrado_bibsmc/renata_rodrigues_iff_mest_2020.pdf

[279] Roman S, Mondain M, Triglia JM, Uziel A. Auditory screening in neonates: evoked oto-acoustic emissions or acoustic distortion product? Rev Laryngol Otol Rhinol (Bord). 2001;122(3):155-8. PMid:11799854.

[280] Ruiz De La Cuesta F, Juste Ruiz M, Cortés Castell E. Results of applying for 13 years the universal newborn hearing screening protocol and study of cases that do not pass the screening. Acta Pediatr Esp. 2018;76:77-82.

[281] Saitoh Y, Hazama M, Sakoda T, Hamada H, Ikeda H, Seno S, et al. Outcome of neonatal screening for hearing loss in neonatal intensive care unit and well-born nursery infants. Nippon Jibiinkoka Gakkai Kaiho. 2002;105(12):1205-11. https://doi.org/10.3950/jibiinkoka.105.1205. PMid:12607282.

[282] Saki N, Bayat A, Hoseinabadi R, Nikakhlagh S, Karimi M, Dashti R. Universal newborn hearing screening in southwestern Iran. Int J Pediatr Otorhinolaryngol. 2017;97:89-92. https://doi.org/10.1016/j.ijporl.2017.03.038. PMid:28483258.

[283] Santos-Ceballos JC, Pérez-Blanco JG, Pantoja-Gómez Y, Martín-González F, Torres-Fortuny A, Eimil-Suarez E, et al. Implementation of the NEURONIC INFANTIX Newborn Hearing Screening System. IFMBE Proc. 2020;75:453-9. https://doi.org/10.1007/978-3-030-30648-9_59.

[284] Sarno CN, Clemis JD. A workable approach to the identification of neonatal hearing impairment. Laryngoscope. 1980;90(8 Pt 1):1313-20. https://doi.org/10.1288/00005537-198008000-00008. PMid:7401831.

[285] Sass-Lehrer M. Early detection of hearing loss: maintaining a family-centered perspective. Semin Hear. 2004;25(4):295-307. https://doi.org/10.1055/s-2004-836132.

[286] Sassada MMY, Ceccon MEJ, de Navarro JM, Vaz FAC. Avaliação auditiva de recém-nascidos gravemente enfermos através do método de emissões otoacústicas evocadas transientes (EOAT) e audiometria de tronco cerebral(BERA). Pediatria (Säo Paulo). 2005;27:154-62.

[287] Satish HS, Anil Kumar R, Viswanatha B. Screening of Newborn Hearing at a Tertiary Care Hospital in South India. Indian J Otolaryngol Head Neck Surg. 2019;71(Suppl 2):1383-90. https://doi.org/10.1007/s12070-018-1454-9. PMid:31750182.

[288] Schade G. Early detection of hearing disorders. Laryngorhinootologie. 2008;87(Suppl 1):S21-31. https://doi.org/10.1055/s-2007-995550. PMid:18373306.

[289] Schauseil-Zipf U, Von Wedel H. Hearing screening by means of acoustic evoked brain stem potentials in newborns and infants. Klin Padiatr. 1988;200(4):324-9. https://doi.org/10.1055/s-2008-1033729. PMid:3172672.

[290] Schmidt P, Leveque M, Danvin JB, Leroux B, Chays A. Systematic hearing screening for newborns in the Champagne-Ardennes region: 32,500 births in 2 years of experience. Ann Otolaryngol Chir Cervicofac. 2007;124(4):157-65. https://doi.org/10.1016/j.aorl.2006.10.004. PMid:17669353.

[291] Schönweiler R, Tioutou E, Tolloczko R, Pankau R, Ptok M. Hearing screening with automatic evaluation of TEOAE and a new method of automatic evaluation of early auditory evoked potentials. Optimization and field trial. HNO. 2002;50:649-56. https://doi.org/10.1007/s00106-002-0636-6.

[292] Sedano MC, San Martín UA, Rahal EM. Realidad nacional de los programas de detección auditiva temprana con miras a la cobertura universal. Rev Otorrinolaringol Cir Cabeza Cuello. 2018;78(1):9-14. https://doi.org/10.4067/s0717-75262018000100009.

[293] Seguya A, Bajunirwe F, Kakande E, Nakku D. Feasibility of establishing an infant hearing screening program and measuring hearing loss among infants at a regional referral hospital in south western Uganda. PLoS One. 2021;16(6):e0253305. https://doi.org/10.1371/journal.pone.0253305. PMid:34138954.

[294] Sente M, Aleksov-Hatvan G. Early detection of hearing impairment in children. Srp Arh Celok Lek. 2006;134(9-10):448-52. PMid:17252916.

[295] Shang Y, Hao W, Gao Z, Xu C, Ru Y, Ni D. An effective compromise between cost and referral rate: a sequential hearing screening protocol using TEOAEs and AABRs for healthy newborns. Int J Pediatr Otorhinolaryngol. 2016;91:141-5. https://doi.org/10.1016/j.ijporl.2016.10.025. PMid:27863628.

[296] Sharma R, Gu Y, Sinha K, Ching TYC, Marnane V, Gold L, et al. An Economic Evaluation of Australia’s Newborn Hearing Screening Program: A Within-Study Cost-Effectiveness Analysis. Ear Hear. 2022;43(3):972-83. https://doi.org/10.1097/AUD.0000000000001153. PMid:34772837.

[297] Shehata-Dieler WE, Dieler R, Keim R, Finkenzeller P, Dietl J, Helms J. Universal hearing screening in newborns using the CRESCENDO newborn hearing screener. Laryngorhinootologie. 2000;79:69-76. https://doi.org/10.1055/s-2000-8792. PMid:10738712.

[298] Shi Y, Zhang N, Du N, Zheng T, Yu Y, Li Y. Genetic and audiological determinants of hearing loss in high-risk neonates. Braz J Otorhinolaryngol. 2025;91(2):101541. https://doi.org/10.1016/j.bjorl.2024.101541. PMid:39754783.

[299] Shirane M, Ganaha A, Nakashima T, Shimoara S, Yasunaga T, Ichihara S, et al. Comprehensive hearing care network for early identification and intervention in children with congenital and late-onset/acquired hearing loss: 8 years’ experience in Miyazaki. Int J Pediatr Otorhinolaryngol. 2020;131:109881. https://doi.org/10.1016/j.ijporl.2020.109881. PMid:31978747.

[300] Shulman S, Besculides M, Saltzman A, Ireys H, White KR, Forsman I. Evaluation of the universal newborn hearing screening and intervention program. Pediatrics. 2010;126(Suppl 1):S19-27. https://doi.org/10.1542/peds.2010-0354F. PMid:20679316.

[301] Solanellas Soler J. Hearing loss: early hearing detection and intervention. Pediatria Integral. 2009;13:457-67.

[302] Sun JH, Li J, Huang P, Bu J, Xu ZM, Li J, et al. Early detection of hearing impairment in high-risk infants of NICU. Zhonghua Er Ke Za Zhi. 2003;41(5):357-9. PMid:14751056.

[303] Sun X, Shen X, Zakus D, Lv J, Xu Z, Wu H, et al. Development of an effective public health screening program to assess hearing disabilities among newborns in Shanghai: a prospective cohort study. World Health Popul. 2009;11(1):14-23. PMid:20039591.

[304] Sun X, Xi Z, Zhang J, Liu B, Xing X, Huang X, et al. Combined hearing and deafness gene mutation screening of 11 046 Chinese newborns. Zhonghua Yi Xue Yi Chuan Xue Za Zhi. 2015;32(6):766-70. https://doi.org/10.3760/cma.j.issn.1003-9406.2015.06.002. PMid:26663044.

[305] Sun XY, Ding J, Zhang Y, Xu Y. Analysis of hearing screening results of 268 187 newborns in Jiaxing from 2013 to 2017. Zhongguo Ertong Baojian Zazhi. 2019;27:453-6. https://doi.org/10.11852/zgetbjzz2018-0999.

[306] Swanepoel DCD, Delport SD, Swart JG. Universal newborn hearing screening in South Africa - A first-world dream? S Afr Med J. 2004;94(8):634-5. PMid:15352584.

[307] Szyfter W, Greczka G, Dąbrowski P, Wróbel M. The report on the Universal Neonatal Hearing Screening Program in Poland between 2003 and 2015. Otolaryngol Pol. 2016;70(2):1-5. https://doi.org/10.5604/00306657.1199346. PMid:27386827.

[308] Szyfter W, Wróbel M, Radziszewska-Konopka M, Szyfter-Harris J, Karlik M. Polish Universal Neonatal Hearing Screening Program-4-year experience (2003-2006). Int J Pediatr Otorhinolaryngol. 2008;72(12):1783-7. https://doi.org/10.1016/j.ijporl.2008.08.015. PMid:18922586.

[309] Tanon-Anoh MJ, Sanogo-Gone D, Kouassi KB. Newborn hearing screening in a developing country: results of a pilot study in Abidjan, Côte d’ivoire. Int J Pediatr Otorhinolaryngol. 2010;74(2):188-91. https://doi.org/10.1016/j.ijporl.2009.11.008. PMid:19963282.

[310] Tham EH, Lee LY, Joseph R. Evaluation of newborn screening practices among paediatricians practising in the private sector in Singapore. Ann Acad Med Singap. 2010;39:S168.

[311] Todd NW. Universal newborn hearing screening follow-up in two Georgia populations: newborn, mother and system correlates. Int J Pediatr Otorhinolaryngol. 2006;70(5):807-15. https://doi.org/10.1016/j.ijporl.2005.09.019. PMid:16246432.

[312] Trinidad Ruiz G, Pantoja Hernández CG, Trinidad Ramos G, Serrano Berrocal MA, Pardo Romero G, González Palomino A, et al. Controlling retests in a universal hearing screening program. Acta Otorrinolaringologica Espanola. 2005;56(3):96-101. https://doi.org/10.1016/S0001-6519(05)78580-0. PMid:15819515.

[313] Tufatulin GS, Lalayants MR, Artyushkin SA, Vikhnina SM, Garbaruk ES, Dvoryanchikov VV, et al. Clinical protocol: audiological assessment of infants in Russian Federation. Part II. Vestn Otorinolaringol 2023;88(6):81-90. https://doi.org/10.17116/otorino20238806181.

[314] Turan Z, Bas N. Evaluation of the effectiveness of newborn hearing screening program: a center in Turkey. Int J Early Child Spec Educ. 2019;11:141-53. https://doi.org/10.20489/intjecse.670470.

[315] Tzanakakis MG, Chimona TS, Apazidou E, Giannakopoulou C, Velegrakis GA, Papadakis CE. Transitory evoked otoacoustic emission (TEOAE) and distortion product otoacoustic emission (DPOAE) outcomes from a three-stage newborn hearing screening protocol. Hippokratia. 2016;20(2):104-9. PMid:28416905.

[316] Ulusoy S, Ugras H, Cingi C, Yilmaz HB, Muluk NB. The results of national newborn hearing screening (NNHS) data of 11,575 newborns from west part of Turkey. Eur Rev Med Pharmacol Sci. 2014;18(20):2995-3003. PMid:25392094.

[317] Ulusoy S, Uʇraş H, Cingi C, Yilmaz HB. Analysis of hearing screening test findings of 11,575 newborns in Turkey. OHNS. 2013;149(S2):240-1. https://doi.org/10.1177/0194599813496044a299.

[318] Uppenkamp S, Jäkel M, Talartschick B, Büschel J, Kollmeier B. Evoked otoacoustic emissions as a screening test for hearing evaluation in newborn and premature infants?. Laryngorhinootologie 1992;71(10):525-9. https://doi.org/ 10.1055/s-2007-997347.

[319] Valencia DM, Rimell FL, Friedman BJ, Oblander MR, Helmbrecht J. Cochlear implantation in infants less than 12 months of age. Int J Pediatr Otorhinolaryngol. 2008;72(6):767-73. https://doi.org/10.1016/j.ijporl.2008.02.009. PMid:18403026.

[320] Van Straaten HLM, Groote ME, Oudesluys-Murphy AM. Evaluation of an automated auditory brainstem response infant hearing screening method in at risk neonates. Eur J Pediatr. 1996;155(8):702-5. https://doi.org/10.1007/BF01957157. PMid:8839729.

[321] Van Straaten HL, Tibosch CH, Dorrepaal C, Dekker FW, Kok JH. Efficacy of automated auditory brainstem response hearing screening in very preterm newborns. J Pediatr. 2001;138(5):674-8. https://doi.org/10.1067/mpd.2001.112646. PMid:11343042.

[322] Vernier LS, Da Rocha BSC, Reppold CT, Zanini CFC, Levandowski DC, De Carvalho Paniz T. Which is the best time to perform the newborn hearing screening? Preliminary results of a study conducted in a public hospital of Porto Alegre. Int Arch Otorhinolaryngol. 2014;18:a2297. https://doi.org/10.1055/s-0034-1389006.

[323] Verrue N, Smets K. Possibilities and pitfalls of early neonatal hearing screening with automated auditory brainstem response measurements in a neonatal intensive care unit. Tijdschr Geneeskd. 2012;68:497-504. https://doi.org/10.2143/TVG.68.10.2001188.

[324] Vila C, Demestre X, Sagrera X, Sala P, Raspall F. Newborn hearing screening. Pediatria Catalana. 2004;64:20-4.

[325] Voegeli R. Early detection of hearing impairment in childhood. Therapeutische Umschau. 1979;36(9):788-91. PMid:505326.

[326] Vohr B, Simon P, McDermott C, Kurtzer-White E, Johnson MJ, Topol D. Early hearing screening, detection and intervention (EHDI) in Rhode Island. Med Health R I. 2002;85(12):369-72. PMid:12593355.

[327] Vos B, Lagasse R, Levêque A. The organisation of universal newborn hearing screening in the Wallonia-Brussels Federation. B-ENT. 2013;(Suppl 21):9-15. PMid:24383218.

[328] Vranješ D, Spremo S, Travar D, Aleksić A, Novaković Z, Stevandić N, et al. The role and importance of screening procedures in early diagnostics of hearing impairment. Medicinski Casopis. 2012;46(2):71-6. https://doi.org/10.5937/mckg46-1265.

[329] Wang DY, Xing GQ, Liu ZY. Transiently evoked otoacoustic emissions for universal newborn hearing screening. Zhongguo Linchuang Kangfu. 2002;6:2210-1.

[330] Wang J, Chen W, Wang J, Li J, Zhao Y, Chen P. Evaluation of the effect of combined otoacoustic emission and automatic auditory EEG response in neonatal hearing screening. Minerva Pediatr (Torino). 2022;74(2):239-41. https://doi.org/10.23736/S2724-5276.21.06576-9. PMid:34530588.

[331] Watkin PM. Controlling the quality of universal neonatal hearing screens. Public Health. 1999;113(4):171-6. https://doi.org/10.1016/S0033-3506(99)00148-1. PMid:10483078.

[332] Weichbold V, Nekahm-Heis D, Welzl-Müller K. Evaluation of the Austrian Newborn Hearing Screening Program. Wien Klin Wochenschr. 2005;117(18):641-6. https://doi.org/10.1007/s00508-005-0414-z. PMid:16416347.

[333] Welzl-Müller K, Stephan K. Examples of implemented neonatal hearing screening programs in Austria. Scand Audiol Suppl. 2001;30(52):7-9. https://doi.org/10.1080/010503901300006912. PMid:11318488.

[334] Widen JE, Folsom RC, Cone-Wesson B, Carty L, Dunnell JJ, Koebsell K, et al. Identification of Neonatal Hearing Impairment: hearing status at 8 to 12 months corrected age using a visual reinforcement audiometry protocol. Ear Hear. 2000;21(5):471-87. https://doi.org/10.1097/00003446-200010000-00011. PMid:11059705.

[335] Wong YA, Mazlan R, Abdul Wahab NA, Ja’afar R, Huda Bani N, Abdullah NA. Quality measures of a multicentre universal newborn hearing screening program in Malaysia. J Med Screen. 2021;28(3):238-43. https://doi.org/10.1177/0969141320973060. PMid:33202173.

[336] Wu JL, Huang CC, Kao CC. A hearing screening in very low birth weight preterm infants by auditory brainstem response. J Otolaryngol Soc Repub China. 1998;33:212-8.

[337] Xu J, Chen S, Zheng Z, Xu M, Shen A. [Follow-up examination for newborns and infants who failed hearing screening]. Lin Chuang Er Bi Yan Hou Tou Jing Wai Ke Za Zhi. 2006;20:446-8.

[338] Xu Z, Li J. Performance of two hearing screening protocols in the NICU. B-ENT. 2005;1(1):11-5. PMid:15999670.

[339] Xu ZM, Cheng WX, Yang XL. Performance of two hearing screening protocols in NICU in Shanghai. Int J Pediatr Otorhinolaryngol. 2011;75(10):1225-9. https://doi.org/10.1016/j.ijporl.2011.07.004. PMid:21802153.

[340] Xu Z, Li J. Performance of two hearing screening protocols in the NICU. B-ENT. 2005;1(1):11-5. PMid:15999670.

[341] Xu ZM, Li J, Hu TZ, Sun JH, Shen XM. Sensitivity of distortion product otoacoustic emissions and auditory brain-stem response in neonatal hearing screening, a comparative study. Zhonghua Yi Xue Za Zhi. 2003;83(4):278-80. PMid:12812641.

[342] Yao GD, Li SX, Chen DL, Feng HQ, Zhao SB, Liu YJ, et al. Combination of hearing screening and genetic screening for deafness-susceptibility genes in newborns. Exp Ther Med. 2013;7(1):218-22. https://doi.org/10.3892/etm.2013.1406. PMid:24348793.

[343] Yigit Ö, Batuk M, Çinar B, Yildirim M, Yarali M, Sennaroglu G. Auditory brainstem response measurements in newborns: which electrode placement is better? Turk Arch Otorhinolaryngol. 2020;58(2):112-7. https://doi.org/10.5152/tao.2020.5065. PMid:32783038.

[344] Yılmazer R, Yazıcı MZ, Erdim İ, Kaya HK, Özcan Dalbudak Ş, Kayhan TF. Follow-up results of newborns after hearing screening at a training and Research Hospital in Turkey. J Int Adv Otol. 2016;12(1):55-60. https://doi.org/10.5152/iao.2015.1736. PMid:27340984.

[345] Yoshinaga-Itano C. Universal newborn hearing screening programs and developmental outcomes. Audiol Med. 2003;1(3):199-206. https://doi.org/10.1080/16513860310002031.

[346] Yoshinaga-Itano C, Apuzzo MRL. Identification of hearing loss after age 18 months is not early enough. Am Ann Deaf. 1998;143(5):380-7. https://doi.org/10.1353/aad.2012.0151. PMid:9893323.

[347] Yoshinaga-Itano C, Gravel JS. The evidence for universal newborn hearing screening. Am J Audiol. 2001;10(2):62-4. https://doi.org/10.1044/1059-0889(2001/013). PMid:11808721.

[348] Yousefi J, Ajalloueyan M, Amirsalari S, Fard MH. The specificity and sensitivity of transient otoacustic emission in neonatal hearing screening compared with diagnostic test of auditory brain stem response in Tehran hospitals. Iran J Pediatr. 2013;23(2):199-204. PMid:23724183.

[349] Yousefi J, Ajalloueyan M, Amirsalari S, Hassanali Fard M. The specificity and sensitivity of transient otoacustic emission in neonatal hearing screening compared with diagnostic test of auditory brain stem response in tehran hospitals. Iran J Pediatr. 2013;23(2):199-204. PMid:23724183.

[350] Zeng X, Liu Z, Wang J, Zeng X. Combined hearing screening and genetic screening of deafness among Hakka newborns in China. Int J Pediatr Otorhinolaryngol. 2020;136:110120. https://doi.org/10.1016/j.ijporl.2020.110120. PMid:32574949.

[351] Zhang Y, Li G, Zheng Y. Early diagnosis and intervention in 0-9 months old infants with hearing loss. Lin Chuang Er Bi Yan Hou Tou Jing Wai Ke Za Zhi. 2014;28(22):1748-51. PMid:25752104.

[352] Zhang Z, Liu Y, Xie LS, Jia DQ, Gao PM, Luo RZ. Establishment and procedure of universal newborn hearing screening. Zhongguo Linchuang Kangfu. 2004;8:2304-6.

[353] Zhao PJ, Xu ZM, Wu SH, Jin CH, Yu H, Shen P, et al. Hearing screening for high-risk newborns. Zhonghua Yi Xue Za Zhi. 2003;83(4):285-8. PMid:12812643.

[354] Zwicker E, Schorn K. Delayed evoked otoacoustic emissions - an ideal screening-test for excluding hearing impairment in infants. Audiology. 1990;29(5):241-51. https://doi.org/10.3109/00206099009072855. PMid:2275639.

[355] Zych M, Greczka G, Dąbrowski P, Wróbel M, Szyfter-Harris J, Szyfter W. The report of the polish universal neonatal hearing screening program in 2016. Otolaryngol Pol. 2018;72(1):1-4. https://doi.org/10.5604/01.3001.0011.5913. PMid:29513258.

## References

[1] Yoshinaga-Itano C, Sedey AL, Wiggin M, Chung W (2017). Early hearing detection and vocabulary of children with hearing loss. Pediatrics.

[2] Dolphine MDLRG, Lima MCMP, Colella-Santos MF (2023). Utilização do Potencial Auditivo de Estado Estável em lactentes com baixo risco para perda auditiva. Distúrb Comun.

[3] Harris AB, Seeliger E, Hess C, Sedey AL, Kristensen K, Lee Y (2022). Early Identification of Hearing Loss and Language Development at 32 Months of Age. J Otorhinolaryngol Hear Balanc Med.

[4] Fonseca LR, Almeida TFD, Ferreira ELO, Alves NRO, Carvalho LE, Medina C (2024). Triagem auditiva neonatal: construção e validação de material educativo para gestantes e puérperas. CLCS.

[5] Tu LJ, Benchetrit L, Glovsky CK, Cohen MS (2023). Impact of COVID-19 on diagnosis and management of newborn hearing loss. Int J Pediatr Otorhinolaryngol.

[6] Sequi-Canet JM, Brines-Solanes J (2021). Keypoints to Successful Newborn Hearing Screening. Thirty Years of Experience and Innovations. Healthcare (Basel).

[7] Brasil (2002). Manual de normas tecnicas e rotinas operacionais do programa nacional de triagem neonatal.

[8] Mackey AR, Bussé AML, Del Vecchio V, Mäki-Torkko E, Uhlén IM (2022). Protocol and programme factors associated with referral and loss to follow-up from newborn hearing screening: a systematic review. BMC Pediatr.

[9] Rajanbabu K, Joshi BD, Ramkumar V, Kuper H, Vaidyanath R (2024). Early Hearing Detection and Intervention programmes for neonates, infants and children in non-Asian low-income and middle-income countries: a systematic review. BMJ Paediatr Open.

[10] WHO: World Health Organization (2016). Childhood hearing loss: strategies for prevention and care..

[11] Findlen UM, Davenport CA, Cadieux J, Gehred A, Frush Holt R, Vaughn LM (2023). Barriers to and Facilitators of Early Hearing Detection and Intervention in the United States: A Systematic Review. Ear Hear.

[12] WHO: World Health Organization (2021). World report on hearing.

[13] Guo Z, Ji W, Song P, Zhao J, Yan M, Zou X (2024). Global, regional, and national burden of hearing loss in children and adolescents, 1990–2021: a systematic analysis from the Global Burden of Disease Study 2021. BMC Public Health.

[14] Besen E, Paiva KM, Cigana LB, Machado MJ, Samelli AG, Haas P (2023). Prevalence of Congenital Infections in Newborns and Universal Neonatal Hearing Screening in Santa Catarina, Brazil. Audiol Res.

[15] Jafarzadeh S, Khajedaluee M, Khajedaluee AR, Khakzadi M, Esmailzadeh M, Firozbakht M (2023). Early Hearing Detection and Intervention Results in Northeastern of Iran from 2005 to 2019: A Repeated Cross-Sectional Study. Int J Prev Med.

[16] Nelson HJ, Munns A, Angus B, Arbuckle E, Burns SK (2025). Facilitators and barriers of accessing community health services for children in the early years: an Australian qualitative study. J Pediatr Nurs.

[17] Jenks CM, DeSell M, Walsh J (2022). Delays in infant hearing detection and intervention during the COVID‐19 pandemic: commentary. Otolaryngol Head Neck Surg.

[18] Gomes VCA, Badarane EBL, Seto IIC, Yamaguchi CT, Ferreira DB, Umbelino AM (2021). Avaliação das queixas auditivas e das otoemissões acústicas em funcionários do Complexo Hospitalar Universitário da Universidade Federal do Pará com COVID-19 / Evaluation of hearing complaints and otoacoustic emissions in employees of the University Hospital Complex of the Universidade Federal do Pará with COVID-19. BJHR.

[19] Nascimento GB, Kessler TM, Souza APRD, Costa I, Moraes ABD (2020). Indicadores de risco para a deficiência auditiva e aquisição da linguagem e sua relação com variáveis socioeconômicas, demográficas e obstétricas em bebês pré-termo e a termo. CoDAS.

[20] Aromataris E, Lockwood C, Porritt K, Pilla B, Jordan Z (2024). JBI Manual for Evidence Synthesis..

[21] Peters MDJ, Marnie C, Tricco AC, Pollock D, Munn Z, Alexander L (2020). Updated methodological guidance for the conduct of scoping reviews. JBI Evid Synth.

[22] Open Science Framework (2024). A survey of strategies for the early diagnosis of hearing loss in infants: a scoping review.

[23] Rayyan® (2026). https://www.rayyan.ai/.

[24] Page MJ, McKenzie JE, Bossuyt PM, Boutron I, Hoffmann TC, Mulrow CD (2021). The PRISMA 2020 statement: an updated guideline for reporting systematic reviews. BMJ.

[25] Tricco AC, Lillie E, Zarin W, O’Brien KK, Colquhoun H, Levac D (2018). PRISMA Extension for Scoping Reviews (PRISMA-ScR): checklist and explanation. Ann Intern Med.

[26] Webb HD, Stevens JC (1991). Auditory screening in high risk neonates: selection of a test protocol. Clin Phys Physiol Meas.

[27] Tudehope D, Smyth V, Scott J, Rogers Y (1992). Audiological evaluation of very low birthweight infants. J Paediatr Child Health.

[28] Morlet T, Ferber-Viart C, Putet G, Sevin F, Duclaux R (1998). Auditory screening in high-risk pre-term and full-term neonates using transient evoked otoacoustic emissions and brainstem auditory evoked potentials. Int J Pediatr Otorhinolaryngol.

[29] Aidan D, Avan P, Bonfils P (1999). Auditory screening in neonates by means of transient evoked otoacoustic emissions: a report of 2,842 recordings. Ann Otol Rhinol Laryngol.

[30] Watkin PM, Baldwin M (1999). Confirmation of deafness in infancy. Arch Dis Child.

[31] Prieve B, Dalzell L, Berg A, Bradley M, Cacace A, Campbell D (2000). The New York State universal newborn hearing screening demonstration project: outpatient outcome measures. Ear Hear.

[32] Shehata-Dieler WE, Dieler R, Keim R, Finkenzeller P, Dietl J, Helms J (2000). Universal hearing screening of newborn infants with the BERA-phone. Laryngorhinootologie.

[33] Stewart DL, Mehl A, Hall JW, Thomson V, Carroll M, Hamlett J (2000). Universal newborn hearing screening with automated auditory brainstem response: a multisite investigation. J Perinatol.

[34] Baumann U, Schorn K (2001). Früherkennung kindlicher Hörschäden. HNO.

[35] Owen M, Webb M, Evans K (2001). Community based universal neonatal hearing screening by health visitors using otoacoustic emissions. Arch Dis Child Fetal Neonatal Ed.

[36] Lin HC, Shu MT, Chang KC, Bruna SM (2002). A universal newborn hearing screening program in Taiwan. Int J Pediatr Otorhinolaryngol.

[37] Iwasaki S, Hayashi Y, Seki A, Nagura M, Hashimoto Y, Oshima G (2003). A model of two-stage newborn hearing screening with automated auditory brainstem response. Int J Pediatr Otorhinolaryngol.

[38] Rouev P, Mumdzhiev H, Spiridonova J, Dimov P (2004). Universal newborn hearing screening program in Bulgaria. Int J Pediatr Otorhinolaryngol.

[39] White KR, Vohr BR, Meyer S, Widen JE, Johnson JL, Gravel JS (2005). A multisite study to examine the efficacy of the otoacoustic emission/automated auditory brainstem response newborn hearing screening protocol: research design and results of the study. Am J Audiol.

[40] Widen JE, Johnson JL, White KR, Gravel JS, Vohr BR, James M (2005). A multisite study to examine the efficacy of the otoacoustic emission/automated auditory brainstem response newborn hearing screening protocol: results of visual reinforcement audiometry. Am J Audiol.

[41] Yee-Arellano HM, Leal-Garza F, Pauli-Müller K (2006). Universal newborn hearing screening in Mexico: results of the first 2 years. Int J Pediatr Otorhinolaryngol.

[42] De Capua B, Costantini D, Martufi C, Latini G, Gentile M, De Felice C (2007). Universal neonatal hearing screening: the Siena (Italy) experience on 19,700 newborns. Early Hum Dev.

[43] Tatli MM, Serbetcioglu MB, Duman N, Kumral A, Kirkim G, Ogun B (2007). Feasibility of neonatal hearing screening program with two-stage transient otoacoustic emissions in Turkey. Pediatr Int.

[44] Olusanya BO, Wirz SL, Luxon LM (2008). Hospital-based universal newborn hearing screening for early detection of permanent congenital hearing loss in Lagos, Nigeria. Int J Pediatr Otorhinolaryngol.

[45] Dantas MBS, Anjos CAL, Camboim ED, Pimentel MCR (2009). Anjos CAL dos, Camboim ED, Pimentel M de CR. Resultados de um programa de triagem auditiva neonatal em Maceió. Rev Bras Otorrinolaringol.

[46] Ohl C, Dornier L, Czajka C, Chobaut JC, Tavernier L (2009). Newborn hearing screening on infants at risk. Int J Pediatr Otorhinolaryngol.

[47] Geal-Dor M, Adelman C, Levi H, Zentner G, Stein-Zamir C (2010). Comparison of two hearing screening programs in the same population: oto-acoustic emissions (OAE) screening in newborns and behavioral screening when infants. Int J Pediatr Otorhinolaryngol.

[48] Tasci Y, Muderris I, Erkaya S, Altinbas S, Yucel H, Haberal A (2010). Newborn hearing screening programme outcomes in a research hospital from Turkey. Child Care Health Dev.

[49] Yu JKY, Ng IHY, Kam ACS, Wong TKC, Wong ECM, Tong MCF (2010). The universal neonatal hearing screening (UNHS) program in Hong Kong: the outcome of a combined otoacoustic emissions and automated auditory brainstem response screening protocol. Hong Kong J Paediatr.

[50] Daniel LM, Lim SB (2012). The hearing screening programme for infants in KK Women’s and Children’s Hospital - Its development and role in reducing the burden of hearing impairment in Singapore. Proceedings of Singapore Healthcare.

[51] Friderichs N, Swanepoel D, Hall JW (2012). Efficacy of a community-based infant hearing screening program utilizing existing clinic personnel in Western Cape, South Africa. Int J Pediatr Otorhinolaryngol.

[52] Lim HW, Kim EAR, Chung JW (2012). Audiological follow-up results after newborn hearing screening program. Clin Exp Otorhinolaryngol.

[53] Martines F, Salvago P, Bentivegna D, Bartolone A, Dispenza F, Martines E (2012). Audiologic profile of infants at risk: experience of a Western Sicily tertiary care centre. Int J Pediatr Otorhinolaryngol.

[54] Zhang Z, Ding W, Liu X, Xu B, Du W, Nan S (2012). Auditory screening concurrent deafness predisposing genes screening in 10,043 neonates in Gansu province, China. Int J Pediatr Otorhinolaryngol.

[55] Arslan S, Işık AÜ, İmamoğlu M, Topbaş M, Aslan Y, Ural A (2013). Universal newborn hearing screening; automated transient evoked otoacoustic emissions. B-ENT.

[56] Borkoski Barreiro SA, Falcón González JC, Bueno Yanes J, Pérez Bermúdez JL, López Cano Z, Ramos Macías Á (2013). Resultados de un programa de detección precoz de la hipoacusia neonatal. Acta Otorrinolaringol Esp.

[57] Hsu HC, Lee FP, Huang HM (2013). Results of a 1-year government-funded newborn hearing screening program in Taiwan. Laryngoscope.

[58] Kuki S, Chadha S, Dhingra S, Gulati A (2013). The role of current audiological tests in the early diagnosis of hearing impairment in infant. Indian J Otolaryngol Head Neck Surg.

[59] Mishra G, Sharma Y, Mehta K, Patel G (2013). Efficacy of Distortion Product Oto-Acoustic Emission (OAE)/Auditory Brainstem Evoked Response (ABR) Protocols in Universal Neonatal Hearing Screening and Detecting Hearing Loss in Children <2 Years of Age. Indian J Otolaryngol Head Neck Surg.

[60] Qi B, Cheng X, En H, Liu B, Peng S, Zhen Y (2013). Assessment of the feasibility and coverage of a modified universal hearing screening protocol for use with newborn babies of migrant workers in Beijing. BMC Pediatr.

[61] Amini E, Kasheh Farahani Z, Rafiee Samani M, Hamedi H, Zamani A, Karimi Yazdi A (2014). Assessment of Hearing Loss by OAE in Asphyxiated Newborns. Iran Red Crescent Med J.

[62] Celik IH, Canpolat FE, Demirel G, Eras Z, Gencay Sungur V, Sarier B (2014). Zekai Tahir Burak Women’s Health Education and Research Hospital newborn hearing screening results and assessment of the patients. Turk Pediatri Ars.

[63] Huang Y, Liang R, Wen C, Gan J, Lv Q, Lan X (2014). [Analysis in 13 315 newborns hearing screening]. Lin Chuang Er Bi Yan Hou Tou Jing Wai Ke Za Zhi.

[64] Canet JMS, Langa MJS, del Castillo JIC (2016). Results from ten years newborn hearing screening in a secondary hospital. An Pediatr (Barc).

[65] Chrobok V, Dršata J, Janouch M, Komínek P, Kokštein Z, Malý J (2019). Updating the nationwide methodology for hearing screening of newborns in the Czech Republic. Cas Lek Cesk.

[66] Wasser J, Ari-Even Roth D, Herzberg O, Lerner-Geva L, Rubin L (2019). Assessing and monitoring the impact of the national newborn hearing screening program in Israel. Isr J Health Policy Res.

[67] Dey SK, Islam S, Jahan I, Shabuj KH, Begum S, Chisti MJ (2020). Association of hyperbilirubinemia requiring phototherapy or exchange transfusion with hearing impairment among admitted term and late preterm newborn in a NICU. Mymensingh Med J.

[68] McInerney M, Scheperle R, Zeitlin W, Bodkin K, Uhl B (2020). Adherence to follow-up recommendations for babies at risk for pediatric hearing loss. Int J Pediatr Otorhinolaryngol.

[69] Nishad A, Gangadhara Somayaji KS, Mithun HK, Sequeira N (2020). A study of incidence of hearing loss in newborn, designing a protocol and methodology to detect the same in a tertiary health-care center. Indian Journal of Otology.

[70] Kelly AF, Kelly PK, Shah M (2021). Auditory brainstem response pass rates correlate with newborn hour of life and delivery mode. J Pediatr.

[71] Shim J, Kim H, Kwon Y, Chang J, Park E, Im GJ (2021). Results of a 10-year hearing screening using automated auditory brainstem response in newborns: the two-step AABR method. Int J Pediatr Otorhinolaryngol.

[72] Arora RD, Jati M, Nagarkar NM, Galhotra A, Agrawal S, Mehta R (2022). Experience, challenges and outcome of implementing universal new born hearing screening in a medical college hospital set up. Indian J Otolaryngol Head Neck Surg.

[73] Gharibi R, Khavidaki GAD (2022). Assessment of the efficacy of hearing screening program in infants in Zahedan. J Family Med Prim Care.

[74] Hajare P, Mudhol R (2022). A Study of JCIH (Joint Commission on Infant Hearing) Risk Factors for Hearing Loss in Babies of NICU and Well Baby Nursery at a Tertiary Care Center. Indian J Otolaryngol Head Neck Surg.

[75] Khaimook W, Suwanno R, Dindamrongkul R, Intusoma U (2022). An Early Hearing Detection and Intervention Program in Songklanagarind Hospital. J Health Sci Med Res JHSMR.

[76] Mazlan R, Raman K, Abdullah A (2022). A 10-year retrospective analysis of newborn hearing screening in a tertiary hospital in Malaysia. Egypt J Otolaryngol.

[77] Dutra MRP, Cavalcanti HG, Ferreira MÂF (2023). Access to pediatric hearing healthcare in Rio Grande do Norte, Brazil. ABCS Health Sci.

[78] Badusha MM, Sangma R, Raj M, Raj AT (2024). Audiological screening of high-risk infants and incidence of hearing impairment. International Journal of Pharmaceutical and Clinical Research.

[79] Kgare KS, Joubert K (2024). Community-based infant hearing screening: outcomes of a rural pilot programme. S Afr J Commun Disord.

[80] Jennings A, Isaacson G (2025). Newborn hearing screening success… patient care failure. Laryngoscope.

[81] Chandrakapure D, Sachdeva K, Sharma D, Nema S (2025). Effectiveness of universal newborn hearing screening at a government tertiary health care centre in central india: a retrospective study. Indian J Otolaryngol Head Neck Surg.

[82] Ndoleriire C, Böttcher P, Hinterholzer M, Anyait S, Namingira SP, Musinzi J (2025). Quality controlled neonatal hearing screening in children 0–3 months at post natal wards in Uganda: a multi-center study. Int J Pediatr Otorhinolaryngol.

[83] Parangrit K, Sillabutra J, Isaradisaikul SK, Kulprachakarn K (2025). Comparative study of the efficacy between new and routine newborn hearing screening protocols in public hospital, Thailand. J Matern Fetal Neonatal Med.

[84] Yoshinaga-Itano C, Manchaiah V, Hunnicutt C (2021). Outcomes of Universal Newborn Screening Programs: systematic Review. J Clin Med.

[85] Butcher E, Dezateux C, Cortina-Borja M, Knowles RL (2019). Prevalence of permanent childhood hearing loss detected at the universal newborn hearing screen: systematic review and meta-analysis. PLoS One.

[86] Edmond K, Chadha S, Hunnicutt C, Strobel N, Manchaiah V, Yoshinga-Itano C (2022). Effectiveness of universal newborn hearing screening: a systematic review and meta-analysis. J Glob Health.

[87] Manz K, Nennstiel U, Marzi C, Mansmann U, Brockow I (2025). Quality measures of two-stage newborn hearing screening: systematic review and meta-analysis. Front Public Health.

[88] Faistauer M, Silva AL, Dominguez DDOR, Bohn R, Félix TM, Costa SSD (2022). Does universal newborn hearing screening impact the timing of deafness treatment?. J Pediatr (Rio J).

[89] Korver AMH, Smith RJH, Van Camp G, Schleiss MR, Bitner-Glindzicz MAK, Lustig LR (2017). Congenital hearing loss. Nat Rev Dis Primers.

[90] Zhu Q-W, Li M-T, Zhuang X, Chen K, Xu W-Q, Jiang Y-H (2021). Assessment of hearing screening combined with limited and expanded genetic screening for Newborns in Nantong, China. JAMA Netw Open.

[91] Pan K, Shang Z, Liu J, Wen Y, Luo J, Zou D (2024). Newborn concurrent hearing and genetic screening for hearing impairment: a systematic review and meta‑analysis. Exp Ther Med.

[92] Martins MFP, Donadon C, Skarzynski PH, De Souza AJT, De Andrade AN, Gil D (2025). Auditory steady-state responses for detecting mild hearing loss in babies, infants, and children: literature review. Life (Basel).

[93] Bower C, Reilly BK, Richerson J, Hecht JL, Hackell JM, Almendarez YM (2023). Hearing assessment in infants, children, and adolescents: recommendations beyond neonatal screening. Pediatrics.

[94] Wu K, Lan L, Shi W, Li J, Xie L, Xiong F (2022). The audiological characteristics of infant auditory neuropathy patients without otoacoustic emission. Laryngoscope Investig Otolaryngol.

[95] Gáborján A, Katona G, Szabó M, Muzsik B, Kustel M, Horváth M (2022). Universal newborn hearing screening with automated auditory brainstem response (AABR) in Hungary: 5-year experience in diagnostics and influence on the early intervention. Eur Arch Otorhinolaryngol.

[96] Alothman N, Elbeltagy R, Mulla R (2024). Universal newborn hearing screening program in Saudi Arabia: current insight. J Otol.

