# Supplementary material for: A survey of strategies for the early diagnosis of hearing loss in infants: a scoping review
Source: Codas. 2026 Jul 31;38(5):e20250375. doi: 10.1590/2317-1782/e20250375en (PMC13440685; doi:10.1590/2317-1782/e20250375en)
Supplement: Translation

# Translation: Um levantamento das estratégias para o diagnóstico precoce da perda auditiva em lactentes: uma revisão de escopo

**DOI:** 10.1590/2317-1782/e20250375pt

**Authors:** Allan Dayner Silva Lopes, Antônio Pereira de Carvalho, Sanmara de Andrade Silva, Rodrigo Oliveira da Fonseca, Maiara Cristine Oliveira de Almeida, Maria Helena Medeiros de Sá Lima Lucena, Karinna Veríssimo Meira Taveira, Hannalice Gottschalck Cavalcanti

**Affiliations:** 1 Programa Associado de Pós-graduação em Fonoaudiologia, Universidade Federal da Paraíba – UFPB - João Pessoa (PB), Brasil.; 2 Departamento de Fonoaudiologia, Universidade Federal da Paraíba – UFPB - João Pessoa (PB), Brasil.; 3 Programa Associado de Pós-graduação em Fonoaudiologia, Universidade Federal do Rio Grande do Norte Natal – UFRN - Natal (RN), Brasil.; 4 Departamento de Morfologia, Universidade Federal do Rio Grande do Norte Natal – UFRN - Natal (RN), Brasil.

**Keywords:** Triagem Neonatal, Perda Auditiva, Bebê, Diagnóstico Precoce, Revisão de Escopo

## Abstract

**Objetivo:**

Mapear as estratégias utilizadas para o diagnóstico precoce da perda auditiva em lactentes.

**Estratégia de pesquisa:**

A busca bibliográfica foi realizada nas bases PubMed/Medline, Scopus, Embase, Web of Science, LILACS, Google Scholar e ProQuest, sem restrição de idioma ou período de publicação, inicialmente realizada em outubro de 2024 e atualizada em janeiro de 2026. Utilizados descritores controlados (DeCS/MeSH) e palavras-chave relacionados à perda auditiva, triagem auditiva neonatal e diagnóstico precoce. O protocolo foi registrado na Open Science Framework.

**Critérios de seleção:**

Foram incluídos estudos primários que abordaram estratégias de diagnóstico precoce da deficiência auditiva em crianças de até dois anos. Excluíram-se estudos secundários, relatos de caso, diretrizes, artigos duplicados ou sem texto completo. A seleção foi realizada por revisores independentes, com consenso em caso de divergência.

**Análise dos dados:**

As informações extraídas abrangeram delineamento, população, instrumentos utilizados, estratégias diagnósticas e contexto assistencial. A síntese foi descritiva e narrativa, com agrupamento das evidências por tipo de abordagem.

**Resultados:**

Dos 16.946 registros identificados, 58 estudos, publicados entre 1991 e 2025, atenderam aos critérios de elegibilidade. Predominaram estratégias baseadas na Triagem Auditiva Neonatal Universal (TANU), com associação entre emissões otoacústicas e potencial evocado auditivo de tronco encefálico automatizado. Estratégias complementares incluíram protocolos em dois estágios, reteste precoce, programas comunitários, triagem genética e exames eletrofisiológicos adicionais. Observou-se variabilidade na operacionalização dos protocolos e persistência de desigualdades estruturais.

**Conclusão:**

A TANU permanece como eixo central do diagnóstico precoce da perda auditiva em lactentes, com efetividade dependente da organização dos fluxos assistenciais e da integração entre triagem, diagnóstico e acompanhamento.

## INTRODUÇÃO

A audição desempenha papel central no desenvolvimento infantil, estando diretamente relacionada à aquisição da linguagem, à comunicação e às habilidades cognitivas e sociais^(1,2)^. A privação auditiva nos primeiros anos de vida pode acarretar prejuízos duradouros nos âmbitos educacional, emocional e social, tornando a identificação e a intervenção precoces fundamentais para a mitigação desses impactos^(1-3)^.

Nesse contexto, os programas de Triagem Auditiva Neonatal Universal (TANU) consolidaram-se como importante política de saúde pública em diversos países, com o objetivo de favorecer a detecção precoce da perda auditiva^(4,5)^. Entretanto, desafios estruturais, organizacionais e contextuais — evidenciados de forma mais intensa durante a pandemia da COVID-19 — têm comprometido a continuidade desses programas, resultando em atrasos no diagnóstico, na intervenção e no seguimento assistencial^(5-7)^.

As diretrizes nacionais e internacionais recomendam a utilização combinada das Emissões Otoacústicas Evocadas (EOAE) e do Potencial Evocado Auditivo de Tronco Encefálico Automático (PEATE-A) como protocolo padrão-ouro para a triagem auditiva neonatal, a ser realizada preferencialmente nas primeiras 48 horas de vida ou até os 30 dias após o nascimento^(2,6,8-10)^. Contudo, a existência de recomendações formais não assegura sua implementação homogênea na prática clínica. Diferenças entre sistemas de saúde, setores público e privado, bem como falhas na integração das redes assistenciais, resultam em variações nos fluxos diagnósticos e na efetividade do acompanhamento^(8-11)^.

A literatura aponta que as estratégias utilizadas para o diagnóstico precoce da deficiência auditiva em lactentes permanecem fragmentadas, frequentemente centradas em tecnologias ou populações específicas, sem considerar de forma integrada aspectos organizacionais, socioeconômicos e geográficos que influenciam o acesso aos serviços e a continuidade do cuidado^(8,12-14)^. Essa heterogeneidade dificulta a comparação entre contextos e limita a identificação de padrões que subsidiem a qualificação das práticas assistenciais e das políticas públicas de saúde auditiva^(9,13,15)^.

Diante da relevância epidemiológica da perda auditiva infantil e dos desafios persistentes relacionados à sua detecção e intervenção precoces^(12,13,16-19)^, observa-se uma lacuna na literatura quanto à síntese crítica das estratégias empregadas para o diagnóstico precoce, abrangendo não apenas os exames utilizados, mas também os fluxos assistenciais, protocolos, intervenções educativas e políticas de saúde. Assim, o objetivo desta revisão de escopo foi mapear as estratégias utilizadas para o diagnóstico precoce da perda auditiva em lactentes, reunindo e sintetizando as evidências científicas disponíveis, a fim de contribuir para o aprimoramento das práticas clínicas e o fortalecimento das políticas públicas de saúde auditiva infantil.

## MÉTODOS

Esta revisão de escopo foi conduzida segundo as recomendações metodológicas do Joanna Briggs Institute - JBI^(20)^ e reportada conforme o checklist do Preferred Reporting Items for Systematic Reviews and Meta-Analyses Extension for Scoping Reviews - PRISMA-ScR^(21)^. O protocolo do estudo foi previamente registrado na plataforma Open Science Framework (OSF)^(22)^.

### Critérios de Elegibilidade

A pergunta de pesquisa foi formulada a partir da estratégia **PCC** (População, Conceito e Contexto), definida da seguinte forma:

– lactentes (0–2 anos);
  – estratégias utilizadas para o diagnóstico precoce da perda auditiva;
    – serviços e contextos de atenção à saúde auditiva.

Assim, a pergunta norteadora foi: *“Quais estratégias têm sido utilizadas para o diagnóstico precoce da perda auditiva em lactentes?”*

### Critérios de inclusão

Foram incluídos estudos que abordassem estratégias, protocolos, programas ou métodos aplicados ao diagnóstico precoce da deficiência auditiva em crianças de até dois anos de idade, estudos primários (observacionais transversais, de coorte, caso-controle, quase-experimentais ou experimentais e ensaios metodológicos). Não houve restrições quanto ao idioma ou data de publicação.

A literatura cinzenta (teses, dissertações, documentos institucionais e relatórios técnicos) foi excluída, com o objetivo de garantir maior reprodutibilidade, padronização metodológica e comparabilidade dos dados, priorizando estudos publicados em periódicos científicos revisados por pares.

### Critérios de exclusão

Foram excluídos estudos que envolvessem participantes fora da faixa etária proposta, estudos que não se enquadrassem nos critérios de inclusão previamente definidos, estudos que não abordassem estratégias específicas de avaliação ou diagnóstico da perda auditiva, ou que não especificassem a idade de detecção. Foram também excluídos estudos secundários (revisões sistemáticas, narrativas, integrativas e meta-análises), diretrizes clínicas, relatos de caso, séries de casos, artigos de opinião, textos técnicos, livros, websites, blogs e demais fontes não científicas. Artigos que não foram encontrados na íntegra, após o contato com os autores.

### Estratégia de busca

A busca bibliográfica foi inicialmente conduzida na base de dados PubMed/Medline e adaptada para as demais bases: Embase, Scopus, Web of Science, LILACS, Google Scholar e ProQuest, em 15 de outubro de 2024 e atualizada em 05 de janeiro de 2026 (Apêndice A). No Google Scholar, foram analisadas as primeiras 200 referências ordenadas por relevância, conforme recomendação metodológica para revisões de escopo.

A estratégia foi adaptada a cada base de dados e combinou descritores controlados e não controlados (MeSH, DeCS e palavras-chave livres) relacionados aos conceitos principais do estudo, utilizando operadores booleanos AND e OR, além de truncamentos e variações ortográficas, conforme as especificidades de cada base de dados.

### Gerenciamento das referências e triagem

Os resultados das buscas foram exportados inicialmente para o software Zotero (versão 7.0), utilizado exclusivamente para organização das referências e remoção automática de duplicatas. Em seguida, os registros foram importados para a plataforma Rayyan^(23)^, onde foi realizada uma verificação manual adicional de duplicatas, a fim de aumentar a acurácia do processo, além da triagem dos títulos e resumos.

Antes do início da seleção, os revisores participaram de uma etapa de calibração, com análise conjunta de uma amostra piloto de estudos, visando padronizar a aplicação dos critérios de elegibilidade.

### Seleção dos estudos

A seleção dos estudos foi realizada em duas etapas. Na primeira, os títulos e resumos foram avaliados de forma independente por seis revisores, organizados em três duplas (dupla 1: ADSL e SAS; dupla 2: APCF e ROF; dupla 3: MCOA e MHM). A divisão em três duplas teve como objetivo distribuir o volume de registros de forma equilibrada, garantindo maior agilidade e rigor na triagem inicial.

Cada base de dados foi analisada por pelo menos uma dupla de revisores, assegurando a aplicação uniforme dos critérios de elegibilidade. Na segunda etapa, os textos completos dos estudos potencialmente elegíveis foram lidos integralmente pelos mesmos revisores.

As decisões foram tomadas inicialmente por consenso dentro de cada dupla. Quando não havia concordância, o estudo era discutido em reunião com todas as duplas. Persistindo a divergência, um terceiro revisor independente foi consultado para decisão final. Durante todas as etapas, os revisores permaneceram cegados quanto às decisões dos demais pares, conforme funcionalidade da plataforma Rayyan.

### Extração e análise dos dados

Os dados foram extraídos por meio de um formulário estruturado elaborado pelos autores, contemplando: (a) identificação do estudo (autores, ano, país, idioma e tipo de publicação); (b) características metodológicas (delineamento, amostra, instrumentos utilizados e população-alvo); (c) descrição das estratégias de diagnóstico precoce aplicadas; (d) principais achados e conclusões.

A síntese dos resultados foi realizada de forma descritiva e narrativa, com apresentação em tabelas e gráficos que agrupam as estratégias identificadas segundo o tipo de abordagem (tecnológica, clínica, educativa ou política). Quando aplicável, foram destacadas lacunas de conhecimento e recomendações futuras para a área da saúde auditiva infantil.

## RESULTADOS

O processo de busca e seleção dos estudos está representado no fluxograma PRISMA-ScR (Figura 1). Foram inicialmente identificados 16.946 registros nas bases de dados consultadas. Após a remoção de duplicatas e leitura de títulos e resumos, 413 artigos foram selecionados para leitura na íntegra, destes, 355 foram excluídos (Apêndice B). Diante disso, 58 estudos atenderam aos critérios de elegibilidade e compuseram a amostra final. As exclusões decorreram principalmente da ausência de foco em diagnóstico precoce, da inclusão de faixas etárias superiores a dois anos e da utilização de delineamentos incompatíveis com o escopo da revisão.

**Figura 1:** Diagrama de fluxo conforme diretrizes do PRISMA-ScR^(25)^ (adaptado)

*Sempre que viável, informe o número de registros identificados em cada base de dados ou registro pesquisado, em vez de apresentar apenas o número total de registros recuperados em todas as bases de dados ou registros; **Se ferramentas de automação tiverem sido utilizadas, informe quantos registros foram excluídos por avaliadores humanos e quantos foram excluídos pelas ferramentas de automação

A caracterização geral dos artigos incluídos está apresentada na Tabela 1. As publicações abrangeram o período de 1991 a 2025, com aumento expressivo a partir de 2015 (n=20)^(64-83)^. A distribuição temporal das publicações está representada na Figura 2.

**Tabela 1:** Caracterização dos artigos incluídos na revisão

	**Autores (Ano)**	**Características Metodológicas**		**Procedimentos**	**Desfecho**
		**Tipo de Estudo**	**Amostra**		
**1**	Webb e Stevens (1991)^(26)^	Comparativo	250	EOA / PEATE	A triagem auditiva neonatal deve iniciar com EOAE devido à sua eficiência e custo-benefício. O ABR deve ser utilizado para confirmação e diferenciação do tipo de perda auditiva.
**2**	Tudehope et al. (1992)^(27)^	Prospectivo	149	PEATE / BOA / IMIT	A avaliação auditiva precoce em neonatos MBP é essencial para identificar perdas auditivas, especialmente as condutivas. A combinação de ABR, audiometria comportamental e imitanciometria oferece uma abordagem abrangente para o diagnóstico auditivo.
**3**	Morlet et al. (1998)^(28)^	Prospectivo	1.531	EOAT / PEATE	O processo de triagem identificou com sucesso a perda auditiva em uma população de alto risco, destacando a necessidade de exames repetidos durante o primeiro ano para crianças com resultados patológicos iniciais.
**4**	Aidan et al. (1999)^(29)^	Coorte	1727	EOAT	O estudo demonstra a viabilidade e as limitações das EOAT como uma ferramenta universal de triagem auditiva para neonatos
**5**	Watkin e Baldwin (1999)^(30)^	Retrospectivo	100	EOAT / PEATE / BOA	A triagem auditiva neonatal é eficaz na identificação precoce de deficiência auditiva, mas é essencial garantir o seguimento adequado e a comunicação eficaz com os pais para confirmar o diagnóstico e iniciar a intervenção precoce.
**6**	Prieve et al. (2000)^(31)^	Prospectivo	43.311	EOAT / PEATEa / PEATE	Os indicadores ambulatoriais melhoraram ao longo dos anos, com diferenças significativas entre recém-nascidos da UTI neonatal e das enfermarias de rotina, além de disparidades regionais que necessitam análise adicional.
**7**	Shehata-Dieler et al. (2000)^(32)^	Prospectivo	1.349	PEATE / EOA	O artigo conclui que o método BeRapHon® é uma ferramenta eficaz e altamente específica para o rastreamento auditivo universal em recém-nascidos.
**8**	Stewart et al. (2000)^(33)^	Prospectivo	11.711	PEATEa / PEATE / TIMP / EOAT	A triagem auditiva universal com PEATEa se mostrou altamente efetiva e viável nos diferentes hospitais avaliados, com baixo impacto sobre custos de seguimento e baixa interferência no vínculo entre pais e bebê.
**9**	Baumann e Schorn (2001)^(34)^	Coorte	102	EOAT / BOA / PEATE	O método da *bandinha* mostrou-se inadequado para a triagem auditiva, com detecção de apenas 61,5% dos casos. Em contrapartida, o teste de emissões otoacústicas demonstrou-se eficaz na identificação de perdas auditivas em crianças. Além disso, dispositivos automatizados, como o *Echoscreen*, apresentaram alta especificidade para a detecção da perda auditiva.
**10**	Owen et al. (2001)^(35)^	Prospectivo	683	EOA	O modelo de triagem auditiva neonatal comunitária conduzida por agentes de saúde é viável, bem aceito, com alta cobertura populacional e baixa taxa de falso positivos.
**11**	Lin et al. (2002)^(36)^	Prospectivo	6.765	EOAT / PEATE	O uso de EOAT como triagem auditiva universal neonatal mostrou-se viável, preciso e custo-efetivo.
**12**	Iwasaki et al. (2003)^(37)^	Prospectivo	4.085	PEATEa / PEATE / EOA / BOA	O teste de triagem PEATEa em dois estágios reduziu efetivamente as taxas de referência e as taxas de falsos positivos em recém-nascidos.
**13**	Rouev et al. (2004)^(38)^	Prospectivo	1.838	PEATEa / PEATE	A TANU na Bulgária utilizando um protocolo de rastreamento de duas fases com PEATEa e PEATE, é um método eficaz e custo-efetivo para a detecção precoce da perda auditiva em recém-nascidos, apesar dos desafios relacionados com o seguimento dos casos referenciados.
**14**	White et al. (2005)^(39)^	Prospectivo	86.634	EOA / PEATEa	O protocolo de triagem auditiva neonatal utilizando EOA e PEATEa identificou a maioria dos bebês com perda auditiva permanente, mas uma proporção significativa teria passado no PEATEa.
**15**	Widen et al. (2005)^(40)^	Prospectivo	973	EOA / PEATEa / VRA	A triagem auditiva neonatal deve incluir monitoramento contínuo além do período neonatal. A VRA é um método apropriado para a maioria dos bebês, mas a experiência do examinador são fatores importantes para a eficácia do diagnóstico.
**16**	Yee-Arellano et al. (2006)^(41)^	Prospectivo	3.066	PEATEa / PEATE	Mesmo com amostra reduzida (fase piloto), as taxas de perda auditiva identificadas são semelhantes às observadas em outros países. O estudo destaca a viabilidade do programa e a urgência em expandi-lo, enfatizando atenção aos aspectos operacionais e de seguimento clínico.
**17**	De Capua et al. (2007)^(42)^	Prospectivo	19.700	EOA / EOA / PEATE / BOA	O PEATE de diagnóstico é preferível ao PEATE automatizado para melhor precisão.
**18**	Tatli et al. (2007)^(43)^	Prospectivo	711	EOAT / PEATE	O programa de triagem auditiva neonatal utilizando EOAT em duas etapas é viável e bem aceito pelas famílias. A triagem eficaz foi alcançada em 96% dos neonatos na enfermaria.
**19**	Olusanya et al. (2008)^(44)^	Transversal	1.330	EOAT / PEATE	A triagem auditiva neonatal universal em ambiente hospitalar é viável em contextos urbanos da Nigéria, mesmo conduzida por profissionais não especializados, após treinamento breve. É eficaz na detecção, com baixa taxa de encaminhamento.
**20**	Dantas et al. (2009)^(45)^	Retrospectivo	1.626	EOAT	Os resultados reforçam a importância da implantação e manutenção de programas de triagem auditiva neonatal, sendo útil para comparações em estudos regionais ou multinacionais.
**21**	Ohl et al. (2009)^(46)^	Retrospectivo	1.461	EOAa / EOA / PEATE	O programa de triagem em bebês de risco permitiu o acesso precoce ao manejo de 60 crianças surdas, embora a perda após a triagem tenha sido significativa.
**22**	Geal-Dor et al. (2010)^(47)^	Comparativo	1.545	EOAT / BOA	A triagem neonatal com EOAT é objetiva, favorecendo a detecção precoce e maior adesão. A triagem comportamental subsequente é útil para identificar déficits auditivos tardios ou progressivos, incluindo casos de neuropatia auditiva. Os métodos apresentam caráter complementar, não substitutivo.
**23**	Tasci et al. (2010)^(48)^	Retrospectivo	16.975	EOAT / PEATE	Protocolo de três etapas é preciso e não invasivo; sugere-se reorganização para duas etapas (EOAT inicial + PEATE) para ampliar cobertura.
**24**	Yu et al. (2010)^(49)^	Retrospectivo	6.336	EOAPD / PEATEa	O protocolo combinado de triagem de EOAPD e PEATEa é um protocolo de triagem viável e mais eficiente.
**25**	Daniel e Lim (2012)^(50)^	Retrospectivo	100.237	EOA / PEATEa / PEATE	O programa de triagem auditiva neonatal em Singapura foi eficaz na detecção precoce da deficiência auditiva, proporcionando oportunidades para intervenções precoces.
**26**	Friderichs et al. (2012)^(51)^	Transversal	6.227	EOAPD	A baixa cobertura indica a necessidade de profissionais dedicados (em vez da equipe regular da clínica) para alcançar uma taxa de triagem mais abrangente e eficaz.
**27**	Lim et al. (2012)^(52)^	Retrospectivo	10.879	PEATEa / PEATE / TIMP	O protocolo de triagem com PEATEa mostrou valores aceitáveis de encaminhamento e valor preditivo.
**28**	Martines et al. (2012)^(53)^	Comparativo	412	EOAT / TIMP / PEATE	Há alta prevalência de PASN em lactentes de risco; fatores como história familiar e internação em UTI neonatal são preditores independentes. A triagem combinada (TEOAE + ABR) apresenta melhor desempenho que o uso isolado de TEOAE, especialmente para detecção de neuropatia auditiva em recém-nascidos de alto risco.
**29**	Zhang et al. (2012)^(54)^	Prospectivo	10.043	EOAPD / PEATEa / Triagem genética	A triagem auditiva associada à triagem genética pode melhorar a identificação de recém-nascidos com risco de perda auditiva, incluindo casos com mutações recessivas ou induzidas por medicamentos.
**30**	Arslan et al. (2013)^(55)^	Prospectivo	2229	EOATa / PEATEa	Um teste viável para executar a TAN, bem como se a EOATa em dois estágios reduz as taxas de falsos positivos de forma eficaz.
**31**	Borkoski Barreiro et al. (2013)^(56)^	Retrospectivo	26.717	EOATa / EOAT / PEATE / PEAEE	O programa de detecção precoce da perda auditiva alcançou cobertura superior a 95%, porém apresentou taxa de encaminhamento para diagnóstico abaixo das recomendações. Apesar disso, mostrou-se adequado para a rotina hospitalar, garantindo cobertura significativa.
**32**	Hsu et al. (2013)^(57)^	Prospectivo	3.361	EOAT / PEATEa	O programa universal de triagem auditiva neonatal da PEATEa mostra alta cobertura e baixas taxas de referência.
**33**	Kuki et al. (2013)^(58)^	Comparativo	50	BOA / EOAT / PEATEa / PEATE / PEAEE	A análise observacional indica que combinando BOA com EOAT e/ou PEATEa, é possível obter triagem auditiva precisa, econômica e bem aceita. O BOA, pela sua facilidade, baixo custo e boa aceitabilidade, pode ser especialmente útil em locais com recursos limitados e onde a triagem auditiva universal ainda não está implementada.
**34**	Mishra et al. (2013)^(59)^	Prospectivo	1.101	EOAPD / PEATE	A combinação dos testes EOAPD e PEATE é eficaz na triagem auditiva neonatal universal e na detecção precoce de perda auditiva em crianças menores de 2 anos.
**35**	Qi et al. (2013)^(60)^	Prospectivo	10.983	EOAT / PEATE	É viável e prático alcançar altas taxas de cobertura na triagem de perda auditiva e reduzir as taxas de encaminhamento em recém-nascidos de trabalhadores migrantes.
**36**	Amini et al. (2014)^(61)^	Caso-Série	149	EOAT / TIMP / PEATE	Existe uma relação significativa entre peso médio ao nascer e EOA alterada e a EOA alterada em recém-nascidos asfixiados foi de 2%.
**37**	Çelik et al. (2014)^(62)^	Retrospectivo	142.128	EOAT / PEATE	A triagem auditiva mostrou-se essencial para todos os recém-nascidos, garantindo suporte ao desenvolvimento. Lactentes com fatores de risco requerem repetição dos testes para diagnóstico precoce, e melhorias no pré-natal podem contribuir para a redução das taxas de déficit auditivo.
**38**	Huang et al. (2014)^(63)^	Prospectivo	13.315	EOAT / PEATEa / PEATE	A combinação de EOAT e PEATEa, seguida de avaliação auditiva completa, constitui um modelo eficaz de TANU para recém-nascidos de alto risco na UTIN.
**39**	Canet et al. (2016)^(64)^	Retrospectivo	14.247	EOA / EOA / PEATE	O programa mostrou-se útil, porém necessita de melhorias nos processos de rastreamento e acompanhamento. Sua eficácia é limitada por perdas de seguimento, reforçando a necessidade de registro eletrônico e coordenação específica.
**40**	Chrobok et al. (2019)^(65)^	Descritivo	-	EOA / PEATE	As técnicas de triagem auditiva neonatal (EOAT e PEATE) são eficazes, objetivas e rápidas, e seu uso universal é essencial para identificar precocemente perdas auditivas e iniciar intervenção antes dos 6 meses
**41**	Wasser et al. (2019)^(66)^	Prospectivo	179.000	EOAT / PEATE	O programa nacional de triagem auditiva neonatal em Israel demonstrou alta cobertura e reduziu significativamente as idades ao diagnóstico e início da habilitação para bebês com deficiência auditiva.
**42**	Dey et al. (2020)^(67)^	Prospectivo	264	EOAPD / PEATE	A deficiência auditiva estava associada à hiperbilirrubinemia que requer fototerapia ou troca de transfusão, pode ser um indicador preditivo para o desenvolvimento da deficiência auditiva.
**43**	McInerney et al. (2020)^(68)^	Retrospectivo	604	EOAPD / PEATE	Os resultados destacam a necessidade de focar nas barreiras específicas que afetam a perda de acompanhamento. Recomendamos o uso de ferramentas para o engajamento parental
**44**	Nishad et al. (2020)^(69)^	Prospectivo	1.000	EOAT / PEATE	Um protocolo bem estruturado, com triagem EOA inicial e reteste com PEATE, é necessário para detecção precoce da perda auditiva—maior incidência foi observada em neonatos de alto risco; EOA é útil para triagem rápida, mas o PEATE é essencial para diagnóstico definitivo
**45**	Kelly et al. (2021)^(70)^	Retrospectivo	31.984	PEATE	Adiar o teste PEATE para cerca de 10–11 horas após o nascimento em partos vaginais e 30–32 horas em cesáreas melhora significativamente as taxas de aprovação, e a inclusão de um terceiro teste após a saída hospitalar reduz ainda mais os encaminhamentos.
**46**	Shim et al. (2021)^(71)^	Retrospectivo	3.059	PEATEa / PEATE / TIMP / PEAEE / EOAT	O protocolo de triagem em duas etapas com PEATEa apresentou desempenho eficaz e dentro do recomendado pelo JCIH (taxa de referência de 3–4%). O estudo valida esse método como adequado para triagem auditiva neonatal em ambiente hospitalar.
**47**	Arora et al. (2022)^(72)^	Prospectivo	1200	EOAT / PEATE / PEAEE	A TAN é viável em ambientes com recursos limitados associados a programas comunitários e educação parental são essenciais para a melhoria.
**48**	Gharibi e Khavidaki (2022)^(73)^	Transversal	7.700	EOAT / PEATEa / PEATE	Um programa abrangente de triagem auditiva neonatal é fundamental para garantir diagnóstico e tratamento oportunos, sendo que a identificação e a intervenção precoces são decisivas para uma reabilitação eficaz da perda auditiva.
**49**	Hajare e Mudhol (2022)^(74)^	Comparativo	800	EOAPD / PEATEa / PEATE	Uma instalação centralizada para triagem poderia beneficiar hospitais sem recursos para exames universais ou de alto risco.
**50**	Khaimook et al. (2022)^(75)^	Retrospectivo	1.579	EOA / PEATE / TIMP	O programa universal de triagem auditiva neonatal é eficaz para identificar precocemente crianças com perda auditiva e encaminhá-las à intervenção, destacando o suporte familiar e a atuação interdisciplinar como fatores-chave para melhores resultados.
**51**	Mazlan et al. (2022)^(76)^	Retrospectivo	50.569	EOAT / PEATEa / PEATE	O programa atingiu excelente cobertura, mas teve desempenho abaixo do esperado em outros indicadores como encaminhamento, seguimento e diagnóstico. É necessário revisitar e aprimorar o programa atual para mantê-lo relevante e eficaz.
**52**	Dutra et al. (2023)^(77)^	Transversal	104	EOAT / VRA / BOA / PEATE / PEAEE	Falta acesso ao serviço e realização do diagnóstico e as crianças não são atendidas nas idades adequadas. A idade, a escolaridade e a distância da mãe ao serviço não influenciaram o acesso e a utilização do serviço.
**53**	Badusha et al. (2024)^(78)^	Prospectivo	117	EOAPD / PEATE	A elevada incidência de deficiência auditiva em bebês de risco reforça a necessidade da triagem neonatal. A colaboração entre profissionais de saúde e pais é essencial para a efetividade do rastreio e da in
**54**	Kgare e Joubert (2024)^(79)^	Retrospectivo	2.302	TIMP / PEATE / PEAA	A triagem auditiva neonatal universal em base comunitária mostrou-se viável em contexto rural; entretanto, elevadas taxas de perda de seguimento e idade tardia no rastreio comprometeram a efetividade do diagnóstico precoce da perda auditiva.
**55**	Jennings e Isaacson (2025)^(80)^	Retrospectivo	2.069	EOAT / PEATEa	Embora a triagem auditiva neonatal antes da alta tenha alta cobertura, a efetividade do diagnóstico precoce é comprometida por perda de seguimento ambulatorial (43%) e barreiras socioeconômicas (ex.: transporte, seguro e cuidado de outros filhos).
**56**	Chandrakapure et al. (2025)^(81)^	Retrospectivo	28.025	BOA / EOAT / PEATEa / PEATE	A triagem auditiva neonatal universal em centro terciário apresentou elevada cobertura e excelente desempenho diagnóstico; entretanto, a baixa adesão ao seguimento pós-triagem limitou a efetividade do diagnóstico precoce da perda auditiva.
**57**	Ndoleriire et al. (2025)^(82)^	Transversal	12.996	EOAT / PEATEa / PEATE / TIMP	A triagem auditiva neonatal com controle de qualidade mostrou-se viável em contexto hospitalar público; contudo, grandes variações na cobertura e elevadas taxas de perda de seguimento comprometeram a efetividade do diagnóstico precoce da perda auditiva.
**58**	Parangrit et al. (2025)^(83)^	Retrospectivo	6.472	EOA / PEATEa	A implementação de um protocolo de triagem auditiva neonatal mais precoce e estruturado aumentou a cobertura e o diagnóstico em tempo oportuno, embora a perda de seguimento permaneça como limitação relevante do fluxo assistencial.

Legenda: BOA = Behavioral Observation Audiometry; EOAa = Emissões Otoacústicas Automáticas; EOAT = Emissões Otoacústicas Transientes; EOAPD = Emissões Otoacústicas por Produto de Distorção; IMIT = Imitanciometria; PEATE = Potencial Evocado Auditivo do Tronco Encefálico; PEATEa = Potencial Evocado Auditivo do Tronco Encefálico Automático; PEAEE = Potencial Evocado Auditivo do Estado Estável; TIMP = Timpanometria; VRA = Audiometria de Reforço Visual

Fonte: Page et al.^(24)^

**Figura 2:** Distribuição temporal dos estudos incluídos na revisão

Em relação à distribuição geográfica, observou-se predominância de estudos conduzidos na América do Norte, Europa e Ásia, com destaque para Estados Unidos^(31,33,39,40,68,70,80)^, Índia^(58,59,69,72,74,78,81)^, China^(49,54,60,63)^ e Turquia^(43,46,55,62)^. A Figura 3 apresenta a distribuição geográfica dos estudos incluídos. Foram identificadas poucas publicações provenientes da América Latina (n = 3)^(41,45,77)^ e da África (n = 4)^(44,51,79,82)^.

**Figura 3:** Distribuição geográfica dos estudos incluídos na revisão. Criado com Datawrapper

Predominaram estudos observacionais descritivos, principalmente transversais e retrospectivos, que corresponderam à maior parte da amostra (n = 35) e estiveram majoritariamente vinculados a programas de Triagem Auditiva Neonatal Universal (TANU) ou a serviços hospitalares de referência. Coortes prospectivas e ensaios clínicos foram menos frequentes (n = 18), direcionados sobretudo à avaliação da acurácia diagnóstica e à validação de protocolos combinados. O tamanho amostral variou amplamente, de 50^(58)^ a 179.000^(66)^ participantes, evidenciando heterogeneidade substancial entre os estudos incluídos.

As estratégias mapeadas para o diagnóstico precoce da perda auditiva infantil incluíram predominantemente a triagem auditiva neonatal universal, com utilização de emissões otoacústicas (EOAT/EOAPD) em 89,6% dos estudos (n = 52), potencial evocado auditivo de tronco encefálico (PEATE) em 77,5% (n = 45) e versões automatizadas do PEATE (PEATEa) em 41,3% (n = 24). Protocolos em dois estágios, geralmente compostos por EOA seguidas de PEATEa, foram descritos em aproximadamente 35% dos estudos (n = 20).

Estratégias complementares também foram identificadas, incluindo abordagens direcionadas a populações de risco (n = 7)^(26,28,46,53,68,69,74,78)^, ações de educação e orientação parental (n = 2)^(68,72)^, baterias eletrofisiológicas ampliadas (n = 7)^(38,41,56,58,70-72)^ e avaliações comportamentais (n = 9)^(27,30,34,37,42,47,58,77,81)^. Em menor proporção, alguns estudos relataram a incorporação de testagens etiológicas complementares, como triagem genética associada à avaliação auditiva neonatal (n = 1)^(54)^.

No que se refere aos contextos assistenciais, a maioria dos estudos foi conduzida em maternidades e hospitais públicos (n = 52), enquanto iniciativas desenvolvidas na atenção primária à saúde ou em serviços comunitários foram menos frequentes (n = 6)^(35,47,51,53,65,79)^. As principais barreiras relatadas incluíram perda de seguimento nos retestes, falhas na comunicação entre maternidades e serviços especializados, indisponibilidade de equipamentos e dificuldades logísticas relacionadas ao acesso aos serviços^(31,34,47,52,60,66,77)^. Em contrapartida, estudos que apresentaram melhores indicadores assistenciais relataram a adoção de protocolos universais em duas etapas, estratégias de busca ativa das famílias, monitoramento informatizado dos casos e educação parental estruturada^(39,43,48,55,56,58,61,67,72,73,82)^.

Com base na síntese dos estudos incluídos, foi elaborada uma figura-síntese (Figura 4) integrando estratégias diagnósticas, contextos assistenciais e barreiras recorrentes relacionadas ao diagnóstico precoce da deficiência auditiva infantil.

**Figura 4:** Síntese das estratégias e barreiras relacionadas ao diagnóstico precoce da perda auditiva em lactentes

## DISCUSSÃO

A distribuição temporal e geográfica dos estudos incluídos revela padrões relevantes da produção científica sobre o diagnóstico precoce da perda auditiva infantil. Observou-se aumento das publicações a partir de 2015, bem como concentração dos estudos em países de alta renda, especialmente Estados Unidos, Europa e Ásia, indicando desigualdades na produção científica e na implementação de programas estruturados de triagem.

Esta revisão de escopo evidenciou ampla variabilidade nos fluxos assistenciais e na padronização dos protocolos destinados à detecção e intervenção precoce da perda auditiva infantil^(26-78)^. Tal heterogeneidade reflete não apenas diferenças metodológicas entre os estudos, mas sobretudo desigualdades estruturais entre os sistemas de saúde, relacionadas à organização da rede assistencial, à disponibilidade de recursos e à integração entre os níveis de atenção. Ao mesmo tempo, a diversidade de contextos analisados permitiu identificar estratégias bem-sucedidas que contribuem para o diagnóstico oportuno da deficiência auditiva, mesmo em cenários com restrições logísticas^(9,26,29,36)^.

No conjunto dos estudos, a combinação de Emissões Otoacústicas e Potencial Evocado Auditivo de Tronco Encefálico manteve-se como eixo central das estratégias de detecção precoce, associando maior sensibilidade diagnóstica à antecipação do início da reabilitação auditiva^(27,34,44,72,76)^. A evolução histórica da Triagem Auditiva Neonatal Universal, inicialmente direcionada a neonatos de alto risco e posteriormente ampliada para toda a população neonatal, mostrou impacto consistente na redução da idade média de diagnóstico e no acesso às intervenções auditivas, independentemente do contexto assistencial^(26-29,38,60,76,84)^.

Apesar de sua ampla adoção, os achados desta revisão indicam que a efetividade da TANU está fortemente condicionada à forma como os protocolos são operacionalizados. A utilização de protocolos em dois estágios, com aplicação sequencial de EOA e PEATE automatizado, mostrou-se eficaz na redução de falsos positivos e na otimização da especificidade diagnóstica^(81,85-87)^. Além disso, o reteste realizado antes da alta hospitalar ou nas primeiras 48–72 horas de vida esteve associado a melhores taxas de adesão e menor encaminhamento desnecessário, minimizando interferências transitórias do período neonatal^(8,33,37,41,52,71,80,87)^. Esses achados reforçam que a qualidade do fluxo assistencial é tão determinante quanto a escolha do método diagnóstico.

Aspectos programáticos e de gestão surgiram como determinantes críticos para o desempenho dos programas de triagem. A coordenação por profissionais especializados, a isenção de custos nos estágios subsequentes e a integração com redes regionais e nacionais de saúde estiveram consistentemente associadas à redução das perdas de seguimento^(8,78,82)^. Esses resultados sustentam a interpretação de que a eficácia da TANU depende menos da tecnologia isolada e mais da organização dos serviços e da continuidade do cuidado.

Modelos de triagem comunitária, especialmente aqueles que incorporaram agentes comunitários de saúde, demonstraram potencial para ampliar a cobertura e reduzir a evasão, sobretudo em áreas rurais ou de difícil acesso^(35,44)^. Tais experiências indicam que estratégias territorializadas podem mitigar barreiras logísticas e geográficas, contribuindo para maior equidade no acesso aos serviços de saúde auditiva^(35,44,79)^.

Outro achado relevante refere-se às limitações da triagem auditiva como evento único. Estudos incluídos demonstraram que lactentes que “passam” na triagem inicial podem desenvolver perda auditiva de início tardio, resultando em diagnóstico e intervenção tardios^(40,66,68,88)^. Essa evidência reforça a necessidade de monitoramento clínico e etiológico contínuo, com reconvocação periódica para exames complementares, como o PEATE diagnóstico e a audiometria comportamental, ao longo dos primeiros anos de vida.

Entre as estratégias complementares, a triagem genética destacou-se como abordagem promissora, especialmente na identificação precoce de perdas auditivas não detectadas pelos métodos auditivos convencionais e na prevenção de quadros associados à ototoxicidade^(54,89-91)^. De forma semelhante, o uso combinado de exames eletrofisiológicos, como o Potencial Evocado Auditivo de Estado Estável, contribuiu para a estimativa do grau e da configuração audiométrica, embora persistam controvérsias quanto à sua viabilidade em larga escala^(26,28,40,50,66,92-96)^.

De forma integrada, os achados desta revisão indicam que a efetividade dos programas de diagnóstico precoce da perda auditiva infantil resulta da articulação entre estratégias tecnológicas, organização dos serviços e integração dos diferentes níveis de atenção à saúde. A fragmentação entre triagem, diagnóstico e intervenção permanece como um dos principais entraves para o cumprimento da linha de cuidado preconizada internacionalmente^(12,88)^.

Esta revisão apresenta limitações inerentes ao seu delineamento, incluindo a heterogeneidade metodológica dos estudos e a predominância de pesquisas conduzidas em países de alta renda, o que pode restringir a generalização dos resultados. Ainda assim, ao integrar estratégias, contextos e barreiras em uma única síntese analítica, este estudo contribui para uma compreensão mais abrangente das fragilidades e potencialidades dos programas de diagnóstico precoce da perda auditiva infantil, apontando caminhos para o aprimoramento de políticas públicas e práticas assistenciais.

## CONCLUSÃO

Esta revisão de escopo mapeou as principais estratégias para o diagnóstico precoce da perda auditiva em lactentes, destacando a predominância da Triagem Auditiva Neonatal Universal baseada em protocolos combinados. Embora os métodos diagnósticos estejam consolidados, observou-se ampla variabilidade na operacionalização dos protocolos entre os diferentes contextos assistenciais, indicando que a efetividade do diagnóstico precoce depende mais da organização dos fluxos e da integração entre triagem, diagnóstico e acompanhamento do que da tecnologia isolada. A escassez de estudos oriundos de contextos socialmente vulneráveis evidencia lacunas relevantes na literatura e aponta a necessidade de futuras investigações que contemplem diferentes realidades assistenciais.

## Apêndice A Estratégia de busca nas bases de dados

**Table:**

Database	Search (October 15^th^ 2024, updated January 5, 2026)
Embase	#1. 'newborn'/exp OR 'animals, newborn' OR 'child, newborn' OR 'full term infant' OR 'human neonate' OR 'human newborn' OR 'infant, newborn' OR 'neonatal animal' OR 'neonate' OR 'neonate animal' OR 'neonatus' OR 'newborn animal' OR 'newborn animals' OR 'newborn baby' OR 'newborn child' OR 'newborn infant' OR 'newly born animal' OR 'newly born baby' OR 'newly born child' OR 'newly born infant' OR 'newborn'
	#2. 'hearing impairment'/exp OR 'auditory defect' OR 'deaf' OR 'deafness' OR 'hard of hearing' OR 'hearing damage' OR 'hearing defect' OR 'hearing difficulty' OR 'hearing loss' OR 'hypacousia' OR 'hypacousis' OR 'hypacusia' OR 'hypacusis' OR 'hypakousia' OR 'hypakusis' OR 'hypoacousia' OR 'hypoacousis' OR 'hypoacusia' OR 'hypoacusis' OR 'hypoakusis' OR 'impaired hearing' OR 'hearing impairment'
	#3. 'early diagnosis'/exp OR 'diagnosis, early' OR 'early diagnosis' OR 'newborn screening'/exp OR 'mass screening, newborn' OR 'neonatal screening' OR 'screening, newborn' OR 'newborn screening'
	#4. #1 AND #2 AND #3
	#5. #4 AND [embase]/lim NOT ([embase]/lim AND [medline]/lim)
LILACS	(mh:"Recém-Nascido" OR "Recém-Nascido" OR "Criança Recém-Nascida" OR "Crianças Recém-Nascidas" OR "Lactente Recém-Nascido" OR "Lactentes Recém-Nascidos" OR neonato neonatos "Recém-Nascido (RN)" OR "Recém-Nascidos" OR "Newborn Infant" OR "Recién Nacido" OR mh:m01.060.703.520*) AND (mh:"Perda Auditiva" OR "Perda Auditiva" OR "Deficiência Auditiva" OR hipoacusia OR "Perda Auditiva Transitória" OR "Perda da Audição" OR "Perda da Capacidade Auditiva" OR "Surdez Transitória" OR "Hearing Loss" OR "Pérdida Auditiva" OR mh:c09.218.458.341* OR mh:c10.597.751.418.341* OR mh:c23.888.592.763.393.341*) AND (mh:"Diagnóstico Precoce" OR "Diagnóstico Precoce" OR "Early Diagnosis" OR "Diagnóstico Precoz" OR mh:e01.390* OR mh:"Triagem Neonatal" OR "Triagem Neonatal" OR "Rastreamento Neonatal" OR "Rede Estadual" de "Triagem Neonatal" OR "Triagem Neonatal Universal" OR "Triagem do Recém-Nascido" OR "Neonatal Screening" OR "Tamizaje Neonatal" OR mh:e01.370.225.910* OR mh:e01.370.500.580* OR mh:e05.200.910* OR mh:e05.318.308.980.438.580.580* OR mh:n02.421.726.233.443.816* OR mh:n05.715.360.300.800.438.500.575* OR mh:n06.850.520.308.980.438.580.580* OR mh:n06.850.780.500.580*)
PubMed/ Medline	#1. "Infant"[Mesh] OR Infants OR "Infant, Newborn"[Mesh] OR "Newborn Infant" OR "Newborn Infants" OR Neonate OR Neonates OR Newborns OR Newborn
	#2. "Hearing Loss"[Mesh] OR "Hearing Impairment"
	#3. "Early Diagnosis"[Mesh] OR "Early Detection of Disease" OR "Disease Early Detection" OR "Mass Screening"[Mesh] OR "Mass Screenings" OR Screening OR Screenings OR "Neonatal Screening"[Mesh] OR "Neonatal Screenings" OR "Newborn Screening" OR "Newborn Screenings" OR "Newborn Infant Screening"
	#4. #1 AND #2 AND #3
Scopus	(TITLE-ABS-KEY ( infant OR infants OR "Newborn Infant" OR "Newborn Infants" OR neonate OR neonates OR newborns OR newborn) AND TITLE-ABS-KEY ("Hearing Loss" OR "Hearing Impairment") AND TITLE-ABS-KEY ("Early Diagnosis" OR "Early Detection of Disease" OR "Disease Early Detection" OR "Mass Screening" OR "Mass Screenings" OR screening OR screenings OR "Neonatal Screening" OR "Neonatal Screenings" OR "Newborn Screening" OR "Newborn Screenings" OR "Newborn Infant Screening" ))
Web of Science	Infant OR Infants OR "Newborn Infant" OR "Newborn Infants" OR Neonate OR Neonates OR Newborns OR Newborn (Topic) AND "Hearing Loss" OR "Hearing Impairment" (Topic) AND "Early Diagnosis" OR "Early Detection of Disease" OR "Disease Early Detection" OR "Mass Screening" OR "Mass Screenings" OR Screening OR Screenings OR "Neonatal Screening" OR "Neonatal Screenings" OR "Newborn Screening" OR "Newborn Screenings" OR "Newborn Infant Screening" (Topic)
Google Scholar	"Newborn Infant" AND "Hearing Loss" AND "Early Diagnosis" OR "Neonatal Screening"
*ProQuest*	Infant OR Infants OR "Newborn Infant" OR "Newborn Infants" OR Neonate OR Neonates OR Newborns OR Newborn (Topic) AND "Hearing Loss" OR "Hearing Impairment" (Topic) AND "Early Diagnosis" OR "Early Detection of Disease" OR "Disease Early Detection" OR "Mass Screening" OR "Mass Screenings" OR Screening OR Screenings OR "Neonatal Screening" OR "Neonatal Screenings" OR "Newborn Screening" OR "Newborn Screenings" OR "Newborn Infant Screening" (Topic)

## Apêndice B Motivos para exclusão dos estudos *(n=355)*

**Table:**

Nº	Author, Year	Reason for exclusion^*^
[1]	Pérez et al., 2009	4
[2]	Abdul Hadi et al., 2012	1
[3]	Abdulai et al., 2020	2
[4]	Abdullah et al., 2006	1
[5]	Adelola et al., 2010	5
[6]	Ahmed et al., 2018	1
[7]	Aiyer; Parikh, 2009	1
[8]	Alberti et al., 1983	4
[9]	Al-Kandari; Alshuaib, 2007	5
[10]	Alarcón Avila et al., 2025	2
[11]	Allen; Lambert, 1990	4
[12]	Almenar Latorre et al., 2002	5
[13]	Alves De Sousa et al., 1996	2
[14]	Anderssen et al., 2002	3
[15]	Angrisani et al., 2012	2
[16]	Ansari, 2022	2
[17]	Applebaum, 1999	2
[18]	Aras Öztürk et al., 2018	3
[19]	Ardic, 2017	4
[20]	Arnold et al., 2006	5
[21]	Arslan et al., 2013a	2
[22]	Arslan et al., 2013b	2
[23]	Ataoğlu et al., 2019	1
[24]	Azevedo, 1991	7
[25]	Azevedo et al., 2004	7
[26]	Azizi et al., 2016	1
[27]	Babac; Djerić; Ivanković, 2007	5
[28]	Back; Ho, 2011	3
[29]	Backous, 2002	2
[30]	Bagatto et al., 2020	1
[31]	Bai et al., 2024	1
[32]	Bansal; Gupta; Nagarkar, 2008	3
[33]	BarskyFirkser; Sun, 1997	1
[34]	Bartmeyer; Navarini; Carvalho, 2025	3
[35]	Begum et al., 2024	3
[36]	Bellia et al., 2020	1
[37]	Benito Orejas et al., 2008a	3
[38]	Benito Orejas et al., 2008b	3
[39]	Benito Orejas; Silva Rico, 2017	2
[40]	Berger et al., 2012	4
[41]	Bertoldi; Manfredi; Mitre, 2017	4
[42]	Bevilacqua et al., 2010	3
[43]	Bhalot et al., 2023	3
[44]	Bhatia et al., 2013	4
[45]	Biaggio et al., 2015	2
[46]	Bianchin et al., 2022	4
[47]	Biscegli et al., 2015	3
[48]	Bishnoi et al., 2019	2
[49]	Bonelli et al., 2000	4
[50]	Botasso; Lima; Correa, 2022a	4
[51]	Botasso; Lima; Correa, 2022b	4
[52]	Botelho et al., 2010	3
[53]	Bradford et al., 1985	2
[54]	Bradley; Barbera, 2001	2
[55]	Bravo A et al., 2017	5
[56]	Brockow et al., 2011a	3
[57]	Brockow et al., 2011b	3
[58]	Bubbico et al., 2008	1
[59]	Bubbico; Tognola; Grandori, 2017	3
[60]	Buser et al., 2003	5
[61]	Calcutt et al., 2016	5
[62]	Calevo et al., 2007	3
[63]	Campos Banãles et al., 2003	4
[64]	Campos et al., 2003	4
[65]	Canale et al., 2006	4
[66]	Cao-Nguyen; Kos; Guyot, 2007	4
[67]	Cardoso et al., 2009	4
[68]	Cassidy; Ditty, 2001	1
[69]	Ceccato et al., 1996	2
[70]	Černý et al., 2003	3
[71]	Chadha; Bais, 1997	4
[72]	Champion, 2021	4
[73]	Chang et al., 2012	1
[74]	Chen et al., 2012	4
[75]	Chen et al., 2017	4
[76]	Chibisova et al., 2014	3
[77]	Chiriboga et al., 2021	2
[78]	Choi et al., 2022	4
[79]	Chu et al., 2015	1
[80]	Chung; Oh; Park, 2020a	6
[81]	Chung; Oh; Park, 2020b	6
[82]	Cianfrone et al., 2018	4
[83]	Cianfrone et al., 2021	1
[84]	Colella-Santos et al., 2013a	4
[85]	Colella-Santos et al., 2013b	1
[86]	Coll; Régnier, 1988	6
[87]	Collins et al., 2022	7
[88]	Costa et al., 2015	3
[89]	Cox; Toro, 2001	1
[90]	Cundall, 1997	7
[91]	Curcin; Sitka; Rasinski, 1998	6
[92]	Curnock, 1993	2
[93]	Da Silva et al., 2017	2
[94]	Dalzell et al., 2000	3
[95]	Dantas et al., 2009	3
[96]	Davis; Hind, 2003	1
[97]	Daykhes et al., 2017	3
[98]	De Barros Boishardy et al., 2005	3
[99]	De González Dios; Mollar Maseres, 2005	3
[100]	De Leenheer et al., 2011	1
[101]	De Mattos et al., 2009	2
[102]	Delgado Domínguez, 2011	2
[103]	Deng et al., 2022	3
[104]	Desloovere et al., 2000	6
[105]	Dlouhá et al., 2002	7
[106]	Downs, 1995	1
[107]	Duci A et al., 2000	2
[108]	Durante et al., 2005	4
[109]	Durante et al., 2004	7
[110]	Eibenstein et al., 2014	2
[111]	Eiserman et al., 2008	4
[112]	Elpers et al., 2016	1
[113]	Farhat et al., 2014	4
[114]	Feroz, et al., 2025	3
[115]	Felden et al., 2017	7
[116]	Fernandes; Nozawa, 2010	1
[117]	Fernandez Martinez et al., 1995	1
[118]	Ferro et al., 2007	4
[119]	Findlen; Malhotra; Hunter,	1
[120]	Fink et al., 2022	1
[121]	Fitzpatrick; Durieux-Smith; Whittingham, 2010	4
[122]	François et al., 2011	1
[123]	Fujikawa; Yoshinaga-Itano, 2000	2
[124]	Fukushima et al., 2008	1
[125]	Gáborján et al., 2019	3
[126]	Gaborján et al., 2022	3
[127]	Galhotra; Sahu, 2019	3
[128]	Garbaruk et al., 2021	2
[129]	Gavilan Cellie, 1993	2
[130]	Geal-Dor et al., 2002	3
[131]	Gellrich et al., 2024	5
[132]	Gentiletti-Faenze et al., 2003	2
[133]	Georgescu; Vladareanu, 2015	4
[134]	Giordano; Marchegiani; Germiller, 2015	7
[135]	Godoy B; Bustamante M, 2006	1
[136]	González De Aledo Linos et al., 2005	3
[137]	González de Dios; Mollar Maseres; Rebagliato Russo, 2005	1
[138]	González Díaz et al., 2020	5
[139]	González; Díaz; Mora, 2022	5
[140]	González; Fernández de Soto; Torres, 2012	2
[141]	Gordo et al., 1994	3
[142]	Granell; Martin, 2011	2
[143]	Greczka et al., 2015	3
[144]	Greczka et al., 2018	1
[145]	Grégoire, 1996	3
[146]	Greville; Keith, 1978	3
[147]	Groh et al., 1999	3
[148]	Guastini et al., 2010	1
[149]	Guilhoto; Quintal; Da Costa, 2003	4
[150]	Guimarães; Barbosa, 2012	1
[151]	Gulati et al., 2022	1
[152]	Halder et al., 2025	6
[153]	Hall; Brosch; Hoffmann, 2020	1
[154]	Hanschmann; Berger, 2010	7
[155]	Harrison et al., 2000	2
[156]	Hatzopoulos et al., 2001	5
[157]	Hayes, 2002	2
[158]	Helge et al., 2005	3
[159]	Hergils, 2000	2
[160]	Hoffmann et al., 2013	1
[161]	Hosforddunn et al., 1987	5
[162]	Huang et al., 2014	4
[163]	Huang et al., 2023	2
[164]	Huang et al., 2009	3
[165]	Huang et al., 2017	4
[166]	Huarte Irujo, 2003	1
[167]	Irfan et al., 2024	2
[168]	Iwanicka-Pronicka et al., 2008	4
[169]	Izumets; Nezhivenko; Murashko, 2015	2
[170]	Jacob et al., 2022	4
[171]	Jacobson; Jacobson, 1990	1
[172]	Jacobson, 2001	6
[173]	Jakubikova et al., 1999	7
[174]	Jakubíková; Kabátová; Závodná, 2003	1
[175]	Januário et al., 2015	1
[176]	Jayne et al., 2023	3
[177]	Johnson et al., 2005	1
[178]	Kanji; Khoza-Shangase, 2012	4
[179]	Kanji; Krabbenhoft, 2018	4
[180]	Kawata et al., 2011	1
[181]	Keefe et al., 2000	2
[182]	Kemper, 2011	4
[183]	Kerschner et al., 2004	3
[184]	Kiese-Himmel; Schroff; Kruse, 1997	1
[185]	Kim et al., 2011	7
[186]	Koester; Nekahm-Heis; Zorowka, 2002	7
[187]	Konukseven et al., 2010	4
[188]	Korres et al., 2006	5
[189]	Korres et al., 2008	2
[190]	Krauss M et al., 2013	1
[191]	Krishnan, 2009	3
[192]	Kumar; Gupta; Sinha, 2017	3
[193]	Lan et al., 2024	1
[194]	Langagne et al., 2008	6
[195]	Lee-Ramos et al., 2023	1
[196]	Leonhardt; Müller, 2008	3
[197]	Lewis; Racai; Bevilacqua, 1987	2
[198]	Lewis et al., 2010	2
[199]	Lim; Daniel, 2008	1
[200]	Lima; Marba; Santos, 2005	4
[201]	Lima et al., 2010	4
[202]	Lima et al., 2015	4
[203]	Ling et al., 2015	7
[204]	Liu et al., 2014	4
[205]	Liu et al., 2001	7
[206]	Lopes et al., 2020	4
[207]	Lotfi; Movallali, 2007;	4
[208]	Low et al., 2005	3
[209]	Lowell, 1960	1
[210]	Magnani et al., 2015	1
[211]	Magnusson; Persson; Sundelin, 2001;	4
[212]	Mahmood et al., 2020	3
[213]	Maluleke; Khoza-Shangase; Kanji, 2019	1
[214]	Maniuc; Chiaburu-Chiosa, 2018	4
[215]	Marco Algarra; Pitarch Ribes; Morant Ventura, 1996	5
[216]	Marinho et al., 2020	1
[217]	Marn, 2000	1
[218]	Marn, 2012	6
[219]	Martines et al., 2007	2
[220]	Martines et al., 2014	1
[221]	Martínez et al., 2003a	4
[222]	Martínez et al., 2003b	4
[223]	Martínez-Pacheco; Ferrán de la Cierva; García-Purriños, 2016	4
[224]	Mason; Herrmann, 1998	3
[225]	Mathur; Dhawan, 2007	4
[226]	Matschke; Plath, 1985a	7
[227]	Matschke; Plath, 1985b	2
[228]	Mattos et al., 2009	2
[229]	Mauk et al., 1991	4
[230]	Maxon et al., 1993	5
[231]	Meier et al., 2004	2
[232]	Mencher, 1974	1
[233]	Meyer et al., 2012	5
[234]	Mijares Nodarse et al., 2006	2
[235]	Molini et al., 1996	2
[236]	Molteni, 2006	7
[237]	Monteiro, 2004	2
[238]	Montovani; Fioravanti; Tamashiro, 1996	2
[239]	Moradi-Joo et al., 2024	3
[240]	Morgan; Canalis, 1991	2
[241]	Mukkun, 2021	3
[242]	Murray; Javel; Watson, 1985	2
[243]	Navarro Rivero et al., 1999	4
[244]	Nennstiel-Ratzel et al., 2007	3
[245]	Newton et al., 1999	1
[246]	Ng; Yun, 1992	2
[247]	Ng et al., 2004	1
[248]	Norton et al., 2000a	1
[249]	Norton et al., 2000b	2
[250]	Obeidat et al., 2024	1
[251]	Olarte et al., 2019	4
[252]	Olusanya, 2001	4
[253]	Olusanya; Ebuehi; Somefun, 2009	1
[254]	Onoda; Azevedo; Santos, 2011	3
[255]	Paat-Ahi; Sikkut; Laarmann, 2014	5
[256]	Pastorino et al., 2005	3
[257]	Paul, 2003	2
[258]	Pedersen et al., 2008	5
[259]	Pelosi et al., 1998	1
[260]	Pitathawatchai; Khaimook; Kirtsreesakul, 2019	3
[261]	Pitathawatchai; Wanachottrakul; Prayuenyong, 2025	6
[262]	Plinkert et al., 1990	1
[263]	Plinkert; Delb, 2001a	1
[264]	Plinkert; Delb, 2001b	3
[265]	Poblano et al., 2000	1
[266]	Polski et al., 2014	3
[267]	Poncet; Chaperon, 1983	4
[268]	Prieve et al., 2013	3
[269]	Prieve; Stevens, 2000	2
[270]	Prince et al., 2003	1
[271]	Ptok, 2004	2
[272]	Radü, 1985	4
[273]	Raghuwanshi et al., 2019	3
[274]	Appanaitis; Rahimian, 2018	5
[275]	Ren et al., 2025	3
[276]	Reis et al., 2019	1
[277]	Richard et al., 2025	6
[278]	Rodrigues, 2020	2
[279]	Roman et al., 2001	4
[280]	Ruiz De La Cuesta; Juste Ruiz; Cortés Castell, 2018	3
[281]	Saitoh et al., 2002	3
[282]	Saki et al., 2017	5
[283]	Santos-Ceballos et al., 2020	7
[284]	Sarno; Clemis, 1980	2
[285]	Sassada et al., 2005	7
[286]	Sass-Lehrer, 2004	2
[287]	Satish; Anil Kumar; Viswanatha, 2019	3
[288]	Schade, 2008	2
[289]	Schauseil-Zipf; Von Wedel, 1988	5
[290]	Schmidt et al., 2007	3
[291]	Schönweiler et al., 2002	4
[292]	Sedano M; San Martín U; Rahal E, 2018	3
[293]	Seguya et al., 2021	4
[294]	Sente; Aleksov-Hatvan, 2006	2
[295]	Shang et al., 2016	4
[296]	Sharma et al., 2022	4
[297]	Shehata-Dieler et al., 2000	3
[298]	Shi et al., 2025	3
[299]	Shirane et al., 2020	4
[300]	Shulman et al., 2010	1
[301]	Solanellas Soler, 2009	2
[302]	Sun et al., 2003	7
[303]	Sun et al., 2009	4
[304]	Sun et al., 2015	4
[305]	Sun et al., 2019	7
[306]	Swanepoel; Delport; Swart, 2004	3
[307]	Szyfter et al., 2008	2
[308]	Szyfter et al., 2016	1
[309]	Tanon-Anoh; Sanogo-Gone; Kouassi, 2010	5
[310]	Tham; Lee; Joseph, 2010	1
[311]	Todd, 2006	3
[312]	Trinidad Ruiz et al., 2005	4
[313]	Tufatulin et al., 2023	2
[314]	Turan; Bas, 2019	1
[315]	Tzanakakis et al., 2016	6
[316]	Ulusoy et al., 2013	3
[317]	Ulusoy et al., 2014	2
[318]	Uppenkamp et al., 1992	1
[319]	Valencia et al., 2008	4
[320]	Van Straaten et al., 2001;	1
[321]	Van Straaten; Groote; Oudesluys-Murphy, 1996	1
[322]	Vernier et al., 2014	7
[323]	Verrue; Smets, 2012	7
[324]	Vila et al., 2004	2
[325]	Voegeli, 1979	2
[326]	Vohr et al., 2002	5
[327]	Vos; Lagasse; Levêque, 2013	3
[328]	Vranješ et al., 2012	3
[329]	Wang; Xing; Liu, 2002	7
[330]	Wang et al., 2022	3
[331]	Watkin, 1999	2
[332]	Weichbold; Nekahm-Heis; Welzl-Müller, 2005	1
[333]	Welzl-Müller; Stephan, 2001	1
[334]	Widen et al., 2000	1
[335]	Wong et al., 2021	1
[336]	Wu; Huang; Kao, 1998	7
[337]	Xu et al., 2006	2
[338]	Xu et al., 2003	2
[339]	Xu; Li, 2005a	2
[340]	Xu; Li, 2005b	3
[341]	Xu; Cheng; Yang, 2011	2
[342]	Yao et al., 2013	4
[343]	Yigit et al., 2020	4
[344]	Yılmazer et al., 2016	1
[345]	Yoshinaga-Itano, 2003	3
[346]	Yoshinaga-Itano; Apuzzo, 1998	1
[347]	Yoshinaga-Itano; Gravel, 2001	1
[348]	Yousefi et al., 2013a	3
[349]	Yousefi et al., 2013b	3
[350]	Zeng et al., 2020	3
[351]	Zhang; Li; Zheng, 2014	2
[352]	Zhang et al., 2004	2
[353]	Zhao et al., 2003	1
[354]	Zwicker; Schorn, 1990	2
[355]	Zych et al., 2018	2

* 1. Wrong study design; 2. Wrong publication type; 3. Non-specific evaluation strategy; 4. Wrong population; 5. Wrong objective; 6. no response from the author; 7. Not found.

REFERENCES

[1] Pérez MC, Gaya JA, Savio G, Perera M, Ponce de León M, Sánchez M. A 25-year review of Cuba’s screening program for early detection of hearing loss. MEDICC Rev. 2009;11(1):21-8. PMid:21483323.

[2] Abdul Hadi K, Salahaldin A, Al Qahtani A, Al Musleh Z, Al Sulaitin M, Bener A, et al. Universal neonatal hearing screening: six years of experience in Qatar. Qatar Med J. 2012;2012(2):42-50. https://doi.org/10.5339/qmj.2012.2.12. PMid:25003040.

[3] Abdulai ER, Baidoo KK, Jangu AA, Searyoh K, Ahonon Y. Otoacoustic emission hearing screening of newborns in Korle-bu teaching hospital, Ghana. Postgrad Med J Ghana. 2020;9(2):90-4. https://doi.org/10.60014/pmjg.v9i2.232.

[4] Abdullah A, Hazim MYS, Almyzan A, Jamilah AG, Roslin S, Ann MT, et al. Newborn hearing screening: experience in a Malaysian hospital. Singapore Med J. 2006;47(1):60-4. PMid:16397723.

[5] Adelola OA, Papanikolaou V, Gormley P, Lang J, Keogh IJ. Newborn hearing screening: A regional example for national care. Ir Med J. 2010;103(5):146-9. PMid:20666087.

[6] Ahmed S, Sheraz S, Malik SA, Ahmed NR, Malik SA, Farooq S, et al. Frequency of congenital hearing loss in neonates. J Ayub Med Coll Abbottabad. 2018;30(2):234-6. PMid:29938425.

[7] Aiyer RG, Parikh B. Evaluation of auditory brainstem responses for hearing screening of high-risk infants. Indian J Otolaryngol Head Neck Surg. 2009;61(1):47-53. https://doi.org/10.1007/s12070-009-0034-4. PMid:23120604.

[8] Al-Kandari JM, Alshuaib WB. Newborn hearing screening in Kuwait. Electromyogr Clin Neurophysiol. 2007;47(6):305-13. PMid:17918507.

[9] Alarcón Avila C, Saenz González AT, Vivas Gómez LF, Rivero Centeno MA, Silva Ortiz MJ, Salazar Vera LC, et al. Diagnostic performance and clinical follow-up of a universal newborn hearing screening program using OAE and ABR in a middle-income country. Int J Pediatr Otorhinolaryngol. 2025;197:112511. https://doi.org/10.1016/j.ijporl.2025.112511. PMid:40884912.

[10] Alberti PW, Hyde ML, Corbin H, Riko K, Abramovich S. An evaluation of bera for hearing screening in high-risk neonates. Laryngoscope. 1983;93(9):1115-21. https://doi.org/10.1288/00005537-198309000-00001. PMid:6888122.

[11] Allen B, Lambert G. Computerisation of V.R.O.A.: a double blind hearing screening technique. Aust J Audiol. 1990;12:11-5.

[12] Almenar Latorre A, Tapia Toca MC, Fernández Pérez C, Moro Serrano M. A combined neonatal hearing screening protocol. An Esp Pediatr. 2002;57(1):55-9. https://doi.org/10.1016/S1695-4033(02)77893-4. PMid:12139894.

[13] Alves De Sousa LC, De Toledo Piza M, Da Costa SS, Andrade MJ, Jaeger WLG. Child deafness: Early diagnosis and casuistic of Paparella Foundation. Revista Brasileira de Otorrinolaringologia 1996;62:9+11-12+14.

[14] Anderssen SH, Andresen J, Andersen R, Sponheim L. Universal neonatal hearing screening of infants with otoacoustic emissions. Tidsskr Nor Laegeforen. 2002;122(22):2187-9. PMid:12426894.

[15] Angrisani RMG, Suzuki MR, Pifaia GR, Testa JR, Sousa EC, Gil D, et al. PEATE automático em recém nascidos de risco: estudo da sensibilidade e especificidade. Rev CEFAC. 2012;14(2):223-33. https://doi.org/10.1590/S1516-18462011005000065.

[16] Ansari MS. Implementation of National Infant Screening for Hearing Program in India: Challenges, Opportunities and Suggested Framework. Indian J Otolaryngol Head Neck Surg. 2022;74(Suppl 3):4150-8. https://doi.org/10.1007/s12070-021-02873-6. PMid:36742505.

[17] Applebaum EL. Detection of hearing loss in children. Pediatr Ann. 1999;28(6):351-6. https://doi.org/10.3928/0090-4481-19990601-05. PMid:10382193.

[18] Aras Öztürk SE, Aktaş S, Karakurt LT, Develioğlu ÖN, Murat Z, Çetinkaya F, et al. The follow-up results of newborn hearing screening of gaziosmanpasa taksim research and training hospital. Turk Pediatri Ars. 2018;53(1):10-6. https://doi.org/10.5152/TurkPediatriArs.2018.5389. PMid:30083069.

[19] Ardic C. Newborn hearing screening outcomes from Rize; Turkey. Konuralp Tip Derg. 2017;9:41-5. https://doi.org/10.18521/ktd.292877.

[20] Arnold CL, Davis TC, Humiston SG, Bocchini JA Jr, Bass PF 3rd, Bocchini A, et al. Infant hearing screening: stakeholder recommendations for parent-centered communication. Pediatrics. 2006;117(5 Pt 2, Suppl 3):S341-54. https://doi.org/10.1542/peds.2005-2633N. PMid:16735261.

[21] Arslan S, Işik AU, Imamoğlu M, Topbaş M, Aslan Y, Ural A. Universal newborn hearing screening; automated transient evoked otoacoustic emissions. B-ENT. 2013;9(2):122-31. PMid:23909119.

[22] Arslan S, Isik A, Imamoglu M, Topbas M, Aslan Y, Ural A. Universal newborn hearing screening; automated transient evoked otoacoustic emissions. B-ENT. 2013;9(2):123-31. PMid:23909119.

[23] Ataoğlu E, Oğuz D, Mutluay K, Elevli M. Evaluation of newborn hearing screening results in a period of four years newborn hearing screening results. Zeynep Kamil Tip Bulteni. 2019;50:35-8. https://doi.org/10.16948/zktipb.447801.

[24] Azevedo MF. Avaliaçäo e acompanhamento audiológico de neonatos de risco. Acta AWHO. 1991;10:107-16.

[25] Azevedo RF, Paschoal CP, de Azevedo MF, dos Santos AM, Furia CLB. Avaliação da implantação de programa de triagem auditiva neonatal em hospital de nível secundário. Rev Paul Pediatr. 2004;22:77-84.

[26] Azizi A, Amirian F, Dargahi A, Beidaghi S, Mohammadi M. Evaluation of universal newborn hearing screening with TEOAE and ABR: a cross-sectional study with the literature review. Int J Trop Med. 2016;11:84-9.

[27] Babac S, Djerić D, Ivanković Z. Newborn hearing screening. Srp Arh Celok Lek. 2007;135(5-6):264-8. https://doi.org/10.2298/SARH0706264B. PMid:17633310.

[28] Back KEM, Ho SKY. Universal newborn hearing screening in Singapore general hospital. Proceedings of Singapore Healthcare. 2011;20:127. https://doi.org/10.1177/20101058110200S101.

[29] Backous DD. Diagnosis and management of hearing loss in infants and young children. Otolaryngol Clin North Am. 2002;35(4):xi-xii. https://doi.org/10.1016/S0030-6665(02)00057-9.

[30] Bagatto M, Moodie S, Fitzpatrick E, Kealey C, Campbell B, Aiken S. Status of early hearing detection and intervention programs in Canada: results from a country-wide survey. Can J Speech Lang Pathol Audiol. 2020;44:107-24.

[31] Bai JS, Gowda PRP, Naik SM, Somashekhar A. Hearing screening in high-risk neonates using distortion product oto-acoustic emission. Indian J Otolaryngol Head Neck Surg. 2024;76(1):620-5. https://doi.org/10.1007/s12070-023-04227-w. PMid:38440481.

[32] Bansal S, Gupta A, Nagarkar A. Transient evoked otoacoustic emissions in hearing screening programs-Protocol for developing countries. Int J Pediatr Otorhinolaryngol. 2008;72(7):1059-63. https://doi.org/10.1016/j.ijporl.2008.03.014. PMid:18479757.

[33] Barsky-Firkser L, Sun S. Universal newborn hearing screenings: a three-year experience. Pediatrics 1997;99(6):e4. https://doi.org/10.1542/peds.99.6.e4.

[34] Bartmeyer B, Navarini IGF, Carvalho ER. Infecção congênita pelo citomegalovírus: achados clínicos e desfecho em pacientes de um hospital pediátrico terciário de Florianópolis. Brazilian Journal of Sexually Transmitted Diseases. 2025;37:e1448. https://doi.org/10.5327/DST-2177-8264-1448.

[35] Begum A, Siddiqua H, Harshitha D, Unnisa F. Screening of hearing impairment in newborns with risk factors, detected by otoacoustic emission hearing test. International Journal of Pharmaceutical and Clinical Research. 2024;16:785-90.

[36] Bellia CGD, Artmann H, Marques JM, Lüders D, Gonçalves CGD. Brainstem Auditory Evoked Potentials in infants aged 1 to 24 months during a hearing health care service. Clinics (Sao Paulo). 2020;75:e1579. https://doi.org/10.6061/clinics/2020/e1579. PMid:33146347.

[37] Benito Orejas JI, Cano BR, Pérez DM, Fernández-Calvo JL, Gómez AA. Results of applying a universal protocol for early detection of hypoacusia in newborn infants for 42 months. Acta Otorrinolaringol Esp. 2008;59(3):96-101. https://doi.org/10.1016/S0001-6519(08)73274-6. PMid:18364200.

[38] Benito Orejas JI, Ramírez Cano B, Morais Pérez D, Fernández-Calvo JL, Almaraz Gómez A. Results of applying a universal protocol for early detection of hypoacusia in newborn infants for 42 months. Acta Otorrinolaringol Esp. 2008;59(3):96-101. https://doi.org/10.1016/S0001-6519(08)73274-6. PMid:18364200.

[39] Benito Orejas JI, Silva Rico JC. Hearing loss. Identification and early intervention. Pediatria Integral. 2017;21:418-28.

[40] Berger R, Goeze A, Müller-Mazzotta J, Hanschmann H, Kadaifciu B, Eroglu E. Early diagnosis of infant hearing impairment after introduction of newborn hearing screening (UNHS). Laryngorhinootologie. 2012;91(10):637-40. https://doi.org/10.1055/s-0032-1316321. PMid:22773399.

[41] Bertoldi PM, Manfredi AKS, Mitre EI. Análise dos resultados da triagem auditiva neonatal no município de Batatais. Medicina (Ribeiräo Preto) 2017;50:150-7.

[42] Bevilacqua MC, Alvarenga Kde F, Costa OA, Moret AL. The universal newborn hearing screening in Brazil: from identification to intervention. Int J Pediatr Otorhinolaryngol. 2010;74(5):510-5. https://doi.org/10.1016/j.ijporl.2010.02.009. PMid:20303604.

[43] Bhalot L, Gupta Y, Kumar M, George SP, Godha S, Mundra RK. Effect of Neonatal Hearing Screening on Early Diagnosis of Sensorineural Hearing Loss. Indian J Otolaryngol Head Neck Surg. 2023;75(Suppl 1):809-14. https://doi.org/10.1007/s12070-022-03395-5. PMid:37206810.

[44] Bhatia P, Mintz S, Hecht BF, Deavenport A, Kuo AA. Early identification of young children with hearing loss in federally qualified health centers. J Dev Behav Pediatr. 2013;34(1):15-21. https://doi.org/10.1097/DBP.0b013e318279899c. PMid:23275054.

[45] Biaggio EPV, Bertuol B, David ALM, Costa LD, Melo Ã. Newborn hearing screening program: an outlook of detection to the intervention of hearing loss. Int Arch Otorhinolaryngol. 2015;19:S36.

[46] Bianchin G, Palma S, Polizzi V, Kaleci S, Stagi P, Cappai M, et al. A regional-based newborn hearing screening program: the Emilia-Romagna model after ten years of legislation. Ann Ig. 2022;35(3):297-307. http://doi.org/10.7416/ai.2022.2539. PMid:35861691.

[47] Biscegli TS, Bertoni A, Belucio IS, Marcos JMO, Matias TY, Cid FB. Triagem auditiva neonatal: estudo dos neonatos de um hospital-escola do interior do estado de São Paulo. Pediatr Mod. 2015;51(6).

[48] Bishnoi R, Baghel S, Agarwal S, Sharma S. Newborn Hearing Screening: time to Act! Indian J Otolaryngol Head Neck Surg. 2019;71(Suppl 2):1296-9. https://doi.org/10.1007/s12070-018-1352-1. PMid:31750168.

[49] Bonelli A, Marcarini L, Fontanella WM, Passerini S, Colombo F, Blotta P. Otoacustic emissions (TEOAEs) as a screening for neonatal sensorineural hearing loss: our experience. Otorinolaringologia Pediatrica. 2000;11:39-44.

[50] Botasso KD, Lima M, Correa CRS. Analysis of an outpatient child hearing health program: from screening to referral for rehabilitation. CODAS 2022;34(4):e20200403. https://doi.org/10.1590/2317-1782/20212020403.

[51] Botasso KC, Lima MCMP, Correa CRS. Análise de um programa de saúde auditiva infantil ambulatorial: da triagem ao encaminhamento para reabilitação. CoDAS. 2022;34(4):e20200403. https://doi.org/10.1590/2317-1782/20212020403. PMid:35137893.

[52] Botelho FA, Bouzada MC, Resende LM, Silva CF, Oliveira EA. Prevalence of hearing impairment in children at risk. Braz J Otorhinolaryngol. 2010;76(6):739-44. https://doi.org/10.1590/S1808-86942010000600012. PMid:21180942.

[53] Bradford BC, Baudin J, Conway MJ, Hazell JW, Stewart AL, Reynolds EO. Identification of sensory neural hearing loss in very preterm infants by brainstem auditory evoked potentials. Arch Dis Child. 1985;60(2):105-9. https://doi.org/10.1136/adc.60.2.105. PMid:4038866.

[54] Bradley M, Barbera JL. Hearing & Children: early detection-Only the beginning. Hear J. 2001;54(7):56. https://doi.org/10.1097/01.HJ.0000294113.60246.3c.

[55] Bravo AR, Krefft MM, Gómez YF, García TMF, Sandoval VP, Torrente AM. Indicadores de calidad del Programa de Detección Precoz de Hipoacusia Permanente del Hospital Padre Hurtado. Rev Otorrinolaringol Cir Cabeza Cuello. 2017;77(2):117-23. https://doi.org/10.4067/S0718-48162017000200001.

[56] Brockow I, Kummer P, Liebl B, Nennstiel-Ratzel U. Universal Newborn Hearing Screening (UNHS) - Is it possible to successfully implement it nationwide? Gesundheitswesen. 2011;73(8-9):477-82. https://doi.org/10.1055/s-0031-1285900. PMid:21887659.

[57] Brockow I, Kummer P, Liebl B, Nennstiel-Ratzel U. Universal newborn hearing screening (UNHS): is it possible to successfully implement it nationwide?. Gesundheitswesen 2011;73(08/09):477-82. https://doi.org/10.1055/s-0031-1285900.

[58] Bubbico L, Tognola G, Grandori F. Evolution of Italian universal newborn hearing screening programs. Ann Ig. 2017;29(2):116-22. https://doi.org/10.7416/ai.2017.2138. PMid:28244580.

[59] Bubbico L, Tognola G, Greco A, Grandori F. Universal newborn hearing screening programs in Italy: survey of year 2006. Acta Otolaryngol. 2008;128(12):1329-36. https://doi.org/10.1080/00016480802008165. PMid:18607902.

[60] Buser K, Bietendüwel A, Krauth C, Jalilvand N, Meyer S, Reuter G, et al. Model project of hearing screening in new-born in Hanover (preliminary results). Gesundheitswesen. 2003;65(3):200-3. https://doi.org/10.1055/s-2003-38515. PMid:12698391.

[61] Calcutt TL, Dornan D, Beswick R, Tudehope DI. Newborn hearing screening in Queensland 2009–2011: comparison of hearing screening and diagnostic audiological assessment between term and preterm infants. J Paediatr Child Health. 2016;52(11):995-1003. https://doi.org/10.1111/jpc.13281. PMid:27521761.

[62] Calevo MG, Mezzano P, Zullino E, Padovani P, Serra G, Zaccagnino V, et al. Ligurian experience on neonatal hearing screening: clinical and epidemiological aspects. Acta Paediatr. 2007;96(11):1592-9. https://doi.org/10.1111/j.1651-2227.2007.00475.x. PMid:17937684.

[63] Campos Banãles ME, López Campos D, Pérez B, López Aguado D. Correlation between otoacoustic emissions and auditory brainstem response. The relevance of using them in combination. Acta Otorrinolaringologica Espanola. 2003;54:667-70. https://doi.org/10.1016/S0001-6519(03)78465-9.

[64] Campos ME, López Campos D, Pérez B, López Aguado D. Correlation between otoacoustic emissions and BAEP. The importance of their combined use]. Acta Otorrinolaringol Esp 2003;54(10):667-70.

[65] Canale A, Favero E, Lacilla M, Recchia E, Schindler A, Roggero N, et al. Age at diagnosis of deaf babies: a retrospective analysis highlighting the advantage of newborn hearing screening. Int J Pediatr Otorhinolaryngol. 2006;70(7):1283-9. https://doi.org/10.1016/j.ijporl.2006.01.008. PMid:16488484.

[66] Cao-Nguyen MH, Kos MI, Guyot JP. Benefits and costs of universal hearing screening programme. Int J Pediatr Otorhinolaryngol. 2007;71(10):1591-5. https://doi.org/10.1016/j.ijporl.2007.07.008. PMid:17719096.

[67] Cardoso LF, Bissani C, Pinheiro MMC, Viveiros CM, Carreirão W Fo. Análise da implantação de programa de triagem auditiva neonatal em um hospital universitário. Rev Bras Otorrinolaringol. 2009;75(2):237-44. https://doi.org/10.1590/S0034-72992009000200013.

[68] Cassidy JW, Ditty KM. Gender differences among newborns on a transient otoacoustic emissions test for hearing. J Music Ther. 2001;38(1):28-35. https://doi.org/10.1093/jmt/38.1.28. PMid:11407963.

[69] Ceccato C, Chirico G, Mastricci N, Precerutti G, Rondini G. DPOAE screening on discharging infants in local NICU. In: McCafferty WG, editor. Sydney ’97: XVI World Congress of Otorhinolaryngology Head and Neck Surgery; 1997 Mar 2-7; Sydney, Australia. Bologna: Monduzzi Editore; 1997. p. 21-5.

[70] Černý M, Zoban P, Groh D, Brabec R, Vejvalka J, Kabelka Z, et al. Screening for hearing impairment in newborns using transient evoked otoacoustic emissions. Ceskoslov Pediatr. 2003;58:700-4.

[71] Chadha S, Bais AS. Auditory brainstem responses in high risk and normal newborns. Indian J Pediatr. 1997;64(6):777-84. https://doi.org/10.1007/BF02725499. PMid:10771920.

[72] Champion S. Assessment of hearing in high risk infants, using brainstem evoked response audiometry. Indian J Otolaryngol Head Neck Surg. 2021;73(3):383-8. https://doi.org/10.1007/s12070-020-02363-1. PMid:34471628.

[73] Chang HW, Dillon H, Carter L, van Dun B, Young ST. The relationship between cortical auditory evoked potential (CAEP) detection and estimated audibility in infants with sensorineural hearing loss. Int J Audiol. 2012;51(9):663-70. https://doi.org/10.3109/14992027.2012.690076. PMid:22873205.

[74] Chen G, Yi X, Chen P, Dong J, Yang G, Fu S. A large-scale newborn hearing screening in rural areas in China. Int J Pediatr Otorhinolaryngol. 2012;76(12):1771-4. https://doi.org/10.1016/j.ijporl.2012.08.021. PMid:22954384.

[75] Chen X, Yuan M, Lu J, Zhang Q, Sun M, Chang F. Assessment of universal newborn hearing screening and intervention in Shanghai, China. Int J Technol Assess Health Care. 2017;33(2):206-14. https://doi.org/10.1017/S0266462317000344. PMid:28583223.

[76] Chibisova SS, Tsygankova ER, Markova TG, Rumiantseva MG. The universal audiological screening of newborn infants: achievements and challenges. Vestn Otorinolaringol. 2014;(2):49-53. PMid:24781172.

[77] Chiriboga LF, Sideri KP, Ferraresi Rodrigues Figueiredo SN, Monteiro Pinto ES, Chiriboga Arteta LM. Outcomes of a universal neonatal hearing screening program of 9941 newborns over a one-year period in Campinas, Brazil. Int J Pediatr Otorhinolaryngol. 2021;148:110839. https://doi.org/10.1016/j.ijporl.2021.110839. PMid:34274888.

[78] Choi KY, Park SK, Choi S, Chang J. Analysis of Newborn Hearing Screening Results in South Korea after National Health Insurance Coverage: A Nationwide Population-Based Study. Int J Environ Res Public Health. 2022;19(22):15052. https://doi.org/10.3390/ijerph192215052. PMid:36429776.

[79] Chu CW, Chen YJ, Lee YH, Jaung SJ, Lee FP, Huang HM. Government-funded universal newborn hearing screening and genetic analyses of deafness predisposing genes in Taiwan. Int J Pediatr Otorhinolaryngol. 2015;79(4):584-90. https://doi.org/10.1016/j.ijporl.2015.01.033. PMid:25724631.

[80] Chung YS, Oh SH, Park SK. Results of a government-supported newborn hearing screening pilot project in the 17 cities and provinces from 2014 to 2018 in Korea. J Korean Med Sci. 2020;35(31):e251. https://doi.org/10.3346/jkms.2020.35.e251. PMid:32776720.

[81] Chung YS, Oh SH, Park SK. Results of a government-supported newborn hearing screening pilot project in the 17 cities and provinces from 2014 to 2018 in Korea. J Korean Med Sci. 2020;35(31):e251. https://doi.org/10.3346/jkms.2020.35.e251. PMid:32776720.

[82] Cianfrone F, Mammarella F, Ralli M, Evetovic V, Pianura CM, Bellocchi G. Universal newborn hearing screening using A-TEOAE and A-ABR: the experience of a large public hospital. J Neonatal Perinatal Med. 2018;11(1):87-92. https://doi.org/10.3233/NPM-181744. PMid:29689750.

[83] Cianfrone F, Ubah F, Cazzaniga C, Abuhajar S, Ralli M, Angeletti D, et al. Evaluation of newborn hearing screening program in aSl roma 1, rome, italy. Otorhinolaryngology. 2021;71(2):95-9. https://doi.org/10.23736/S2724-6302.20.02335-8.

[84] Colella-Santos MF, Sartorato EL, Tazinazzio TG, de Françozo MCF, do Couto CM, Castilho AM, et al. An auditory health program for neonates in ICU and/or intermediate care settings. Braz J Otorhinolaryngol. 2013;79(6):709-15. https://doi.org/10.5935/1808-8694.20130130. PMid:24474482.

[85] Colella-Santos MF, Sartorato EL, Tazinazzio TG, Françozo MFC, Couto CM, Castilho AM, et al. Programa de saude auditiva em neonatos que permaneceram em UTI e/ou cuidados intermediarios. Braz j Otorhinolaryngol (Impr). 2013;79(6):709-15. https://doi.org/10.5935/1808-8694.20130130.

[86] Coll J, Régnier C. Early auditory potentials in the detection of deafness. Rev Laryngol Otol Rhinol (Bord). 1988;109(4):325-6. PMid:3238194.

[87] Collins A, Beswick R, Driscoll C, Kei J. Clinical characteristics of infants identified with a conductive hearing loss through universal newborn hearing screening: a population-based sample. Int J Pediatr Otorhinolaryngol. 2022;161:111268. https://doi.org/10.1016/j.ijporl.2022.111268. PMid:35964490.

[88] Costa BM, Grawer RS, Soldera CLC, Machado MS, Benvenutti SK. Newborn hearing screening in philanthropic hospital in porto alegre. Int Arch Otorhinolaryngol. 2015;19:S93.

[89] Cox LC, Toro MR. Evolution of a universal infant hearing screening program in an inner city hospital. Int J Pediatr Otorhinolaryngol. 2001;59(2):99-104. https://doi.org/10.1016/S0165-5876(01)00462-1. PMid:11378184.

[90] Cundall J. Universal newborn hearing screening in Tennessee. Tennessee Medicine : Journal of the Tennessee Medical Association. 1997;90(9):370-1. PMid:9308393.

[91] Curcin OA, Sitka U, Rasinski C. Results of screening Very Low Birthweight (VLBW) infants’ hearing. Pediatrics and Related Topics. 1998;36:469-79.

[92] Curnock DA. Identifying hearing impairment in infants and young children. BMJ. 1993;307(6914):1225-6. https://doi.org/10.1136/bmj.307.6914.1225. PMid:8281050.

[93] Da Silva LS, Ribeiro GE, Da Silva DPC, Montovani JC. Outcomes of automated auditory evoked potential realized in different places and factors associated with refer cases. Int Arch Otorhinolaryngol. 2017;21:S104.

[94] Dalzell LE, Orlando M, MacDonald M, Berg A, Bradley M, Cacace A, et al. The New York State universal newborn hearing screening demonstration project: ages of hearing loss identification, hearing aid fitting, and enrollment in early intervention. Ear Hear. 2000;21(2):118-30. https://doi.org/10.1097/00003446-200004000-00006. PMid:10777019.

[95] Dantas MB, Anjos CA, Camboim ED, Pimentel Mde C. Results of a neonatal hearing screening program in Maceió. Braz J Otorhinolaryngol. 2009;75(1):58-63. https://doi.org/10.1016/S1808-8694(15)30832-6. PMid:19488561.

[96] Davis A, Hind S. The newborn hearing screening programme in England. Int J Pediatr Otorhinolaryngol. 2003;67(Suppl 1):S193-6. https://doi.org/10.1016/j.ijporl.2003.08.024. PMid:14662193.

[97] Daykhes NA, Grigor’eva EA, Nazarochkin YV, Davydov VM, Kuznetsov AO. The results of universal audiological screening of newborn infants in the Astrakhan region. Vestn Otorinolaringol 2017;82(2):16-8. https://doi.org/10.17116/otorino201782216-18.

[98] De Barros Boishardy A, Lenoir FM, Brami P, Kapella M, Obstoy MF, Amstutz-Montadert I, et al. Universal hearing screening: 10,835 newborns tested in maternity wards of the geographical Department of Eure, France. Annales d’oto-Laryngologie et de Chirurgie Cervico Faciale : Bulletin de La Société d’oto-Laryngologie. Hop Paris. 2005;122(5):223-30. https://doi.org/10.1016/S0003-438X(05)82353-9. PMid:16439932.

[99] De González Dios J, Mollar Maseres J. Universal screening for neonatal hearing loss: an evaluation of the program as opposed to an evaluation of the test. Acta Otorrinolaringologica Espanola. 2005;56:331-4. https://doi.org/10.1016/S0001-6519(05)78625-8.

[100] De Leenheer EM, Janssens S, Padalko E, Loose D, Leroy BP, Dhooge IJ. Etiological diagnosis in the hearing impaired newborn: proposal of a flow chart. Int J Pediatr Otorhinolaryngol. 2011;75(1):27-32. https://doi.org/10.1016/j.ijporl.2010.05.040. PMid:21047691.

[101] De Mattos WM, Cardoso LF, Bissani C, Pinheiro MMC, Viveiros CM, Carreirão Filho W. Newborn hearing screening program implantation analysis at a university hospital. Braz J Otorhinolaryngol. 2009;75(2):237-44. https://doi.org/10.1016/S1808-8694(15)30784-9. PMid:19575110.

[102] Delgado Domínguez JJ. Early detection of hearing loss in children. Pediatria de Atencion Primaria. 2011;13:279-97.

[103] Deng X, Ema S, Mason C, Nash A, Carbone E, Gaffney M. Receipt and timeliness of newborn hearing screening and diagnostic services among babies born in 2017 in 9 states. J Public Health Manag Pract. 2022;28(1):E100-8. https://doi.org/10.1097/PHH.0000000000001232. PMid:32956290.

[104] Desloovere C, Lemmens W, Feenstra L, Debruyne F, Van Kerschaver E, Offeciers FE, et al. Universal hearing screening of newborns in Flanders: from dreaming to reality? (multiple letters). Tijdschr Geneeskd. 2000;56:1203. https://doi.org/10.2143/TVG.56.16.1000836.

[105] Dlouhá O, Černý L, Hrdličková M, Jedlička I, Puchmajer P, Vohradník M. Auditory screening in high-risk infants. Otorinolaryngologie a Foniatrie. 2002;51:4-7.

[106] Downs MP. Universal newborn hearing screening - The Colorado story. Int J Pediatr Otorhinolaryngol. 1995;32(3):257-9. https://doi.org/10.1016/0165-5876(95)01183-C. PMid:7665273.

[107] Duci AR, Pons VA, Porta CL, Moya BA, Salomón GJ, Martínez CH, et al. Detección universal de hipoacusias en recién nacidos. Rev Otorrinolaringol Cir Cabeza Cuello. 2000;60:143-50.

[108] Durante AS, Carvallo RM, da Costa FS, Soares JC. Characteristics of transient evoked otoacoustic emissions in newborn hearing screening program. Pró-Fono R Atual Cient. 2005;17(2):133-40. https://doi.org/10.1590/s0104-56872005000200002.

[109] Durante AS, Carvallo RMM, Costa MTZ, Ianciarullo MA, Voegels RL, Takahashi GM, et al. A implementação de programa de triagen auditiva neonatal universal em um hospital universitário brasileiro. Pediatria (Säo Paulo). 2004;26:78-84.

[110] Eibenstein A, Varakliotis T, Saccoccio A, Cisternino S, La Gamma R, Mattei A, et al. Newborn hearing screening with TEOAE: our experience on 4759 infants. Otorinolaringologia. 2014;64:27-32.

[111] Eiserman WD, Hartel DM, Shisler L, Buhrmann J, White KR, Foust T. Using otoacoustic emissions to screen for hearing loss in early childhood care settings. Int J Pediatr Otorhinolaryngol. 2008;72(4):475-82. https://doi.org/10.1016/j.ijporl.2007.12.006. PMid:18276019.

[112] Elpers J, Lester C, Shinn JB, Bush ML. Rural family perspectives and experiences with early infant hearing detection and intervention: a qualitative study. J Community Health. 2016;41(2):226-33. https://doi.org/10.1007/s10900-015-0086-1. PMid:26316007.

[113] Farhat AS, Ghasemi MM, Akhondian J, Mohamadzadeh A, Esmaeili H, Amiri R, et al. Assessment of the prevalence of hearing impairment in neonates born in Imam Reza, Ghaem and OM-Albanin Hospitals of Mashhad. Iran J Nematol. 2014;5:17-20.

[114] Felden GQP, Didoné DD, Sleifer P, Gomes E, Etcheverria SK, França MCT, et al. Auditory monitoring in children with risk indicator for hearing loss. Int Arch Otorhinolaryngol. 2017;21:S8.

[115] Fernandes JC, Nozawa MR. Estudo da efetividade de um programa de triagem auditiva neonatal universal. Cien Saude Colet. 2010;15(2):353-61. https://doi.org/10.1590/S1413-81232010000200010.

[116] Fernandez Martinez MD, Bosch Gimenez VM, Recuero Fernandez E, Vicente Santos T, Borrajo Guadarrama E, Canteras Jordana M. Evaluation of audition in children who had a birth weight less than 1,201 grams. An Esp Pediatr. 1995;43:184-6.

[117] Feroz S, Irshad M, Shamsi SA, Sohail E, Hamid A, Rohan M. Evaluation of optimal postnatal timing for the screening of neonatal hearing via transient evoked oto-acoustic emission (TEOAE): a retrospective cross-sectional study. Ir J Med Sci. 2025;95(2):961-8. https://doi.org/10.1007/s11845-025-04238-2. PMid:41432879.

[118] Ferro LM, Tanner G, Erler SF, Erickson K, Dhar S. Comparison of universal newborn hearing screening programs in Illinois hospitals. Int J Pediatr Otorhinolaryngol. 2007;71(2):217-30. https://doi.org/10.1016/j.ijporl.2006.10.004. PMid:17097746.

[119] Findlen UM, Malhotra PS, Hunter LL. Early hearing detection. Clin Perinatol. 2025;52(3):509-21. https://doi.org/10.1016/j.clp.2025.06.009. PMid:40850713.

[120] Fink N, Goeze A, Zaretsky E, Fink A, Reimann K, Hey C. Follow-up II of newborn hearing screening: evaluation of a follow-up II facility after implementation of newborn hearing screening in Germany. HNO. 2022;70(3):179-86. https://doi.org/10.1007/s00106-021-01098-x. PMid:34448878.

[121] Fitzpatrick EM, Durieux-Smith A, Whittingham J. Clinical practice for children with mild bilateral and unilateral hearing loss. Ear Hear. 2010;31(3):392-400. https://doi.org/10.1097/AUD.0b013e3181cdb2b9. PMid:20054278.

[122] François M, Hautefort C, Nasra Y, Zohoun S. Evolution of age at diagnosis of congenital hearing impairment. Eur Ann Otorhinolaryngol Head Neck Dis. 2011;128(2):59-63. https://doi.org/10.1016/j.anorl.2010.10.009. PMid:21295536.

[123] Fujikawa S, Yoshinaga-Itano C. Current status of universal newborn hearing screening. Curr Opin Otolaryngol Head Neck Surg. 2000;8(5):404-8. https://doi.org/10.1097/00020840-200010000-00009.

[124] Fukushima K, Mimaki N, Fukuda S, Nishizaki K. Pilot study of universal newborn hearing screening in Japan: district-based screening program in Okayama. Ann Otol Rhinol Laryngol. 2008;117(3):166-71. https://doi.org/10.1177/000348940811700302. PMid:18444475.

[125] Gáborján A, Götze J, Küstel J, Kecskeméti N, Baranyi I, Csontos F, et al. Verification results of objective newborn hearing screening. Orv Hetil. 2019;160(47):1850-5. https://doi.org/10.1556/650.2019.31604. PMid:31736348.

[126] Gaborján A, Katona G, Szabó M, Muzsik B, Kustel M, Horváth M, et al. Universal newborn hearing screening with automated auditory brainstem response (AABR) in Hungary: 5-year experience in diagnostics and influence on the early intervention. Eur Arch Oto-Rhino-Laryngol. 2022;279(12):5647-54. https://doi.org/10.1007/s00405-022-07441-4. PMid:35767058.

[127] Galhotra A, Sahu P. Challenges and solutions in implementing hearing screening program in India. Indian J Community Med. 2019;44(4):299-302. https://doi.org/10.4103/ijcm.IJCM_73_19. PMid:31802788.

[128] Garbaruk ES, Fedorova LA, Savenko IV, Vikhnina SM, Boboshko MY. Childhood hearing screening: achievements, difficulties, and possible ways to improve. Vestn Otorinolaringol. 2021;86(1):82-9. https://doi.org/10.17116/otorino20218601182. PMid:33720658.

[129] Gavilan Cellie I. Auditory screening of newborns: new procedures. Rev Laryngol Otol Rhinol (Bord). 1993;114:239-41. PMid:8029542.

[130] Geal-Dor M, Levi H, Elidan Y, Arad I. The hearing screening program for newborns with otoacoustic emission for early detection of hearing loss. Harefuah. 2002;141(7):586-90, 668. PMid:12187552.

[131] Gellrich D, Gröger M, Echternach M, Eder K, Huber P. Neonatal hearing screening - does failure in TEOAE screening matter when the AABR test is passed? Eur Arch Oto-Rhino-Laryngol. 2024;281(3):1273-83. https://doi.org/10.1007/s00405-023-08250-z. PMid:37831131.

[132] Gentiletti-Faenze GG, Yañez-Suarez O, Cornejo-Crus JM. Evaluation of automatic identification algorithms for auditory brainstem response used in universal hearing loss *screening.* In: Proceedings of the 25th Annual International Conference of the IEEE Engineering in Medicine and Biology Society (IEEE Cat. No.03CH37439); 2003; Cancun, Mexico. USA: IEEE; 2003. vol. 3, p. 2857-60. https://doi.org/10.1109/IEMBS.2003.1280514.

[133] Georgescu M, Vladareanu S. Early audiological diagnostic in newborn and infant. GinecoEu. 2015;11(3):131-3. https://doi.org/10.18643/gieu.2015.131.

[134] Giordano T, Marchegiani AM, Germiller JA. Children With Sensorineural Hearing Loss And Referral To Early Intervention. ORL Head Neck Nurs. 2015;33(3):10-4. PMid:26427185.

[135] Godoy BC, Bustamante ML. Evaluación de la fase de screening auditivo en menores con factores de riesgo. Rev Otorrinolaringol Cir Cabeza Cuello. 2006;66(2):103-6. https://doi.org/10.4067/S0718-48162006000200005.

[136] González De Aledo Linos A, Bonilla Miera C, Morales Angulo C, Gómez Da Casa F, Barrasa Benito J. Universal newborn hearing screening in Cantabria (Spain): results of the first two years. Anales de Pediatria. 2005;62(2):135-40. https://doi.org/10.1157/13071310. PMid:15701309.

[137] González de Dios J, Mollar Maseres J, Rebagliato Russo M. Evaluation of a universal screening program for hypacusia in neonates. An Pediatr (Barc) 2005;63(3):230-7. https://doi.org/10.1157/13078486.

[138] González Díaz CA, Chapa González C, Laciar Leber E, Vélez HA, Puente NP, Flores D-L, et al., editors. VIII Latin American Conference on Biomedical Engineering and XLII National Conference on Biomedical Engineering. In: Proceedings of CLAIB-CNIB 2019; 2019 Oct 2-5; Cancún, México. Cham: Springer International Publishing; 2020. vol. 75. https://doi.org/10.1007/978-3-030-30648-9.

[139] Gonzalez LR, Díaz ALZ, Mora OC. Effectiveness of “Universal Newborn Hearing Screening program” of Caja Costarricense del Seguro Social, in detecting deafness in children, from 2016 to 2018. Poblac Salud Mesoam. 2022;19(2):204-23. http://dx.doi.org/10.15517/psm.v0i19.47144.

[140] González L, Fernández de Soto JM, Torres MI. Current status of the programs for detection of hearing loss in children younger than six months in Cali. Colomb Med (Cali). 2012;43(1):73-81. https://doi.org/10.25100/cm.v43i1.1061.

[141] Gordo A, Parlato EM, de Azevedo MF, Guedes ZCF. Triagem auditiva em bebês de 2 a 12 meses. Pro Fono. 1994;6:7-13.

[142] Granell J, Martin G. Early detection and intervention on infant hearing loss. In: Dupont JP, editor. Hearing loss: classification, causes and treatment. New York: Nova Science Publishers; 2011. p. 189-212.

[143] Greczka G, Wróbel M, Dąbrowski P, Mikołajczak K, Szyfter W. Universal Neonatal Hearing Screening Program in Poland–10-year summary. Otolaryngol Pol. 2015;69(3):1-5. https://doi.org/10.5604/00306657.1156325. PMid:26388243.

[144] Greczka G, Zych M, Szyfter W, Wróbel M. Analysis of changes in the Polish Universal Neonatal Hearing Screening Program over 15 years. Otolaryngol Pol. 2018;72(2):13-9. https://doi.org/10.5604/01.3001.0011.7247. PMid:29748454.

[145] Grégoire J. Results of a deafness screening program for infants: a pilot project. Can J Public Health. 1996;87(5):339-42. PMid:8991754.

[146] Greville KA, Keith WJ. The effectiveness of two infant hearing screening programmes in New Zealand. Scand Audiol. 1978;7(3):139-45. https://doi.org/10.3109/01050397809076280. PMid:756078.

[147] Groh D, Kabelka Z, Jurovcik M, Koprivova H, Zoban P, Cerny M. Results of a screening programme of hearing disorders in neonates in the Faculty Hospital Prague - Motol in 1997 - 1998. Otorinolaryngologie a Foniatrie. 1999;48:3-6.

[148] Guastini L, Mora R, Dellepiane M, Santomauro V, Mora M, Rocca A, et al. Evaluation of an automated auditory brainstem response in a multi-stage infant hearing screening. Eur Arch Oto-Rhino-Laryngol. 2010;267(8):1199-205. https://doi.org/10.1007/s00405-010-1209-z. PMid:20148257.

[149] Guilhoto LMFF, Quintal VS, Da Costa MTZ. Brainstem auditory evoked response in normal term neonates. Arq Neuropsiquiatr. 2003;61(4):906-8. https://doi.org/10.1590/S0004-282X2003000600003. PMid:14762588.

[150] Guimarães VC, Barbosa MA. Prevalência de alterações auditivas em recém-nascidos em hospital escola. Int Arch Otorhinolaryngol. 2012;16:179-85. https://doi.org/10.7162/S1809-97772012000200005.

[151] Gulati A, Sakthivel P, Singh I, Ramji S. The Hearing Status of Preterm Infant’s ≤ 34 Weeks as Revealed by Otoacoustic Emissions (OAE) Screening and Diagnostic Brainstem Evoked Response Audiometry (BERA): A Tertiary Center Experience. Indian J Otolaryngol Head Neck Surg. 2022;74(Suppl 1):178-83. https://doi.org/10.1007/s12070-020-01945-3. PMid:36032856.

[152] Halder AL, Mollah MAH, Baki MA, Khan S, Nahar J. Hearing impairment among the high-risk neonates: findings from a hospital-based targeted screening. Mymensingh Med J. 2025;34(2):400-7. PMid:40160057.

[153] Hall V, Brosch S, Hoffmann TK. Evaluation of universal newborn hearing screening and follow-up. Laryngorhinootologie. 2020;99(5):299-307. https://doi.org/10.1055/a-1114-6452. PMid:32131107.

[154] Hanschmann H, Berger R. Universal newborn hearing screening. New early recognition examination. Monatsschr Kinderheilkd. 2010;158(6):597-607. https://doi.org/10.1007/s00112-010-2203-7.

[155] Harrison WA, Dunnell JJ, Mascher K, Fletcher K, Vohr BR, Gorga MP, et al. Identification of Neonatal Hearing Impairment: experimental protocol and database management. Ear Hear. 2000;21(5):357-72. https://doi.org/10.1097/00003446-200010000-00004. PMid:11059698.

[156] Hatzopoulos S, Pelosi G, Petruccelli J, Rossi M, Vigi V, Chierici R, et al. Efficient otoacoustic emission protocols employed in a hospital-based neonatal screening program. Acta Otolaryngol. 2001;121(2):269-73. https://doi.org/10.1080/000164801300043802. PMid:11349794.

[157] Hayes D. Current status of universal newborn hearing screening. Curr Opin Otolaryngol Head Neck Surg. 2002;10(5):382-6. https://doi.org/10.1097/00020840-200210000-00010.

[158] Helge T, Werle E, Barnick M, Wegner C, Rühe B, Aust G, et al. Two-tier screening process (TEOAE/AABR) reduces recall rates in newborn hearing screening. HNO. 2005;53(7):655-60. https://doi.org/10.1007/s00106-004-1124-y. PMid:15565423.

[159] Hergils L. How do we identify hearing impairment in early childhood?. Acta Paediatr Suppl. 2000;89(434):12-6. https://doi.org/10.1111/j.1651-2227.2000.tb03090.x. PMid:11055312.

[160] Hoffmann A, Deuster D, Rosslau K, Knief A, Am Zehnhoff-Dinnesen A, Schmidt CM. Feasibility of 1000Hz tympanometry in infants: tympanometric trace classification and choice of probe tone in relation to age. Int J Pediatr Otorhinolaryngol. 2013;77(7):1198-203. https://doi.org/10.1016/j.ijporl.2013.05.001. PMid:23726955.

[161] Hosforddunn H, Johnson S, Simmons FB, Malachowski N, Low K. Infant hearing screening - program implementation and validation. Ear Hear. 1987;8(1):12-20. https://doi.org/10.1097/00003446-198702000-00003. PMid:3549402.

[162] Huang CM, Yang IY, Julie Ma YC, Lin GSF, Yang CC, Tsai HT, et al. The effectiveness of the promotion of newborn hearing screening in Taiwan. Int J Pediatr Otorhinolaryngol. 2014;78(1):14-8. https://doi.org/10.1016/j.ijporl.2013.10.005. PMid:24300945.

[163] Huang EY, DeSell M, White AD, Walsh J, Jenks CM. Results and patient satisfaction from an early access infant hearing detection clinic. Int J Pediatr Otorhinolaryngol. 2023;164:111396. https://doi.org/10.1016/j.ijporl.2022.111396. PMid:36450185.

[164] Huang L, Cai Z, Zhang H, Peng S, Wu D, Wang L, et al. [Study on multi-area universal newborn hearing screening in countryside of China]. Lin Chuang Er Bi Yan Hou Tou Jing Wai Ke Za Zhi. 2009;23(16):737-42. PMid:20041607.

[165] Huang L, Xiong F, Li J, Yang F. An analysis of hearing screening test results in 2291 premature infants of Chinese population. Int J Pediatr Otorhinolaryngol. 2017;95:15-9. https://doi.org/10.1016/j.ijporl.2017.01.027. PMid:28576525.

[166] Huarte Irujo A. Evaluación audiológica de la población infantil. Rev Chil Fonoaudiol 2003;4:17-35. https://doi.org/10.5354/0719-4692.2003.56613.

[167] Irfan M, Shuaib M, Imran M, Ahmed T, Saleem S, Khan HA. Outcomes of Universal Neonatal Hearing Screening Program in a Tertiary Care Hospital in Lahore. Ann Abbasi Shaheed Hosp Karachi Med Dent Coll. 2024;29(3):270-6. https://doi.org/10.58397/ashkmdc.v29i3.890.

[168] Iwanicka-Pronicka K, Radziszewska-Konopka M, Wybranowska A, Churawski Ł. Analysis of specificity and sensitivity of Polish “Universal Newborn Hearing Screening Program.”. Otolaryngol Pol. 2008;62(1):88-95. https://doi.org/10.1016/S0030-6657(08)70215-4. PMid:18637428.

[169] Izumets O, Nezhivenko T, Murashko T. Hearing loss among neonates, early diagnostics. Likars’ka Sprava. 2015:172-4.

[170] Jacob J, Kurien M, Kumar P, Krishnan L. Challenges of universal newborn hearing screening in a developing country-a double-edged sword. Indian J Otolaryngol Head Neck Surg. 2022;74(Suppl 1):395-401. https://doi.org/10.1007/s12070-020-02170-8. PMid:36032816.

[171] Jacobson CA, Jacobson JT. Follow-up services in newborn hearing screening programs. J Am Acad Audiol. 1990;1(4):181-6. PMid:2132602.

[172] Jacobson GP. Universal newborn hearing loss: screening, identification, intervention. Am J Audiol. 2001;10(2):52. https://doi.org/10.1044/1059-0889(2001/ed02). PMid:11808719.

[173] Jakubíková J, Kabátová Z, Závodná M. Identification of hearing loss in newborns by transient otoacoustic emissions. Int J Pediatr Otorhinolaryngol. 2003;67(1):15-8. https://doi.org/10.1016/S0165-5876(02)00285-9. PMid:12560144.

[174] Jakubikova J, Zavodna M, Kardosova A, Vicianova K. Auditory screening of neonates using an objective method–otoacoustic emissions. Bratisl Lek Listy. 1999;100(11):607-10. PMid:10758739.

[175] Januário GC, Lemos SMA, de Lima Friche AA, Alves CRL. Quality indicators in a newborn hearing screening service. Braz J Otorhinolaryngol. 2015;81(3):255-63. https://doi.org/10.1016/j.bjorl.2014.08.008. PMid:25596650.

[176] Jayne A, Fullante PB, Karisse T, Dariel A, Lenon M, Sunga MB, et al. Pilot Implementation of a Community-based, eHealth-enabled Service Delivery Model for Newborn Hearing Screening and Intervention in the Philippines. Acta Med Philipp. 2023;57(9):73-84. https://doi.org/10.47895/amp.v57i9.5332. PMid:39483802.

[177] Johnson JL, White KR, Widen JE, Gravel JS, Vohr BR, James M, et al. A multisite study to examine the efficacy of the otoacoustic emission/automated auditory brainstem response newborn hearing screening protocol: introduction and overview of the study. Am J Audiol. 2005;14(2):S178-85. https://doi.org/10.1044/1059-0889(2005/020). PMid:16489862.

[178] Kanji A, Khoza-Shangase K. The occurrence of high-risk factors for hearing loss in very-low-birth-weight neonates: a retrospective exploratory study of targeted hearing screening. S Afr J Commun Disord. 2012;59(1):3-7. https://doi.org/10.4102/sajcd.v59i1.16. PMid:23409613.

[179] Kanji A, Krabbenhoft K. Audiological follow-up in a risk-based newborn hearing screening programme: an exploratory study of the influencing factors. S Afr J Commun Disord. 2018;65(1):e1-7. https://doi.org/10.4102/sajcd.v65i1.587. PMid:30456962.

[180] Kawata A, Kumagami H, Hara M, Sainoo Y, Takasaki K, Takahashi H, et al. Universal newborn hearing screening in nagasaki prefecture - Experience for 4. 5 years. Pract Otorhinolaryngol (Basel). 2011;104(12):849-54. https://doi.org/10.5631/jibirin.104.849.

[181] Keefe DH, Folsom RC, Gorga MP, Vohr BR, Bulen JC, Norton SJ. Identification of Neonatal Hearing Impairment: ear-canal measurements of acoustic admittance and reflectance in neonates. Ear Hear. 2000;21(5):443-61. https://doi.org/10.1097/00003446-200010000-00009. PMid:11059703.

[182] Kemper AR. Universal newborn hearing screening improves quality of life for children with permanent hearing impairment. J Pediatr. 2011;158(5):859. https://doi.org/10.1016/j.jpeds.2011.03.011. PMid:21482247.

[183] Kerschner JE, Meurer JR, Conway AE, Fleischfresser S, Cowell MH, Seeliger E, et al. Voluntary progress toward universal newborn hearing screening. Int J Pediatr Otorhinolaryngol. 2004;68(2):165-74. https://doi.org/10.1016/j.ijporl.2003.10.010. PMid:14725983.

[184] Kiese-Himmel C, Schroff J, Kruse E. Identification and diagnostic evaluation of hearing impairments in early childhood in German-speaking infants. Eur Arch Oto-Rhino-Laryngol. 1997;254(3):133-9. https://doi.org/10.1007/BF02471276. PMid:9112033.

[185] Kim JH, Lee KB, Koo YC, Hong SA, Lee Y, Son EJ. Evaluation of transient evoked otoacoustic emission in the newborn hearing screening program in neonatal intensive care unit. Korean J Audiol. 2011;15:81-4.

[186] Koester M, Nekahm-Heis D, Zorowka P. Early detection of hearing impairment in the newborn. MMW Fortschr Med. 2002;144:39-40. PMid:12577738.

[187] Konukseven O, Genc A, Muderris T, Kayikci MK, Turkyilmaz D, Ozturk B, et al. Can automated auditory brainstem response be used as an initial stage screening test in newborn hearing screening programs? J Int Adv Otol. 2010;6:231-8.

[188] Korres SG, Balatsouras DG, Nikolopoulos T, Korres GS, Ferekidis E. Making universal newborn hearing screening a success. Int J Pediatr Otorhinolaryngol. 2006;70(2):241-6. https://doi.org/10.1016/j.ijporl.2005.06.010. PMid:16029898.

[189] Korres S, Nikolopoulos TP, Peraki EE, Tsiakou M, Karakitsou M, Apostolopoulos N, et al. Outcomes and efficacy of newborn hearing screening: strengths and weaknesses (success or failure?). Laryngoscope. 2008;118(7):1253-6. https://doi.org/10.1097/MLG.0b013e31816d726c. PMid:18401271.

[190] Krauss MK, Heider CC, Sierra GM, Ribalta LG. Estrategias para mejorar el seguimiento del Programa de Evaluación Auditiva Neonatal Universal. Rev Otorrinolaringol Cir Cabeza Cuello. 2013;73(2):140-5. https://doi.org/10.4067/S0718-48162013000200005.

[191] Krishnan LA. Universal newborn hearing screening follow-up: A university clinic perspective. Am J Audiol. 2009;18(2):89-98. https://doi.org/10.1044/1059-0889(2009/09-0003). PMid:19704112.

[192] Kumar A, Gupta SC, Sinha VR. Universal hearing screening in newborns using otoacoustic emissions and brainstem evoked response in eastern Uttar Pradesh. Indian J Otolaryngol Head Neck Surg. 2017;69(3):296-9. https://doi.org/10.1007/s12070-017-1081-x. PMid:28929058.

[193] Lan H, Liu M, Huang C, Ren J, Huang Y, Jiang F, et al. Evaluation of the current situation and quality of neonatal hearing screening from hearing screening practitioners’ perspective: a cross-sectional study. J Matern Fetal Neonatal Med. 2024;37(1):2317412. https://doi.org/10.1080/14767058.2024.2317412. PMid:38369473.

[194] Langagne T, Schmidt P, Leveque M, Chays A. Universal hearing screening in the Champagne-Ardenne regions: results and consideration after 55 000 births from January 2004 to June 2007. Rev Laryngol Otol Rhinol (Bord). 2008;129(3):153-8. PMid:19694157.

[195] Lee-Ramos CM, Francis A, Collanto AS, John P, Sison LG, Chiong CM. Hearing screening through frequency analysis of auditory brainstem response using PhysioNet Data. Acta Med Philipp. 2023;57(9):32-8. https://doi.org/10.47895/amp.v57i9.4336. PMid:39483803.

[196] Leonhardt A, Müller M. The Newborn hearing-screening: setting a direction for life. Sprache Stimme Gehor. 2008;32:12-7. https://doi.org/10.1055/s-2007-1022556.

[197] Lewis DR, Racai R, Bevilacqua MC. Identificaçäo precoce de deficiência auditiva 1987:113-8.

[198] Lewis DR, Marone SAM, Mendes BCA, Cruz OLM, Nóbrega M. Comitê multiprofissional em saúde auditiva: COMUSA. Braz J Otorhinolaryngol. 2010;76:121-8.

[199] Lim SB, Daniel LM. Establishing a universal newborn hearing screening programme. Ann Acad Med Singap. 2008;37(12, Suppl):63-5. PMid:19904454.

[200] Lima GML, Marba STM, Santos MFC. Avaliação auditiva em recém-nascidos internados em unidades de terapia intensiva e de cuidados intermediários: triagem e acompanhamento ambulatorial. Rev ciênc méd (Campinas) 2005;14:147-56.

[201] Lima MCMP, Rossi TRF, Françozo MFC, Marba ST, Lima GML, Santos MFC. Detecção de perdas auditivas em neonatos de um hospital público. Rev Soc Bras Fonoaudiol. 2010;15(1):1-6. https://doi.org/10.1590/S1516-80342010000100003.

[202] Lima PT, Goldbach MG, Monteiro MC, Ribeiro MG. A triagem auditiva neonatal na Rede Municipal do Rio de Janeiro, Brasil. Ciênc Saúde Colet. 2015;20:57-63. https://doi.org/10.1590/1413-81232014201.21002013.

[203] Ling Q, Huang Z, Zhou Y, Li M, Zhong J. 3,200 cases of neonatal hearing screening results and analysis of related factors. Lin Chuang Er Bi Yan Hou Tou Jing Wai Ke Za Zhi. 2015;29(22):1977-80. PMid:26911062.

[204] Liu P, Chen P, Wang Z, Wei Y, Le W, Ding G, et al. Analysis of TEOAE and AABR hearing screening and follow-up in NICU. Lin Chuang Er Bi Yan Hou Tou Jing Wai Ke Za Zhi. 2014;28(10):705-7. PMid:25129970.

[205] Liu Z, Bu X, Xing G, Lu L. A preliminary study of a hearing screening model for newborn. Zhonghua Er Bi Yan Hou Ke Za Zhi. 2001;36(4):292-4. PMid:12762000.

[206] Lopes BM, Bueno CD, Didoné DD, Sleifer P. Comparison between click and ce-chirp® stimuli in neonatal hearing screening. J Hum Growth Dev. 2020;30(2):260-5. https://doi.org/10.7322/jhgd.v30.10375.

[207] Lotfi Y, Movallali G. A universal newborn hearing screening in Iran. Iran Rehabil J. 2007;5:8-11.

[208] Low WK, Pang KY, Ho LY, Lim SB, Joseph R. Universal newborn hearing screening in Singapore: the need, implementation and challenges. Ann Acad Med Singap. 2005;34(4):301-6. https://doi.org/10.47102/annals-acadmedsg.V34N4p301. PMid:15937570.

[209] Lowell EL. Guidelines for the early detection of auditory deficiency. Bol Oficina Sanit Panam. 1960;49:467-70. PMid:13763822.

[210] Magnani C, Bacchi G, Borghini AM, Delmonte D, Fava G, Occasio AM, et al. Universal newborn hearing screening: the experience of the University Hospital of Parma. Acta Biomed. 2015;86(3):273-7. PMid:26694155.

[211] Magnusson M, Persson K, Sundelin C. The effectiveness of routine health examinations at 2, 6, 9 and 12 months of age: experiences based on data from a Swedish county. Child Care Health Dev. 2001;27(2):117-31. https://doi.org/10.1046/j.1365-2214.2001.00180.x. PMid:11251611.

[212] Mahmood Z, Dogar MR, Waheed A, Ahmad AN, Anwar Z, Abbasi SZ, et al. Screening programs for hearing assessment in newborns and children. Cureus. 2020;12(11):e11284. https://doi.org/10.7759/cureus.11284. PMid:33274158.

[213] Maluleke NP, Khoza-Shangase K, Kanji A. Hearing impairment detection and intervention in children from centre-based early intervention programmes. J Child Health Care. 2019;23(2):232-41. https://doi.org/10.1177/1367493518788477. PMid:30068223.

[214] Maniuc M, Chiaburu-Chiosa D. Auditory function assessment via audiological screening in children. Proc Natl Ent Head Neck Surg Conf. 2018:145-50.

[215] Marco Algarra J, Pitarch Ribes MI, Morant Ventura A. Early diagnosis of neonatal hearing loss. Acta Otorrinolaringol Esp. 1996;47(3):255-7. PMid:8924295.

[216] Marinho ACA, de Souza Pereira EC, Torres KKC, Miranda AM, Ledesma ALL. Evaluation of newborn hearing screening program. Rev Saude Publica. 2020;54:44. https://doi.org/10.11606/s1518-8787.2020054001643. PMid:32374803.

[217] Marn B. First model of the newborn screening for hearing impairment in Croatia. Paediatr Croat. 2000;44:77-80.

[218] Marn B. Early detection of hearing impairment in croatia - screening and diagnostics. Paediatr Croat. 2012;56:195-201.

[219] Martines F, Porrello M, Ferrara M, Martines M, Martines E. Newborn hearing screening project using transient evoked otoacoustic emissions: western Sicily experience. Int J Pediatr Otorhinolaryngol. 2007;71(1):107-12. https://doi.org/10.1016/j.ijporl.2006.09.011. PMid:17095100.

[220] Martines F, Salvago P, Cocuzza S, La Mattina E, Stefano M, Mucia M, et al. Essential of audiology: screening and post-screening. Ital J Pediatr. 2014;40.

[221] Martínez R, Benito JI, Condado MA, Morais D, Fernández-Calvo JL. Results of the application of the protocol for the early detection of hearing loss in high-risk neonates. An Otorrinolaringol Ibero Am. 2003;30(3):277-87. PMid:12918292.

[222] Martínez R, Benito Orejas JI, Condado MA, Morais D, Fernández Calvo JL. Results of 1 year’s application of a universal protocol for the early detection of hearing loss in neonates. Acta Otorrinolaringologica Espanola. 2003;54(5):309-15. https://doi.org/10.1016/S0001-6519(03)78419-2. PMid:12916474.

[223] Martínez-Pacheco MC, Ferrán de la Cierva L, García-Purriños FJ. Delayed diagnosis of childhood deafness: the value of false negatives in the Programme for Early Detection of Neonatal Hearing Loss. Acta Otorrinolaringol Esp. 2016;67(6):324-9. https://doi.org/10.1016/j.otoeng.2016.01.001. PMid:27061391.

[224] Mason JA, Herrmann KR. Universal infant hearing screening by automated auditory brainstem response measurement. Pediatrics. 1998;101(2):221-8. https://doi.org/10.1542/peds.101.2.221. PMid:9445495.

[225] Mathur NN, Dhawan R. An alternative strategy for universal infant hearing screening in tertiary hospitals with a high delivery rate, within a developing country, using transient evoked oto-acoustic emissions and brainstem evoked response audiometry. J Laryngol Rhinol Otol. 2007;121(7):639-43. https://doi.org/10.1017/S0022215106004403. PMid:17112395.

[226] Matschke RG, Plath P. Early detection of hearing impairment - a simple method of screening of neonates. HNO. 1985;33:40-4. PMid:3972648.

[227] Matschke RG, Plath P. Early detection of hearing impairment. A simple method of screening of neonates. HNO. 1985;33:40-4. PMid:3972648.

[228] Mattos WM, Cardoso LF, Bissani C, Pinheiro MM, Viveiros CM, Carreirão Filho W. Newborn hearing screening program implantation analysis at a University Hospital. Braz J Otorhinolaryngol. 2009;75(2):237-44. https://doi.org/10.1016/S1808-8694(15)30784-9. PMid:19575110.

[229] Mauk GW, White KR, Mortensen LB, Behrens TR. The effectiveness of screening programs based on high-risk characteristics in early identification of hearing impairment. Ear Hear. 1991;12(5):312-9. https://doi.org/10.1097/00003446-199110000-00003. PMid:1783234.

[230] Maxon AB, White KR, Vohr BR, Behrens TR. Using transient evoked otoacoustic emissions for neonatal hearing screening. Br J Audiol. 1993;27(2):149-53. https://doi.org/10.3109/03005369309077906. PMid:8220282.

[231] Meier S, Narabayashi O, Probst R, Schmuziger N. Comparison of currently available devices designed for newborn hearing screening using automated auditory brainstem and/or otoacoustic emission measurements. Int J Pediatr Otorhinolaryngol. 2004;68(7):927-34. https://doi.org/10.1016/j.ijporl.2004.02.008. PMid:15183584.

[232] Mencher GT. A program for neonatal hearing screening. Audiology. 1974;13(6):495-500. https://doi.org/10.3109/00206097409071713. PMid:4411714.

[233] Meyer ME, Swanepoel DW, le Roux T, van der Linde M. Early detection of infant hearing loss in the private health care sector of South Africa. Int J Pediatr Otorhinolaryngol. 2012;76(5):698-703. https://doi.org/10.1016/j.ijporl.2012.02.023. PMid:22386272.

[234] Mijares Nodarse E, Gaya Vázquez JA, Savio López G, Pérez Abalo MC, Eimil Suárez E, Torres Fortuny A. Diagnostics tests more used in the early detection of hearing losses. Rev Logop Foniatr Audiol. 2006;26:91-100. https://doi.org/10.1016/S0214-4603(06)70107-3.

[235] Molini E, Ricci G, Simoncelli C, Alunni N, Capolynghi B, Giommetti S, et al. Click evoked otoacoustic emissions (EOAES) to screen hearing in neonates. Rev Laryngol Otol Rhinol (Bord). 1996;117:341-3. PMid:9099021.

[236] Molteni G. Early detection of newborn hearing impairment by transiently evoked otoacoustic emissions and auditory evoked potentials. Personal experience in 10,454 children. Otorinolaringologia. 2006;56:93-6.

[237] Monteiro L. Auditory screening of newborns and children. Nascer Crescer. 2004;13:S283-9.

[238] Montovani JC, Fioravanti MP, Tamashiro IA. Avaliaçäo auditiva em recém-nascido e lactente. Rev Paul Pediatr. 1996;14:78-83.

[239] Moradi-Joo E, Barouni M, Vali L, Mahmoudian S. Qualitative Analysis of Newborn Hearing Screening Program in Iran. Med J Islam Repub Iran. 2024;38:146-54. https://doi.org/10.47176/mjiri.38.23. PMid:38783982.

[240] Morgan DE, Canalis RF. Auditory screening of infants. Otolaryngol Clin North Am. 1991;24(2):277-84. https://doi.org/10.1016/S0030-6665(20)31139-7. PMid:1907005.

[241] Mukkun T. The result of universal newborn hearing screening, 4 years of experience in trang. J Med Assoc Thai. 2021;104(1):95-9. https://doi.org/10.35755/jmedassocthai.2021.01.11432.

[242] Murray AD, Javel E, Watson CS. Prognostic validity of auditory brainstem evoked response screening in newborn infants. Am J Otolaryngol. 1985;6(2):120-31. https://doi.org/10.1016/S0196-0709(85)80050-8. PMid:3887960.

[243] Navarro Rivero B, Gonzalez Dîaz E, Marrero Santos L, Martinez Toledano I, Murillo Diaz MJ, Valino Colas MJ. Brainstem auditory evoked responses in children at risk: a prospective study. An Esp Pediatr. 1999;50:357-60. PMid:10356827.

[244] Nennstiel-Ratzel U, Arenz S, Von Kries R, Wildner M, Strutz J. The model project “newborn auditory screening” in the Upper Palatinate. High process and result quality of an interdisciplinary concept. HNO. 2007;55(2):128-34. https://doi.org/10.1007/s00106-006-1383-x. PMid:16528506.

[245] Newton VE, Liu X, Ke X, Xu L, Bamford JM. Evaluation of the use of a questionnaire to detect hearing loss in babies in China. Int J Pediatr Otorhinolaryngol. 1999;48(2):125-9. https://doi.org/10.1016/S0165-5876(99)00016-6. PMid:10375037.

[246] Ng J, Yun HL. Otoacoustic emissions (OAE) in paediatric hearing screening–the Singapore experience. J Singapore Paediatr Soc. 1992;34(1-2):1-5. PMid:1303456.

[247] Ng PK, Hui Y, Lam BCC, Goh WHS, Yeung CY. Feasibility of implementing a universal neonatal hearing screening programme using distortion product otoacoustic emission detection at a university hospital in Hong Kong. Hong Kong Med J. 2004;10(1):6-13. PMid:14967849.

[248] Norton SJ, Gorga MP, Widen JE, Folsom RC, Sininger Y, Cone-Wesson B, et al. Identification of Neonatal Hearing Impairment: evaluation of transient evoked otoacoustic emission, distortion product otoacoustic emission, and auditory brain stem response test performance. Ear Hear. 2000;21(5):508-28. https://doi.org/10.1097/00003446-200010000-00013. PMid:11059707.

[249] Norton SJ, Gorga MP, Widen JE, Vohr BR, Folsom RC, Sininger YS, et al. Identification of Neonatal Hearing Impairment: transient evoked otoacoustic emissions during the perinatal period. Ear Hear. 2000;21(5):425-42. https://doi.org/10.1097/00003446-200010000-00008. PMid:11059702.

[250] Obeidat FS, Alothman N, Alkahtani R, Al-Najjar S, Obeidat M, Ali AY, et al. Evaluation of newborn hearing screening program in Jordan. Front Pediatr. 2024;12:1420678. https://doi.org/10.3389/fped.2024.1420678. PMid:39055617.

[251] Olarte M, Bermúdez Rey MC, Beltran AP, Guerrero D, Suárez-Obando F, López G, et al. Detection of hearing loss in newborns: definition of a screening strategy in Bogotá, Colombia. Int J Pediatr Otorhinolaryngol. 2019;122:76-81. https://doi.org/10.1016/j.ijporl.2019.03.016. PMid:30978473.

[252] Olusanya B. Early detection of hearing impairment in a developing country: what options? Audiology. 2001;40(3):141-7. https://doi.org/10.3109/00206090109073109. PMid:11465296.

[253] Olusanya BO, Ebuehi OM, Somefun AO. Universal infant hearing screening programme in a community with predominant non-hospital births: A three-year experience. J Epidemiol Community Health. 2009;63(6):481-7. https://doi.org/10.1136/jech.2008.082784. PMid:19228683.

[254] Onoda RM, Azevedo MF, dos Santos AMN. Triagem auditiva neonatal: ocorrência de falhas, perdas auditivas e indicadores de riscos. Braz J Otorhinolaryngol. 2011;77(6):775-83. https://doi.org/10.1590/S1808-86942011000600015.

[255] Paat-Ahi G, Sikkut R, Laarmann H. Evaluation of the new born hearing screening programme. Eesti Arst. 2014;93:571-7.

[256] Pastorino G, Sergi P, Mastrangelo M, Ravazzani P, Tognola G, Parazzini M, et al. The Milan Project: a newborn hearing screening programme. Acta Paediatr. 2005;94(4):458-63. https://doi.org/10.1111/j.1651-2227.2005.tb01918.x. PMid:16092461.

[257] Paul AK. Hearing loss in children: need for early detection and intervention. Indian J Pract Pediatr. 2003;5:353-5.

[258] Pedersen L, Møller TR, Wetke R, Ovesen T. Neonatal hearing screening - Transient-evoked oto-acoustic emission compared to automatic auditory brainstem response. Ugeskr Læger. 2008;170:642-6. PMid:18364157.

[259] Pelosi G, Hatzopoulos S, Chierici R, Vigi V, Martini A. Evaluation of a linear TEOAE protocol in hearing screening of neonates: feasibility study. Acta Otorhinolaryngol Ital. 1998;18(4):213-7. PMid:10205919.

[260] Pitathawatchai P, Khaimook W, Kirtsreesakul V. Pilot implementation of newborn hearing screening programme at four hospitals in southern Thailand. Bull World Health Organ. 2019;97(10):663-71. https://doi.org/10.2471/BLT.18.220939. PMid:31656331.

[261] Pitathawatchai P, Wanachottrakul P, Prayuenyong P. Education on loss to follow-up after newborn hearing screening in Thailand: a randomised controlled trial. Int J Audiol. 2025;64(11):1204-10. https://doi.org/10.1080/14992027.2025.2496238. PMid:40266260.

[262] Plinkert PK, Delb W. Universal auditory screening in a German state: a multidisciplinary project. HNO. 2001;49:888-94. https://doi.org/10.1007/s001060170013. PMid:11759240.

[263] Plinkert PK, Delb W. Electronic data processing-assisted organization of interdisciplinary universal hearing screening in Saarland. HNO. 2001;49:888-94. https://doi.org/10.1007/s001060170013.

[264] Plinkert PK, Sesterhenn G, Arold R, Zenner HP. Evaluation of otoacoustic emissions in high-risk infants by using an easy and rapid objective auditory screening method. Eur Arch Oto-Rhino-Laryngol. 1990;247(6):356-60. https://doi.org/10.1007/BF00179006. PMid:2278701.

[265] Poblano A, Chayo I, Ibarra J, Rueda E. Electrophysiological and behavioral methods in early detection of hearing impairment. Arch Med Res. 2000;31(1):75-80. https://doi.org/10.1016/S0188-4409(99)00060-0. PMid:10767484.

[266] Polski B, Szydłowski J, Pucher B, Sroczyński J. The GP’s role in the early detection of hearing loss process, realised by the universal neonatal hearing screenings of infants program. Family Medicine and Primary Care Review. 2014;16:148-9.

[267] Poncet E, Chaperon C. Results of a ten-year screening programme for the diagnosis of deafness and its consequences in children. Early education of the deaf infant. Ann Otolaryngol Chir Cervicofac. 1983;100:543-8. PMid:6670806.

[268] Prieve BA, Stevens F. The New York State universal newborn hearing screening demonstration project: introduction and overview. Ear Hear. 2000;21(2):85-91. https://doi.org/10.1097/00003446-200004000-00003. PMid:10777016.

[269] Prieve BA, Vander Werff KR, Preston JL, Georgantas L. Identification of conductive hearing loss in young infants using tympanometry and wideband reflectance. Ear Hear. 2013;34(2):168-78. https://doi.org/10.1097/AUD.0b013e31826fe611. PMid:23263407.

[270] Prince CB, Miyashiro L, Weirather Y, Heu P. Epidemiology of early hearing loss detection in Hawaii. Pediatrics. 2003;111(5 Pt 2, Suppl 1):1202-6. https://doi.org/10.1542/peds.111.S1.1202. PMid:12728139.

[271] Ptok M. Early diagnosis of hearing impairment in children. Z Arztl Fortbild Qualitatssich. 2004;98(4):265-70. PMid:15295927.

[272] Radü HJ. Comparison of different audiometric procedures for infants less than 1 year old. HNO. 1985;33(6):271-4. PMid:4030410.

[273] Raghuwanshi SK, Gargava A, Kulkarani V, Kumar A. Role of otoacoustic emission test in neonatal screening at Tertiary Center. Indian J Otolaryngol Head Neck Surg. 2019;71(Suppl 2):1535-7. https://doi.org/10.1007/s12070-019-01606-0. PMid:31750212.

[274] Appanaitis I, Rahimian R. Early hearing detection and intervention in the Republic of Palau: program successes and challenges. J Investig Med. 2018;66(1):A106-7. https://doi.org/10.1136/jim-2017-000663.100.

[275] Reis FMFS, Gonçalves CGO, Conto J, Iantas M, Lüders D, Marques J. Hearing assessment of neonates at risk for hearing loss at a hearing health high complexity service: an electrophysiological assessment. Int Arch Otorhinolaryngol. 2019;23(2):157-64. https://doi.org/10.1055/s-0038-1648217. PMid:30956699.

[276] Ren B, Zhang X, Zhang Q, Gan W, Zhang Y, Guo B, et al. Associational study of neonatal hearing screening results and common metabolic disorders. Int J Pediatr Otorhinolaryngol. 2025;196:112489. https://doi.org/10.1016/j.ijporl.2025.112489. PMid:40690833.

[277] Richard C, Shakhtour L, Gallo N, Smith R, MacDonald CB, Gentry R, et al. Audiological outcomes of cytomegalovirus saliva PCR-Positive Newborns in support of universal screening. Otolaryngol Head Neck Surg. 2025;173(5):1245-53. https://doi.org/10.1002/ohn.1332. PMid:40504068.

[278] Rodrigues RP. Avaliação da implantação do Programa de Triagem Auditiva Neonatal em maternidades públicas brasileiras [dissertação]. Rio de Janeiro: Fundação Oswaldo Cruz, Instituto Nacional de Saúde da Mulher, da Criança e do Adolescente Fernandes Figueira; 2020 [citado em 2026 maio 18]. Disponível em: https://acervos.icict.fiocruz.br/iff/mestrado_bibsmc/renata_rodrigues_iff_mest_2020.pdf

[279] Roman S, Mondain M, Triglia JM, Uziel A. Auditory screening in neonates: evoked oto-acoustic emissions or acoustic distortion product? Rev Laryngol Otol Rhinol (Bord). 2001;122(3):155-8. PMid:11799854.

[280] Ruiz De La Cuesta F, Juste Ruiz M, Cortés Castell E. Results of applying for 13 years the universal newborn hearing screening protocol and study of cases that do not pass the screening. Acta Pediatr Esp. 2018;76:77-82.

[281] Saitoh Y, Hazama M, Sakoda T, Hamada H, Ikeda H, Seno S, et al. Outcome of neonatal screening for hearing loss in neonatal intensive care unit and well-born nursery infants. Nippon Jibiinkoka Gakkai Kaiho. 2002;105(12):1205-11. https://doi.org/10.3950/jibiinkoka.105.1205. PMid:12607282.

[282] Saki N, Bayat A, Hoseinabadi R, Nikakhlagh S, Karimi M, Dashti R. Universal newborn hearing screening in southwestern Iran. Int J Pediatr Otorhinolaryngol. 2017;97:89-92. https://doi.org/10.1016/j.ijporl.2017.03.038. PMid:28483258.

[283] Santos-Ceballos JC, Pérez-Blanco JG, Pantoja-Gómez Y, Martín-González F, Torres-Fortuny A, Eimil-Suarez E, et al. Implementation of the NEURONIC INFANTIX Newborn Hearing Screening System. IFMBE Proc. 2020;75:453-9. https://doi.org/10.1007/978-3-030-30648-9_59.

[284] Sarno CN, Clemis JD. A workable approach to the identification of neonatal hearing impairment. Laryngoscope. 1980;90(8 Pt 1):1313-20. https://doi.org/10.1288/00005537-198008000-00008. PMid:7401831.

[285] Sass-Lehrer M. Early detection of hearing loss: maintaining a family-centered perspective. Semin Hear. 2004;25(4):295-307. https://doi.org/10.1055/s-2004-836132.

[286] Sassada MMY, Ceccon MEJ, de Navarro JM, Vaz FAC. Avaliação auditiva de recém-nascidos gravemente enfermos através do método de emissões otoacústicas evocadas transientes (EOAT) e audiometria de tronco cerebral(BERA). Pediatria (Säo Paulo). 2005;27:154-62.

[287] Satish HS, Anil Kumar R, Viswanatha B. Screening of Newborn Hearing at a Tertiary Care Hospital in South India. Indian J Otolaryngol Head Neck Surg. 2019;71(Suppl 2):1383-90. https://doi.org/10.1007/s12070-018-1454-9. PMid:31750182.

[288] Schade G. Early detection of hearing disorders. Laryngorhinootologie. 2008;87(Suppl 1):S21-31. https://doi.org/10.1055/s-2007-995550. PMid:18373306.

[289] Schauseil-Zipf U, Von Wedel H. Hearing screening by means of acoustic evoked brain stem potentials in newborns and infants. Klin Padiatr. 1988;200(4):324-9. https://doi.org/10.1055/s-2008-1033729. PMid:3172672.

[290] Schmidt P, Leveque M, Danvin JB, Leroux B, Chays A. Systematic hearing screening for newborns in the Champagne-Ardennes region: 32,500 births in 2 years of experience. Ann Otolaryngol Chir Cervicofac. 2007;124(4):157-65. https://doi.org/10.1016/j.aorl.2006.10.004. PMid:17669353.

[291] Schönweiler R, Tioutou E, Tolloczko R, Pankau R, Ptok M. Hearing screening with automatic evaluation of TEOAE and a new method of automatic evaluation of early auditory evoked potentials. Optimization and field trial. HNO. 2002;50:649-56. https://doi.org/10.1007/s00106-002-0636-6.

[292] Sedano MC, San Martín UA, Rahal EM. Realidad nacional de los programas de detección auditiva temprana con miras a la cobertura universal. Rev Otorrinolaringol Cir Cabeza Cuello. 2018;78(1):9-14. https://doi.org/10.4067/s0717-75262018000100009.

[293] Seguya A, Bajunirwe F, Kakande E, Nakku D. Feasibility of establishing an infant hearing screening program and measuring hearing loss among infants at a regional referral hospital in south western Uganda. PLoS One. 2021;16(6):e0253305. https://doi.org/10.1371/journal.pone.0253305. PMid:34138954.

[294] Sente M, Aleksov-Hatvan G. Early detection of hearing impairment in children. Srp Arh Celok Lek. 2006;134(9-10):448-52. PMid:17252916.

[295] Shang Y, Hao W, Gao Z, Xu C, Ru Y, Ni D. An effective compromise between cost and referral rate: a sequential hearing screening protocol using TEOAEs and AABRs for healthy newborns. Int J Pediatr Otorhinolaryngol. 2016;91:141-5. https://doi.org/10.1016/j.ijporl.2016.10.025. PMid:27863628.

[296] Sharma R, Gu Y, Sinha K, Ching TYC, Marnane V, Gold L, et al. An Economic Evaluation of Australia’s Newborn Hearing Screening Program: A Within-Study Cost-Effectiveness Analysis. Ear Hear. 2022;43(3):972-83. https://doi.org/10.1097/AUD.0000000000001153. PMid:34772837.

[297] Shehata-Dieler WE, Dieler R, Keim R, Finkenzeller P, Dietl J, Helms J. Universal hearing screening in newborns using the CRESCENDO newborn hearing screener. Laryngorhinootologie. 2000;79:69-76. https://doi.org/10.1055/s-2000-8792. PMid:10738712.

[298] Shi Y, Zhang N, Du N, Zheng T, Yu Y, Li Y. Genetic and audiological determinants of hearing loss in high-risk neonates. Braz J Otorhinolaryngol. 2025;91(2):101541. https://doi.org/10.1016/j.bjorl.2024.101541. PMid:39754783.

[299] Shirane M, Ganaha A, Nakashima T, Shimoara S, Yasunaga T, Ichihara S, et al. Comprehensive hearing care network for early identification and intervention in children with congenital and late-onset/acquired hearing loss: 8 years’ experience in Miyazaki. Int J Pediatr Otorhinolaryngol. 2020;131:109881. https://doi.org/10.1016/j.ijporl.2020.109881. PMid:31978747.

[300] Shulman S, Besculides M, Saltzman A, Ireys H, White KR, Forsman I. Evaluation of the universal newborn hearing screening and intervention program. Pediatrics. 2010;126(Suppl 1):S19-27. https://doi.org/10.1542/peds.2010-0354F. PMid:20679316.

[301] Solanellas Soler J. Hearing loss: early hearing detection and intervention. Pediatria Integral. 2009;13:457-67.

[302] Sun JH, Li J, Huang P, Bu J, Xu ZM, Li J, et al. Early detection of hearing impairment in high-risk infants of NICU. Zhonghua Er Ke Za Zhi. 2003;41(5):357-9. PMid:14751056.

[303] Sun X, Shen X, Zakus D, Lv J, Xu Z, Wu H, et al. Development of an effective public health screening program to assess hearing disabilities among newborns in Shanghai: a prospective cohort study. World Health Popul. 2009;11(1):14-23. PMid:20039591.

[304] Sun X, Xi Z, Zhang J, Liu B, Xing X, Huang X, et al. Combined hearing and deafness gene mutation screening of 11 046 Chinese newborns. Zhonghua Yi Xue Yi Chuan Xue Za Zhi. 2015;32(6):766-70. https://doi.org/10.3760/cma.j.issn.1003-9406.2015.06.002. PMid:26663044.

[305] Sun XY, Ding J, Zhang Y, Xu Y. Analysis of hearing screening results of 268 187 newborns in Jiaxing from 2013 to 2017. Zhongguo Ertong Baojian Zazhi. 2019;27:453-6. https://doi.org/10.11852/zgetbjzz2018-0999.

[306] Swanepoel DCD, Delport SD, Swart JG. Universal newborn hearing screening in South Africa - A first-world dream? S Afr Med J. 2004;94(8):634-5. PMid:15352584.

[307] Szyfter W, Greczka G, Dąbrowski P, Wróbel M. The report on the Universal Neonatal Hearing Screening Program in Poland between 2003 and 2015. Otolaryngol Pol. 2016;70(2):1-5. https://doi.org/10.5604/00306657.1199346. PMid:27386827.

[308] Szyfter W, Wróbel M, Radziszewska-Konopka M, Szyfter-Harris J, Karlik M. Polish Universal Neonatal Hearing Screening Program-4-year experience (2003-2006). Int J Pediatr Otorhinolaryngol. 2008;72(12):1783-7. https://doi.org/10.1016/j.ijporl.2008.08.015. PMid:18922586.

[309] Tanon-Anoh MJ, Sanogo-Gone D, Kouassi KB. Newborn hearing screening in a developing country: results of a pilot study in Abidjan, Côte d’ivoire. Int J Pediatr Otorhinolaryngol. 2010;74(2):188-91. https://doi.org/10.1016/j.ijporl.2009.11.008. PMid:19963282.

[310] Tham EH, Lee LY, Joseph R. Evaluation of newborn screening practices among paediatricians practising in the private sector in Singapore. Ann Acad Med Singap. 2010;39:S168.

[311] Todd NW. Universal newborn hearing screening follow-up in two Georgia populations: newborn, mother and system correlates. Int J Pediatr Otorhinolaryngol. 2006;70(5):807-15. https://doi.org/10.1016/j.ijporl.2005.09.019. PMid:16246432.

[312] Trinidad Ruiz G, Pantoja Hernández CG, Trinidad Ramos G, Serrano Berrocal MA, Pardo Romero G, González Palomino A, et al. Controlling retests in a universal hearing screening program. Acta Otorrinolaringologica Espanola. 2005;56(3):96-101. https://doi.org/10.1016/S0001-6519(05)78580-0. PMid:15819515.

[313] Tufatulin GS, Lalayants MR, Artyushkin SA, Vikhnina SM, Garbaruk ES, Dvoryanchikov VV, et al. Clinical protocol: audiological assessment of infants in Russian Federation. Part II. Vestn Otorinolaringol 2023;88(6):81-90. https://doi.org/10.17116/otorino20238806181.

[314] Turan Z, Bas N. Evaluation of the effectiveness of newborn hearing screening program: a center in Turkey. Int J Early Child Spec Educ. 2019;11:141-53. https://doi.org/10.20489/intjecse.670470.

[315] Tzanakakis MG, Chimona TS, Apazidou E, Giannakopoulou C, Velegrakis GA, Papadakis CE. Transitory evoked otoacoustic emission (TEOAE) and distortion product otoacoustic emission (DPOAE) outcomes from a three-stage newborn hearing screening protocol. Hippokratia. 2016;20(2):104-9. PMid:28416905.

[316] Ulusoy S, Ugras H, Cingi C, Yilmaz HB, Muluk NB. The results of national newborn hearing screening (NNHS) data of 11,575 newborns from west part of Turkey. Eur Rev Med Pharmacol Sci. 2014;18(20):2995-3003. PMid:25392094.

[317] Ulusoy S, Uʇraş H, Cingi C, Yilmaz HB. Analysis of hearing screening test findings of 11,575 newborns in Turkey. OHNS. 2013;149(S2):240-1. https://doi.org/10.1177/0194599813496044a299.

[318] Uppenkamp S, Jäkel M, Talartschick B, Büschel J, Kollmeier B. Evoked otoacoustic emissions as a screening test for hearing evaluation in newborn and premature infants?. Laryngorhinootologie 1992;71(10):525-9. https://doi.org/ 10.1055/s-2007-997347.

[319] Valencia DM, Rimell FL, Friedman BJ, Oblander MR, Helmbrecht J. Cochlear implantation in infants less than 12 months of age. Int J Pediatr Otorhinolaryngol. 2008;72(6):767-73. https://doi.org/10.1016/j.ijporl.2008.02.009. PMid:18403026.

[320] Van Straaten HLM, Groote ME, Oudesluys-Murphy AM. Evaluation of an automated auditory brainstem response infant hearing screening method in at risk neonates. Eur J Pediatr. 1996;155(8):702-5. https://doi.org/10.1007/BF01957157. PMid:8839729.

[321] Van Straaten HL, Tibosch CH, Dorrepaal C, Dekker FW, Kok JH. Efficacy of automated auditory brainstem response hearing screening in very preterm newborns. J Pediatr. 2001;138(5):674-8. https://doi.org/10.1067/mpd.2001.112646. PMid:11343042.

[322] Vernier LS, Da Rocha BSC, Reppold CT, Zanini CFC, Levandowski DC, De Carvalho Paniz T. Which is the best time to perform the newborn hearing screening? Preliminary results of a study conducted in a public hospital of Porto Alegre. Int Arch Otorhinolaryngol. 2014;18:a2297. https://doi.org/10.1055/s-0034-1389006.

[323] Verrue N, Smets K. Possibilities and pitfalls of early neonatal hearing screening with automated auditory brainstem response measurements in a neonatal intensive care unit. Tijdschr Geneeskd. 2012;68:497-504. https://doi.org/10.2143/TVG.68.10.2001188.

[324] Vila C, Demestre X, Sagrera X, Sala P, Raspall F. Newborn hearing screening. Pediatria Catalana. 2004;64:20-4.

[325] Voegeli R. Early detection of hearing impairment in childhood. Therapeutische Umschau. 1979;36(9):788-91. PMid:505326.

[326] Vohr B, Simon P, McDermott C, Kurtzer-White E, Johnson MJ, Topol D. Early hearing screening, detection and intervention (EHDI) in Rhode Island. Med Health R I. 2002;85(12):369-72. PMid:12593355.

[327] Vos B, Lagasse R, Levêque A. The organisation of universal newborn hearing screening in the Wallonia-Brussels Federation. B-ENT. 2013;(Suppl 21):9-15. PMid:24383218.

[328] Vranješ D, Spremo S, Travar D, Aleksić A, Novaković Z, Stevandić N, et al. The role and importance of screening procedures in early diagnostics of hearing impairment. Medicinski Casopis. 2012;46(2):71-6. https://doi.org/10.5937/mckg46-1265.

[329] Wang DY, Xing GQ, Liu ZY. Transiently evoked otoacoustic emissions for universal newborn hearing screening. Zhongguo Linchuang Kangfu. 2002;6:2210-1.

[330] Wang J, Chen W, Wang J, Li J, Zhao Y, Chen P. Evaluation of the effect of combined otoacoustic emission and automatic auditory EEG response in neonatal hearing screening. Minerva Pediatr (Torino). 2022;74(2):239-41. https://doi.org/10.23736/S2724-5276.21.06576-9. PMid:34530588.

[331] Watkin PM. Controlling the quality of universal neonatal hearing screens. Public Health. 1999;113(4):171-6. https://doi.org/10.1016/S0033-3506(99)00148-1. PMid:10483078.

[332] Weichbold V, Nekahm-Heis D, Welzl-Müller K. Evaluation of the Austrian Newborn Hearing Screening Program. Wien Klin Wochenschr. 2005;117(18):641-6. https://doi.org/10.1007/s00508-005-0414-z. PMid:16416347.

[333] Welzl-Müller K, Stephan K. Examples of implemented neonatal hearing screening programs in Austria. Scand Audiol Suppl. 2001;30(52):7-9. https://doi.org/10.1080/010503901300006912. PMid:11318488.

[334] Widen JE, Folsom RC, Cone-Wesson B, Carty L, Dunnell JJ, Koebsell K, et al. Identification of Neonatal Hearing Impairment: hearing status at 8 to 12 months corrected age using a visual reinforcement audiometry protocol. Ear Hear. 2000;21(5):471-87. https://doi.org/10.1097/00003446-200010000-00011. PMid:11059705.

[335] Wong YA, Mazlan R, Abdul Wahab NA, Ja’afar R, Huda Bani N, Abdullah NA. Quality measures of a multicentre universal newborn hearing screening program in Malaysia. J Med Screen. 2021;28(3):238-43. https://doi.org/10.1177/0969141320973060. PMid:33202173.

[336] Wu JL, Huang CC, Kao CC. A hearing screening in very low birth weight preterm infants by auditory brainstem response. J Otolaryngol Soc Repub China. 1998;33:212-8.

[337] Xu J, Chen S, Zheng Z, Xu M, Shen A. [Follow-up examination for newborns and infants who failed hearing screening]. Lin Chuang Er Bi Yan Hou Tou Jing Wai Ke Za Zhi. 2006;20:446-8.

[338] Xu Z, Li J. Performance of two hearing screening protocols in the NICU. B-ENT. 2005;1(1):11-5. PMid:15999670.

[339] Xu ZM, Cheng WX, Yang XL. Performance of two hearing screening protocols in NICU in Shanghai. Int J Pediatr Otorhinolaryngol. 2011;75(10):1225-9. https://doi.org/10.1016/j.ijporl.2011.07.004. PMid:21802153.

[340] Xu Z, Li J. Performance of two hearing screening protocols in the NICU. B-ENT. 2005;1(1):11-5. PMid:15999670.

[341] Xu ZM, Li J, Hu TZ, Sun JH, Shen XM. Sensitivity of distortion product otoacoustic emissions and auditory brain-stem response in neonatal hearing screening, a comparative study. Zhonghua Yi Xue Za Zhi. 2003;83(4):278-80. PMid:12812641.

[342] Yao GD, Li SX, Chen DL, Feng HQ, Zhao SB, Liu YJ, et al. Combination of hearing screening and genetic screening for deafness-susceptibility genes in newborns. Exp Ther Med. 2013;7(1):218-22. https://doi.org/10.3892/etm.2013.1406. PMid:24348793.

[343] Yigit Ö, Batuk M, Çinar B, Yildirim M, Yarali M, Sennaroglu G. Auditory brainstem response measurements in newborns: which electrode placement is better? Turk Arch Otorhinolaryngol. 2020;58(2):112-7. https://doi.org/10.5152/tao.2020.5065. PMid:32783038.

[344] Yılmazer R, Yazıcı MZ, Erdim İ, Kaya HK, Özcan Dalbudak Ş, Kayhan TF. Follow-up results of newborns after hearing screening at a training and Research Hospital in Turkey. J Int Adv Otol. 2016;12(1):55-60. https://doi.org/10.5152/iao.2015.1736. PMid:27340984.

[345] Yoshinaga-Itano C. Universal newborn hearing screening programs and developmental outcomes. Audiol Med. 2003;1(3):199-206. https://doi.org/10.1080/16513860310002031.

[346] Yoshinaga-Itano C, Apuzzo MRL. Identification of hearing loss after age 18 months is not early enough. Am Ann Deaf. 1998;143(5):380-7. https://doi.org/10.1353/aad.2012.0151. PMid:9893323.

[347] Yoshinaga-Itano C, Gravel JS. The evidence for universal newborn hearing screening. Am J Audiol. 2001;10(2):62-4. https://doi.org/10.1044/1059-0889(2001/013). PMid:11808721.

[348] Yousefi J, Ajalloueyan M, Amirsalari S, Fard MH. The specificity and sensitivity of transient otoacustic emission in neonatal hearing screening compared with diagnostic test of auditory brain stem response in Tehran hospitals. Iran J Pediatr. 2013;23(2):199-204. PMid:23724183.

[349] Yousefi J, Ajalloueyan M, Amirsalari S, Hassanali Fard M. The specificity and sensitivity of transient otoacustic emission in neonatal hearing screening compared with diagnostic test of auditory brain stem response in tehran hospitals. Iran J Pediatr. 2013;23(2):199-204. PMid:23724183.

[350] Zeng X, Liu Z, Wang J, Zeng X. Combined hearing screening and genetic screening of deafness among Hakka newborns in China. Int J Pediatr Otorhinolaryngol. 2020;136:110120. https://doi.org/10.1016/j.ijporl.2020.110120. PMid:32574949.

[351] Zhang Y, Li G, Zheng Y. Early diagnosis and intervention in 0-9 months old infants with hearing loss. Lin Chuang Er Bi Yan Hou Tou Jing Wai Ke Za Zhi. 2014;28(22):1748-51. PMid:25752104.

[352] Zhang Z, Liu Y, Xie LS, Jia DQ, Gao PM, Luo RZ. Establishment and procedure of universal newborn hearing screening. Zhongguo Linchuang Kangfu. 2004;8:2304-6.

[353] Zhao PJ, Xu ZM, Wu SH, Jin CH, Yu H, Shen P, et al. Hearing screening for high-risk newborns. Zhonghua Yi Xue Za Zhi. 2003;83(4):285-8. PMid:12812643.

[354] Zwicker E, Schorn K. Delayed evoked otoacoustic emissions - an ideal screening-test for excluding hearing impairment in infants. Audiology. 1990;29(5):241-51. https://doi.org/10.3109/00206099009072855. PMid:2275639.

[355] Zych M, Greczka G, Dąbrowski P, Wróbel M, Szyfter-Harris J, Szyfter W. The report of the polish universal neonatal hearing screening program in 2016. Otolaryngol Pol. 2018;72(1):1-4. https://doi.org/10.5604/01.3001.0011.5913. PMid:29513258.
